# Transmembrane Shuttling of Photosynthetically Produced Electrons to Propel Extracellular Biocatalytic Redox Reactions in a Modular Fashion

**DOI:** 10.1002/ange.202207971

**Published:** 2022-08-29

**Authors:** Valentina Jurkaš, Florian Weissensteiner, Piera De Santis, Stephan Vrabl, Frieda A. Sorgenfrei, Sarah Bierbaumer, Selin Kara, Robert Kourist, Pramod P. Wangikar, Christoph K. Winkler, Wolfgang Kroutil

**Affiliations:** ^1^ Institute of Chemistry University of Graz Heinrichstraße 28 8010 Graz Austria; ^2^ Department of Engineering, Biological and Chemical Engineering Section Biocatalysis and Bioprocessing Group Aarhus University Gustav Wieds Vej 10 8000 Aarhus Denmark; ^3^ Austrian Centre of Industrial Biotechnology, c/o Institute of Chemistry, University of Graz Heinrichstraße 28 8010 Graz Austria; ^4^ Institute of Molecular Biotechnology Graz University of Technology Petersgasse 14 8010 Graz Austria; ^5^ Department of Chemical Engineering, Indian Institute of Technology Bombay, Powai, Mumbai 400076 India DBT-Pan IIT Centre for Bioenergy, Indian Institute of Technology Bombay, Powai, Mumbai 400076 India Wadhwani Research Centre for Bioengineering Indian Institute of Technology Bombay Powai Mumbai 400076 India; ^6^ Field of Excellence BioHealth— University of Graz 8010 Graz Austria; ^7^ BioTechMed Graz 8010 Graz Austria

**Keywords:** Biocatalysis, Photocatalysis, Redox Chemistry, Reductions, Transmembrane Shuttling

## Abstract

Many biocatalytic redox reactions depend on the cofactor NAD(P)H, which may be provided by dedicated recycling systems. Exploiting light and water for NADPH‐regeneration as it is performed, e.g. by cyanobacteria, is conceptually very appealing due to its high atom economy. However, the current use of cyanobacteria is limited, e.g. by challenging and time‐consuming heterologous enzyme expression in cyanobacteria as well as limitations of substrate or product transport through the cell wall. Here we establish a transmembrane electron shuttling system propelled by the cyanobacterial photosynthesis to drive extracellular NAD(P)H‐dependent redox reactions. The modular photo‐electron shuttling (MPS) overcomes the need for cloning and problems associated with enzyme‐ or substrate‐toxicity and substrate uptake. The MPS was demonstrated on four classes of enzymes with 19 enzymes and various types of substrates, reaching conversions of up to 99 % and giving products with >99 % optical purity.

## Graphical abstract

Water is utilized as benign electron‐donor in photosynthesis, e.g. by cyanobacteria. Herein, a novel approach to shuttle them through the cyanobacterium's cell‐membrane and to utilize them for extracellular NAD(P)H‐dependent redox reactions is presented. The concept was demonstrated for 19 different reductive and oxidative enzymes from 4 different classes.

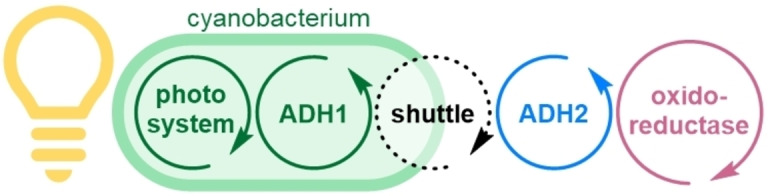

## Introduction

Stereoselective redox reactions belong to the most important transformations in organic synthesis.[^1^, ^2^, ^3^, ^4^] Typical examples are the asymmetric reductions of prochiral functional groups such as imines, ketones, or alkenes as well as stereoselective oxidations like C−H functionalization reactions or Baeyer–Villiger oxidations of selected molecules. Biocatalytic methods offer a broad spectrum of transformations for this portfolio of redox reactions,[^5^, ^6^, ^7^, ^8^] as about one quarter of the known enzymes belong to the class of oxidoreductases.[^9^, ^10^] The high stereoselectivity that biocatalysts usually exhibit is therefore mirrored by the broad application of biocatalytic oxidations and reductions in industry, especially in the synthesis of small molecule pharmaceuticals.[^8^, ^11^, ^12^, ^13^, ^14^]

Most biocatalytic reductions require one equivalent of reduced nicotinamide cofactor (NADH or NADPH) as electron‐source.^[1]^ In the case of monooxygenases, one oxygen atom of the co‐substrate molecular oxygen may for instance be incorporated into the product while the second atom is reduced to water, again at the expense of one equivalent of NAD(P)H.^[2]^ Consequently, various methods for the regeneration of reduced nicotinamides have been established, using e.g. glucose, alcohols, formate, phosphite or even molecular hydrogen as sacrificial electron donor.[^15^, ^16^] Alternative protocols apply chemical reductants, use light to enable the utilization of cheap donor molecules or directly employ electrons for the regeneration of the cofactor with electrochemical methods.[^15^, ^16^, ^17^, ^18^] In general, many regeneration concepts show a poor atom economy, e.g. from glucose/2‐propanol only two hydrogen atoms are used.

**Figure 1:**
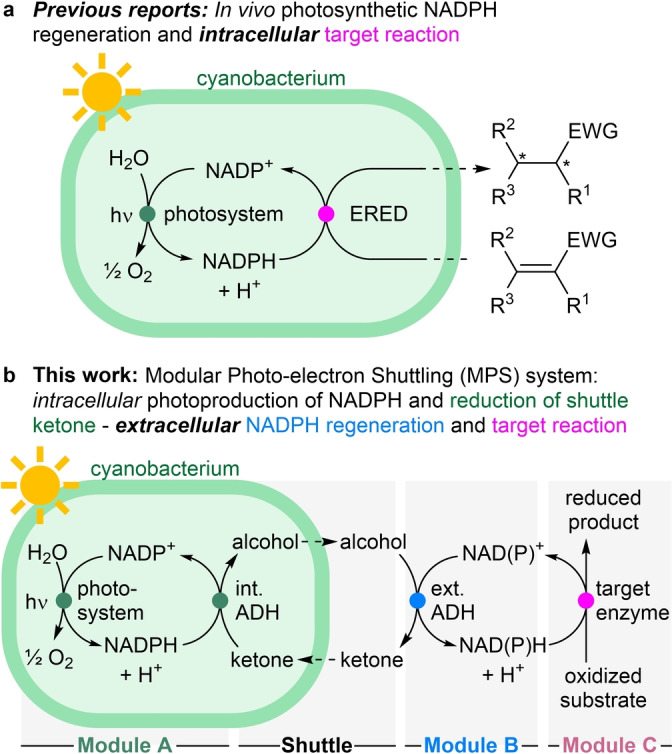
Photosynthetic nicotinamide regeneration using cyanobacteria. a) Photosynthetic water oxidation provides NADPH for an heterologously expressed intracellular oxidoreductase (in the shown example an ene‐reductase, ERED). b) Intracellular photosynthetic NADPH production (Module A) coupled to an extracellular redox reaction (Module C) via electron shuttling. The photosynthetic water oxidation transfers the electrons via an intracellular ADH (Module A) to an alcohol/ketone shuttle (Shuttle) to an extracellular ADH for the regeneration of extracellular NAD(P)H (Module B) which is coupled to the extracellular NAD(P)H‐dependent oxidoreductase (Module C). EWG=electron withdrawing group.

Besides the direct usage of electrons via electrodes for reductions (requiring additional redox mediators)[^18^, ^19^] and maybe the use of molecular hydrogen,^[20]^ arguably one ideal electron donor molecule is water, as it is ubiquitous in biocatalytic reactions and would lead to molecular oxygen as the only co‐product. This has been realized by coupling nature's photosynthesis with an intracellular enzymatic redox reaction (Figure 1a). In the very first report, an NADPH‐dependent ene‐reductase (ERED) was heterologously expressed in *Synechocystis* sp. PCC 6803 (hereafter *Synechocystis*).[^21^, ^22^] The photosynthetic machinery of the cyanobacterial host liberated electrons from water, ultimately producing intracellular NADPH, which in turn was used by the recombinant ERED to perform the biocatalytic reduction of C=C‐bonds. Recently, this approach has been extended to other biocatalysts, including alcohol dehydrogenases (ADHs),[^23^, ^24^] imine reductases (IREDs),^[25]^ monooxygenases,[^26^, ^27^, ^28^] the AlkBGT hydroxylation‐system,^[29]^ and carboxylic acid reductases.^[30]^

However, all of these systems report a range of shortcomings which prevent them from broad application and fast implementation, the most prominent being the challenges associated with cloning and expression of the target enzyme in the applied photoautotrophic organisms.[^31^, ^32^, ^33^] Especially the long period (months) required for the introduction of a target gene in a photoautotrophic host excludes a fast variation and investigation of different expression systems, promotors, constructs, and also a screening of enzyme panels or enzyme variants. Further issues include the problematic heterologous expression of enzymes that are toxic to the cells metabolism or a limited transfer of the substrate or product through the cell‐membrane of the living organism.[^24^, ^25^, ^27^] Finally, the photosynthetic machinery produces NADPH which limits the applicable enzymes to NADPH‐dependent ones.[^21^, ^22^, ^24^] Alternative systems that use isolated thylakoid membranes for cofactor recycling[^34^, ^35^] as well as a whole‐cell system providing formate for extracellular NADH recycling have been reported.^[36]^ Furthermore, light‐dependent regeneration of reduced nicotinamides has also been realized using organic photocatalysts,^[37]^ and heterogeneous photocatalysts in combination with electron mediators.[^38^, ^39^, ^40^, ^41^, ^42^] However, such systems mostly rely on buffer components as electron source.

## Results and Discussion

To overcome selected issues with cyanobacteria, like the challenge of heterologous enzyme expression and others, we designed a system where the electrons photo‐generated by the cyanobacterium are shuttled to the outside of the cell to drive there any redox reaction of interest. This would allow a modular set‐up and a simple exchange of the extracellular reaction (Figure 1b). The modular approach would allow to use one single cyanobacterial strain to provide photosynthetic hydride equivalents in the form of both NADH and NADPH, for any extracellular redox reaction. In brief, in Module A an oxidized small shuttle molecule (e.g. ketone), that is permeable to the cell membrane,^[43]^ is converted to its reduced form (e.g. alcohol) inside the cyanobacterium, thereby taking up one redox equivalent. After passing the cell membrane, the alcohol is re‐oxidized in Module B by an exchangeable external ADH, producing one equivalent of either NADH or NADPH, depending on the ADH‘s cofactor specificity. The reduced nicotinamide is then utilized in the third Module C, encompassing the exchangeable NAD(P)H‐dependent target redox enzyme. Overall, the reaction only requires one equivalent of water and light, and the only side product is molecular oxygen.

To test the idea of modular photo‐electron shuttling (MPS, Figure 1b), wild type *Synechocystis* sp. PCC 6803 (*Synechocystis* wt) was initially chosen for Module A, as it was reported to possess native ADH activity on cyclic ketones.^[26]^ For the back‐translation of the redox equivalents that were generated inside the cyanobacterium and transported through the cell membrane by the shuttle molecule into extracellular NADH, the NADH‐dependent alcohol dehydrogenase (ADH‐A) from *Rhodococcus ruber* DSM 44541[^44^, ^45^, ^46^] was selected as Module B. The ene‐reductase (ERED) OPR3 from *Lycopersicon esculentum*[^47^, ^48^] was chosen as Module C, to reduce the C=C‐bond of 4‐ketoisophorone (**1 a**) as test reaction (Table 1). Although OPR3 was reported to prefer NADPH,^[49]^ it is known to accept NADH as well.^[47]^ From several alcohol/ketone pairs investigated as shuttle molecules (Table S1), cyclohexanol/cyclohexanone was selected for the initial experiment combining all Modules (Modules A+B+C). Although the first experiment led to a conversion of just 4 % (Table 1, entry 1 and Table S2), it indicated that the concept might work, since in the dark no trace of product was formed. The low conversion was attributed to the low native ADH activity in the wild‐type *Synechocystis* sp. PCC 6803. Consequently, the strain was substituted for a recombinant strain of *Synechococcus elongatus* PCC 7942, heterologously expressing the NADPH‐dependent ADH from *Lactobacillus kefir* (*Lk*ADH).[^23^, ^50^] In subsequent experiments the NADPH‐dependent *Lk*ADH was investigated also as catalyst for Module B as NADPH‐providing alternative to the NADH‐recycling ADH‐A. Furthermore, it turned out that acetone/2‐propanol worked even better as shuttle pair (Table S3). Employing now the recombinant *Synechococcus elongatus* (Module A) together with ADH‐A as cell free extract (Module B) allowed to reach 55 % conversion and 66 % using the purified enzyme (Table 1, entries 2–3). Employing the *Lk*ADH (in purified form as well as CFE, with NADP^+^) for NADPH recycling in Module B, allowed to reach quantitative conversion and excellent e.e.s (entries 4–5). Note that this translates to almost 10 turnovers of NADP^+^ and two turnovers of the shuttle molecule for this first preliminary system.

**Table 1:** ]  1 Proof of principle of the modular system exploiting photo‐electron shuttling (MPS) to provide reduced nicotinamide for the reduction of **1 a**.

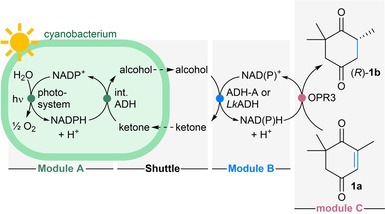								
Entry	Module A	Shuttle	Module B	Module C	Light		Dark	
					conv. [%]	e.e. [%]	conv. [%]	e.e. [%]
1	wt *Syn*.	cyclohexanone (20 mM)	ADH‐A (lyo.), NAD^+^	OPR3	4	n.d.	n.c.	n.d.
2	rec. *S*. *elongatus*	acetone (5 mM)	ADH‐A (purif.), NAD^+^	OPR3	66	97	n.d.	n.d.
3	rec. *S*. *elongatus*	acetone (5 mM)	ADH‐A (CFE), NAD^+^	OPR3	55	96	6	89
4	rec. *S*. *elongatus*	acetone (5 mM)	*Lk*ADH (purif.), NADP^+^	OPR3	>99	96	n.d.	n.d.
5	rec. *S*. *elongatus*	acetone (5 mM)	*Lk*ADH (CFE), NADP^+^	OPR3	>99	95	12	92
6	rec. *S*. *elongatus*	acetone (5 mM)	–	–	32	93	10	92

**Module A**: *Synechocystis* sp. PCC 6803 cells (wt *Syn*.; OD_750_=10) or cells of the recombinant *Lk*ADH‐*Synechococcus elongatus* strain (rec. *S. elongatus*; OD_750_=5). **Module B**: ADH‐A (CFE: 0.5 mg ml^−1^ or purified: 30 μL of a 2.06 mg mL^−1^ stock) and NAD^+^ (1 mM), or *Lk*ADH (CFE: 0.5 mg ml^−1^ or purified: 3 μL of a 24.6 mg mL^−1^ stock) and NADP^+^ (1 mM). **Shuttle**: As indicated. **Module C**: OPR3 (purified enzyme, 100 μg mL^−1^) and **1 a** (10 mM). All in BG11 medium (with 5 mM HEPES/NaOH buffer, pH 8; supplemented with 1 mM MgCl_2_, final volume 1 mL). Reaction overnight (16 h) in a photoreactor with white light (430 μE m^−2^ s^−1^) at room temperature and 600 rpm. Samples in “dark” were covered with aluminum foil. c.=conversion; lyo.=lyophilized whole cells (2 mg mL^−1^); purif.=purified enzyme; CFE=lyophilized cell free extract. n.d.=not determined. n. c.=no conversion. Complete data set is shown in Tables S3 and S4. Product **1 b** is prone to racemization.

**Figure 2:**
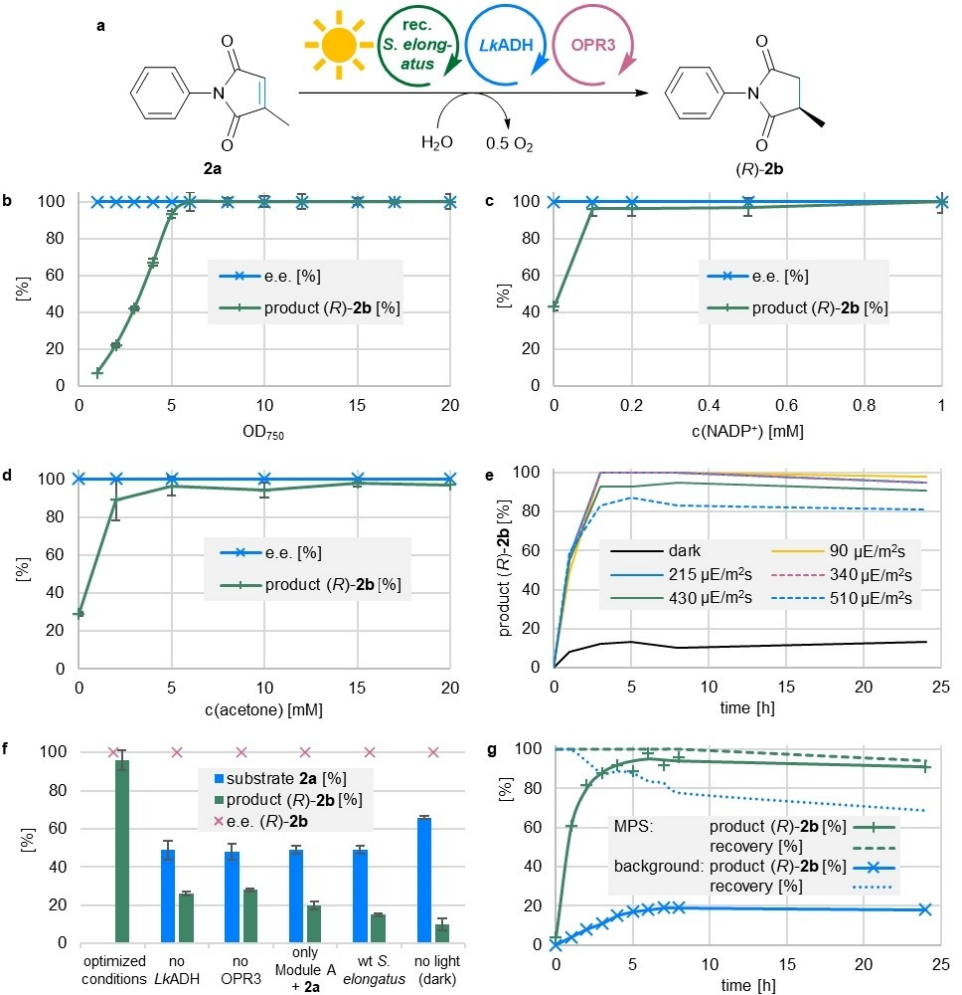
Influence of various parameters on the reduction of **2 a** in the MPS system. a) General reaction scheme for the reduction of **2 a** to **2 b**. b) Loading of recombinant *Synechococcus elongatus* cells (OD_750_=0–20). c) Variation of extracellular NADP^+^ concentration (0–1 mM). d) Loading of the alcohol/ketone shuttle (provided as acetone, 0–20 mM). e) Influence of the illumination conditions (white light, 0–510 μE m^−2^ s^−1^). f) Control reactions, either without external *Lk*ADH, OPR3 or light, background reactivity of the recombinant *Synechococcus elongatus* and performance of the system with the wt *Synechococcus elongatus* (no internal *Lk*ADH). g) Comparison of the MPS with the background reactivity of the wt *Synechococcus elongatus*; total recovery is the sum of recovered product and substrate. **Module A**: Recombinant *Synechococcus elongatus* cells (OD_750_=5, or as indicated), or wt. *Synechococcus elongatus* cells (OD_750_=5). **Module B**: *Lk*ADH (CFE, 0.25 mg mL^−1^, 0.23 U_2‐propanol_ mL^−1^), NADP^+^ (0.1 mM, or as indicated). **Shuttle**: Acetone (5 mM, or as indicated). **Module C**: OPR3 (purified enzyme, 100 μg mL^−1^), DMSO (4 % v/v), and **2 a** (10 mM). All in BG11 medium (with 5 mM HEPES/NaOH buffer, pH 8; supplemented with 1 mM MgCl_2_, final volume 1 mL). Reaction overnight (16 h, or as indicated) in a photoreactor with white light (430 μE m^−2^ s^−1^, or as indicated) at room temperature and 600 rpm. The experiments were performed in two biological replicates, each in triplicate. Error bars represent standard deviation. Samples in “dark” were covered with aluminum foil. c.=concentration.

The higher conversions might be explained by the known preference of OPR3 for NADPH.^[49]^ Control reactions revealed a certain background activity of the cyanobacterium and low reactivity in the dark (Entry 6). This can be attributed to native ERED‐activity in the *Synechococcus elongatus* strain and possibly stored reducing equivalents in the cells.^[51]^ For investigating the influence of essential parameters (cell density, concentrations of NAD(P)^+^ and the shuttle, light intensity and reaction time) the substrate *N*‐phenyl methylmaleimide (**2 a**) was used (Figure 2a), since this substrate is not prone to side reactions such as carbonyl reduction. The substrate **2 a** was supplied to the reaction as a solution in DMSO (for the solvent tolerance of *Synechococcus elongatus* see Figure S2).

Increasing the amount of recombinant *Synechococcus elongatus* cells, measured as OD_750_, from 1 to 5 went in hand with an increase of product formation of (*R*)‐**2 b**, reaching 93 % product formation at an OD_750_ of 5 and quantitative product formation at an OD_750_ of 10 (Figure 2b). As the total recoveries were quantitative at an OD_750_ of 5, this cell loading, corresponding to a dry cell weight of 1.2±0.1 mg mL^−1^ and chlorophyll *a* content of 34±2 μg mL^−1^, was selected for future reactions.

When varying the concentration of the nicotinamide cofactor NADP^+^, the reaction went almost to completion at a NADP^+^ concentration as low as 0.1 mM, which corresponds to 96 turnovers for the cofactor (96 % conversion, Figure 2c). A residual activity that was found when the external addition of NADP^+^ was omitted, which was attributed to the cells native EREDs. A similar amount of product was detected in the absence of acetone due to the same reason. The conversion to the product increased to 89 % when the concentration of acetone was 2 mM (≈4.5 turnovers of acetone; Figure 2d). However, due to the volatility of the shuttle molecule, 5 mM were supplied in the subsequent experiments.

Following the time course of the system under varied illumination conditions revealed that increasing the light intensity to 510 μE m^−2^ s^−1^ photosynthetically active radiation (PAR), reduced the amount of produced **2 b**. In fact, dimming the light intensity improved the system, as under illumination with 215 or 90 μE m^−2^ s^−1^, the reaction was complete within 3 hours (Figure 2e). The apparent specific activity of the overall MPS system [calculated after one hour and estimated by the formation of (*R*)‐**2 b**], increased from 68±10 U g_CDW_^−1^ to 79±10 U g_CDW_^−1^ when the light intensity was amplified from 90 to 215 μE m^−2^ s^−1^ but stayed then almost constant upon further increase of the light intensity. Therefore, an intensity of 215 μE m^−2^ s^−1^ was chosen as optimal for further experiments. These numbers compare well to the specific activities found for a recombinant *Synechocystis* strain expressing the ERED YqjM.^[21]^ In this case, engineering of the NADPH supply in the strain was reported to almost double the specific activity of the recombinant YqjM.^[22]^ Note that the light intensity used during cultivation was 80 μE m^−2^ s^−1^, and adaptation of the cellular photosystem to these conditions is reflected in better performance of the MPS at such lower intensity (90 to 215 μE m^−2^ s^−1^).^[23]^ To put these numbers into context, a PAR of 90 μE m^−2^ s^−1^ corresponds to the light intensity measured on a windowsill behind glass on an autumn day in Graz, Austria. Control reactions were performed omitting one of the integral components of the recycling system (Figure 2f). About 26–28 % of (*R*)‐**2 b** were formed within 16 hours if one of the external enzymes (*Lk*ADH or OPR3) was omitted. When the wild‐type *Synechococcus elongatus* (lacking the recombinant *Lk*ADH) was used for Module A, 15 % of the product (*R*)‐**2 b** were formed and when the recombinant *Synechococcus elongatus* was incubated with **2 a** in the absence of all other components of the recycling system, 20 % of product were found. All these reactivities account for the background ene‐reductase activity of the *Synechococcus elongatus*. When the reaction was performed in the dark, about 10 % conversion to the product were found, originating from redox equivalents stored in the whole *Synechococcus elongatus* cells. The high product formations achieved using the optimized reaction conditions prove the necessity of every single component of the system. In agreement with reports about an ERED originating from *Synechococcus elongatus*,^[51]^ the background reaction exhibited the same stereoselectivity as the target reaction (Figure 2f), therefore the e.e. values of the product were not influenced by the background. For a better understanding of the background reactivity, the time course of the reactions using the MPS was compared with the wild type *Synechococcus elongatus* only (Figure 2g). With the MPS system using OPR3, 61 % of **2 a** were converted to **2 b** within the first hour of the reaction, while only 4 % of **2 a** were converted in the first hour by wild‐type *Synechococcus elongatus*. The slower rate of the background ene‐reduction shows that the contribution of the native ene‐reductases to the overall reaction is only minor when the recycling system is applied. In addition, going in hand with observations made earlier, the recovery of the background reaction was only 69 % after 24 hours, as the slowly transformed substrate could be consumed in side reactions, such as nucleophilic additions of free cysteine groups to the activated electrophilic β‐carbon of the substrate.[^22^, ^52^]

**Figure 3:**
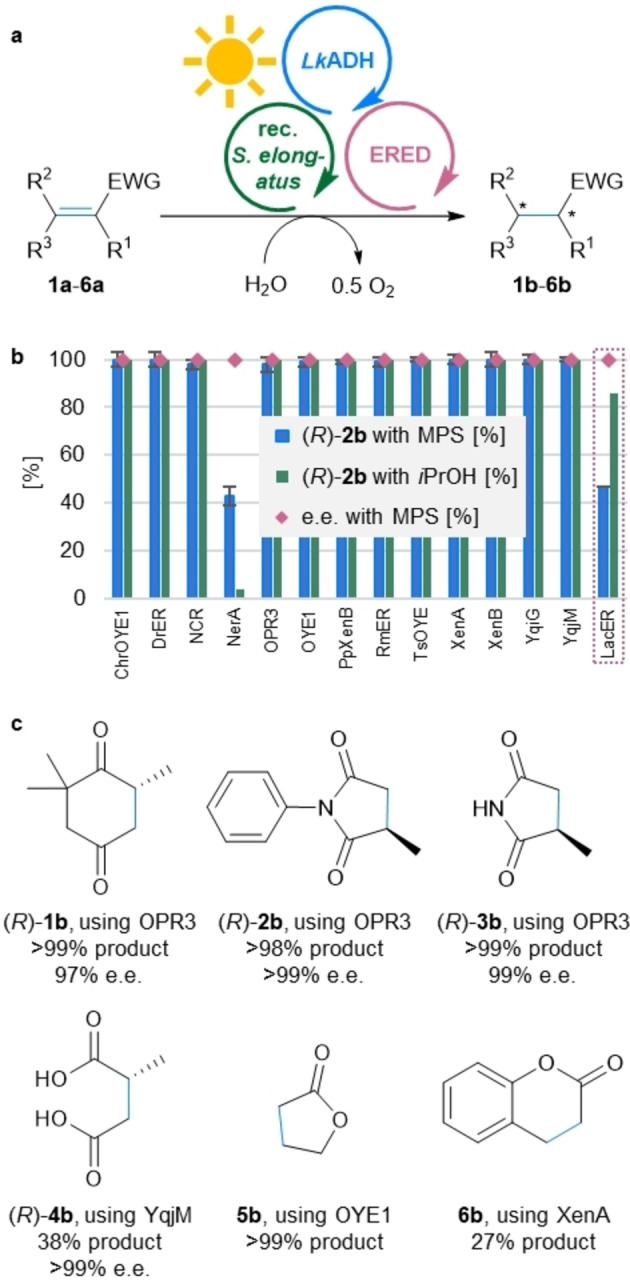
Applicability of the MPS for a library of EREDs and the reduction of various activated alkenes **1 a**–**6 a**. a) General reaction scheme for the reduction of **1 a**–**6 a**. b) Product formation of (*R*)‐**2 b** testing a library of 14 EREDs for the reduction of **2 a** with the MPS or the coupled enzyme regeneration system using LkADH and 2‐propanol (20 mM) as auxiliary substrate (with 2‐PrOH). For LacER, the Module B was exchanged for ADH‐A and NAD^+^. c) Panel of products obtained using various EREDs. **Module A**: Recombinant *Synechococcus elongatus* cells (OD_750_=5). **Module B**: *Lk*ADH (CFE, 0.25 mg mL^−1^, 0.23 U_2‐propanol_ mL^−1^), NADP^+^ (0.1 mM). In case LacER was used in **Module C**: ADH‐A (CFE, 0.25 mg mL^−1^), NAD^+^ (0.1 mM). **Shuttle**: Acetone (5 mM). **Module C**: The indicated ERED (purified enzyme, 100 μg mL^−1^), DMSO (4 % v/v; for substrate **1 a** no DMSO was used) and the indicated substrate **1 a**–**6 a** (10 mM). All in BG11 medium (with 5 mM HEPES/NaOH buffer, pH 8; supplemented with 1 mM MgCl_2_, final volume 1 mL). Reaction overnight (16 h; for substrate **1 a**: 3 h) in a photoreactor with white light (215 μE m^−2^ s^−1^) at 21 °C and 600 rpm. The experiments were performed in at least two biological replicates. Error bars represent standard deviation. EWG=electron withdrawing group. For further data see Tables S6 and S7.

To explore the generality of the optimized MPS, a library of 14 different EREDs was tested in this system using the alkene reduction of **2 a** as model reaction (Figure 3a). As most EREDs are active with both reduced nicotinamide cofactors, NADH and NADPH, *Lk*ADH and NADP^+^ were arbitrary chosen for Module B, except for the case of the more strictly NADH‐dependent *Lac*ER. For 12 out of 14 EREDs the product formation was quantitative or close to quantitative (i.e., 100 turnovers of NADP^+^), demonstrating the broad applicability of the MPS system for ene‐reductases (Figure 3b). Even for NerA from *Rhizobium radiobacter*, giving a product formation of 43 %, the MPS outperformed the (traditional) coupled enzyme regeneration system using *Lk*ADH and 2‐propanol as auxiliary substrate, which gave 4 % of product (experiment performed for comparison, Figure 3b and Table S6). In the case of *Lac*ER, when ADH‐A was applied as Module B to recycle NADH, the traditional cofactor recycling (using ADH‐A and 2‐propanol) gave 86 % product, while MPS resulted in 47 % (*R*)‐**2 b** (i.e., 47 turnovers of NAD^+^; for the complete data set of both regeneration systems see Table S6).

Having shown that the MPS is applicable for a library of EREDs, the system was expanded to the reduction of other activated alkenes (Figure 3c). Using the optimized conditions identified above, the initial test substrate 4‐ketoisophorone (**1 a**) was quantitatively converted to its product (*R*)‐**1 b**. The high e.e. of 97 % is noteworthy, as the chiral centre of the product is prone to racemization.^[53]^ Additionally to the *N*‐phenylated methylmaleimide (**2 a**) also the non‐arylated derivative **3 a** was transformed to the corresponding reduced products with absolute stereoselectivity (99 % e.e.) and >99 % conversion to the product. Employing the ERED YqjM for the reduction of a diacid, which are in general challenging substrates for EREDs, the MPS‐driven reduction of citraconic acid **4 a** led to 38 % of the optically pure (*R*)‐**4 b** (>99 % e.e). The system was further applied to less activated substrates such as the lactone **5 a** and the coumarin **6 a**, resulting in the formation of >99 % of **5 b** and 27 % of the bulkier **6 b**. The reactions were also compared to the substrate coupled recycling (using *Lk*ADH and 2‐propanol) and investigated in a variety of control reactions, e.g. testing the background reactivity of the recombinant *Synechococcus elongatus* cells, the reaction in the dark and in the absence of the ERED (see Table S7). For substrates **4 a** and **6 a**, the substrate coupled recycling gave higher product yields than the MPS (52 % for **4 a** and 63 % for **6 a**). Analogous to substrate **2 a**, the dark controls often gave around 10 % of product due to redox equivalents stored in the cells. In general, the background ERED activity of the cells was either too slow to be competitive or, in case of **4**–**6 a**, was not detectable (Table S7). In case of **1 b**, background reactions due to overreduction by *Lk*ADHs in Module A and Module B were overcome by stopping the reaction after 3 h. MPS reactions with substrate **6 a** showed lower recovery compared to control reactions probably due to product or substrate depletion associated with photobleaching of *Synechococcus elongatus*.

**Figure 4:**
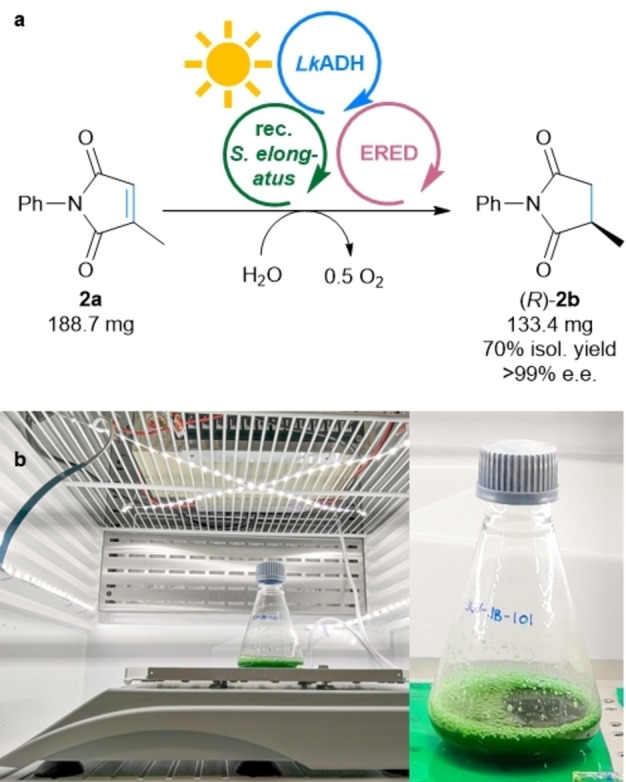
Upscaling of MPS for the reduction of **2 a**. a) General reaction scheme for the reduction of **2 a** to **2 b**. b) Setup of the reaction. **Module A**: Recombinant *Synechococcus elongatus* cells (OD_750_=10) **Module B**: *Lk*ADH (CFE, 0.25 mg mL^−1^, 0.23 U_2‐propanol_ mL^−1^), NADP^+^ (0.1 mM). **Shuttle**: Acetone (20 mM). **Module C**: OPR3 (purified enzyme, 100 μg mL^−1^), no DMSO, and **2 a** (20 mM). All in BG11 medium (with 5 mM HEPES/NaOH buffer, pH 8; supplemented with 1 mM MgCl_2_, final volume 50 mL). Reaction overnight (16 h, or as indicated) in a photoreactor using white light (300 μE m^−2^ s^−1^, or as indicated) at room temperature and 140 rpm.

To be able to run the MPS system for the reduction of **2 a** at a higher substrate concentration than 10 mM, it was tested at 20 mM at varied cell loadings (Table S5). At 20 mM substrate concentration and an OD_750_ of 5 the amount of formed product (*R*)‐**2 b** reached 13.6 mM (68 %). Doubling the cell loading to an OD_750_ of 10 gave 91 % of the product (18.1 mM). Next, the reaction was scaled to 50 mL at a substrate concentration of 20 mM **2 a** (188.7 mg) in a 250 mL Erlenmeyer flask using the ERED OPR3 (Figure 4). After incubation and illumination overnight, 95 % product formation was detected by GC, giving after column chromatography an isolated yield of 70 % enantiopure product (*R*)‐**2 b** (133.4 mg). Motivated by this, the concentration of **2 a** was tested also at 50 mM, which produced up to 46.8±1.1 mM product at an OD_750_ of 25 (for details see Table S5). This translates to ten turnovers of the applied shuttle molecule acetone (5 mM) and 468 turnovers of NADP^+^.

**Figure 5:**
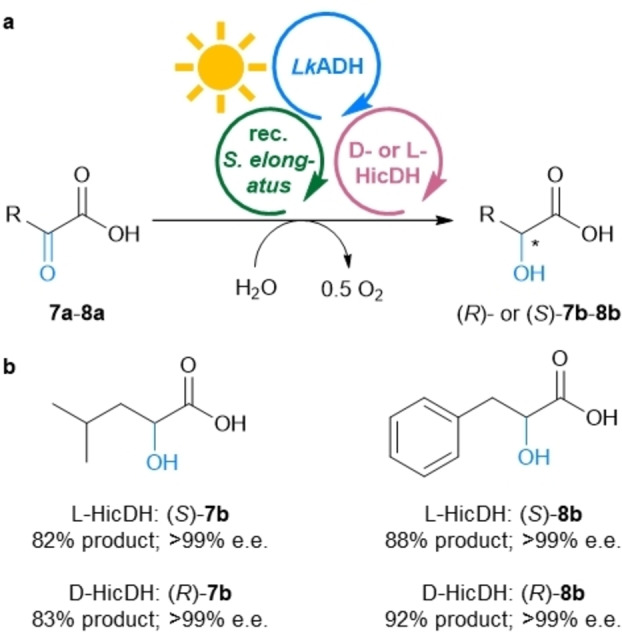
Combination of MPS with keto acid dehydrogenases. a) General reaction scheme for the reduction of **7 a** and **8 a** to both enantiomers of **7 b** and **8 b**. b) Products **7 b** and **8 b**, yields and e.e.s. **Module A**: Recombinant *Synechococcus elongatus* cells (OD_750_=5). **Module B**: *Lk*ADH (CFE, 0.25 mg mL^−1^, 0.23 U_2‐propanol_ mL^−1^), NADP^+^ (0.1 mM). **Shuttle**: Acetone (5 mM). **Module C**: L‐HicDH (CFE, 0.5 mg mL^−1^, 1.8 U_8a_ mL^−1^) or D‐HicDH (CFE, 0.5 mg mL^−1^, 4.7 U_8a_ mL^−1^), and the indicated substrate (10 mM, **7 a** was added as stock solution in BG11, pH adjusted to 7.5; **8 a** was added as stock solution in DMSO, final v/v 4 %) and the corresponding reactions were supplemented with 100 mM HEPES/NaOH pH 8. All in BG11 medium (with 5 mM HEPES/NaOH buffer, pH 8; supplemented with 1 mM MgCl_2_, final volume 1 mL). Reaction overnight (16 h) in a photoreactor with white light (215 μE m^−2^ s^−1^) at room temperature and 600 rpm. The experiment was performed in two biological replicates, each in triplicate.

Since the MPS system worked rather well for the various EREDs, we investigated whether also other classes of NAD(P)H‐dependent enzymes may be used instead of EREDs. For this purpose, the ERED from Module C before was exchanged for two enantiocomplementary keto acid dehydrogenases, namely the L‐HicDH from *Lactobacillus confusus* DSM 20196[^54^, ^55^] and the D‐HicDH from *Lactobacillus paracasei* DSM 20008 (Figure 5).[^55^, ^56^] Using 2‐oxo‐isocaproic acid (**7 a**) as substrate allowed to reach the corresponding hydroxy acid (*S*)‐**7 b** as well as the enantiomer (*R*)‐**7 b** with 82–83 % product formation each in optically pure form (>99 % e.e.; up to 83 turnovers of NADP^+^) (Figure 5, Table S8). The aromatic substrate phenylpyruvate (**8 a**) led to even higher product formation, reaching 88 % for the hydroxy acid (*S*)‐**8 b** and 92 % for the product (*R*)‐**8 b** (i.e., up to 92 turnovers of NADP^+^). As both enantiocomplementary enzymes exhibit excellent stereoselectivities, both enantiomers of the products could be accessed with an e.e. of >99 %. Comparing the results obtained here with a classic enzyme coupled recycling system shows that the MPS leads to comparable amounts of product formation (Table S8).

In a recent report, the two HicDHs were recombinantly expressed in a modified *Synechocystis* strain and applied for the biocatalytic reduction of α‐keto acids.^[24]^ But due to low expression levels of the HicDHs in the *Synechocystis* strain, and limited substrate transport through the cell membrane, this system required high cell loadings (OD_750_ of 20) and suffered from low reaction rates. Additionally, the whole‐cell catalysed reaction was demonstrated to be independent from light. In contrast, using the here presented MPS, substrate/product transport is no problem as the reaction happens outside the whole cell [additionally here the background reaction using only the recombinant *Synechococcus elongatus* cells or the dark reactions showed only low reactivity (Table S8)].

**Figure 6:**
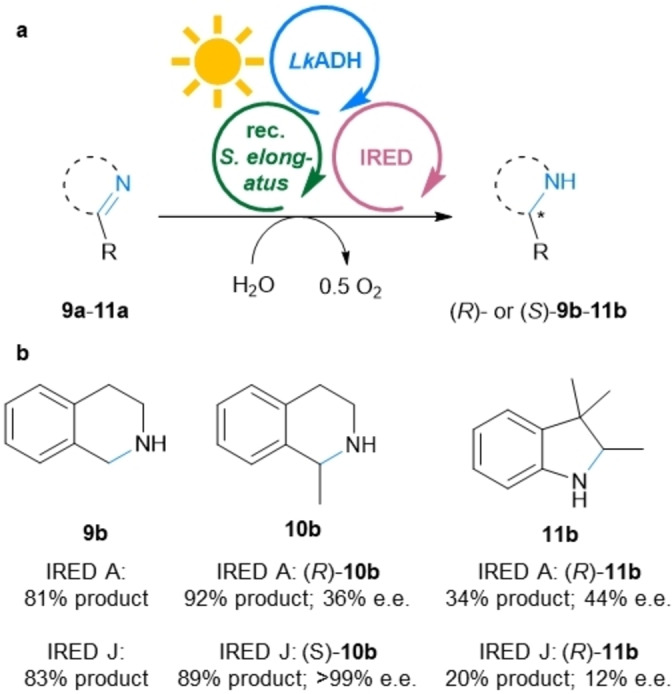
MPS combined with IREDs. a) General reaction scheme for the reduction of **9 a**–**11 a**. b) Products **9 b**–**11 b**, yields and e.e.s. **Module A**: Recombinant *Synechococcus elongatus* cells (OD_750_=5). **Module B**: *Lk*ADH (CFE, 0.25 mg mL^−1^, 0.23 U_2‐propanol_ mL^−1^), NADP^+^ (0.1 mM). **Shuttle**: Acetone (5 mM). **Module C**: The indicated IRED (CFE, 4 mg mL^−1^; IRED A, 0.4 U_9a_ mL^−1^ IRED J 1.16 U_9a_ mL^−1^), DMSO (5 % v/v), and the indicated substrate **9 a**, **10 a** or **11 a** (10 mM). All in BG11 medium (with 5 mM HEPES/NaOH buffer, pH 8; supplemented with 1 mM MgCl_2_, final volume 1 mL). Reaction overnight (16 h) in a photoreactor with white light (215 μE m^−2^ s^−1^) at room temperature and 600 rpm. The experiment was performed in two biological replicates, each in triplicate.

Similar to the HicDHs, IREDs have already been recombinantly expressed in *Synechocystis* sp. PCC 6803,^[25]^ and although this whole cell catalyst allowed to convert up to 8 mM of imine substrate, the reduction reaction was not light‐dependent and relied on redox equivalents stored in the cells. Therefore, the reduction of substrates **9 a**–**11 a** was reinvestigated using the presented MPS system with the IRED A from *Streptomyces* sp. and IRED J from *Kribbella flavida* DSM 17836.[^25^, ^57^] The MPS system allowed to reach a light dependent product formation for substrates **9 a**–**11 a** of up to 92 % (92 turnovers of NADP^+^, Figure 6).

Again the results were mostly comparable with the results of the classic cofactor regeneration reactions reported in vitro (see also Table S9).^[57]^ The employed IREDs gave the stereocomplementary products for the reduction of **10 a** with an e.e. of 36 % for (*R*)‐**10 b** (IRED A) and >99 % for (*S*)‐**10 b** (IRED J). Interestingly, the formation of (*R*)‐**11 b**, using either enzyme, was limited to about 50 % of the conversion that was obtained with the coupled enzyme recycling system (only Modules B+C, supplying an excess of 2‐propanol; IRED A: 70 % conversion; IRED J: 43 % conversion; Table S9). For all these imine substrates, very low background activity in the dark and no background imine‐reductase activity of the *Synechococcus elongatus* were detected. Furthermore, it is worth to mention that imine substrates are reported to be toxic for cyanobacterial cells and therefore, higher cell loadings were required for the in vivo system.^[25]^ Herein, such toxicity was not an issue, possibly due to the enhanced reaction rates, leading to a fast decrease of the concentration of the potentially harmful substrate.

**Figure 7:**
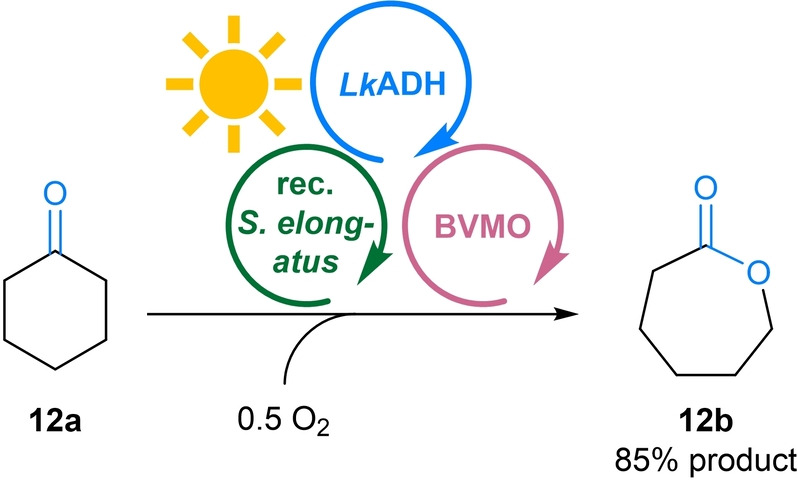
MPS combined with monooxygenations. General reaction scheme for the Baeyer–Villiger monooxygenation of **12 a** to **12 b**. **Module A**: Recombinant *Synechococcus elongatus* cells (OD_750_=5). **Module B**: *Lk*ADH (CFE, 0.25 mg mL^−1^, 0.23 U_2‐propanol_ mL^−1^), NADP^+^ (0.1 mM). **Shuttle**: No shuttle molecule added as the intermediary **12 c** acts as shuttle. **Module C**: CHMO (CFE, 2 mg mL^−1^, 0.22 U_12a_ mL^−1^), and **12 a** (10 mM). All in BG11 medium (with 5 mM HEPES/NaOH buffer, pH 8; supplemented with 1 mM MgCl_2_, final volume 1 mL). Reaction overnight (16 h) in a photoreactor with white light (215 μE m^−2^ s^−1^) at room temperature and 600 rpm. The experiment was performed in two biological replicates, each in triplicate.

Motivated by these results we went forward to use the MPS to fuel a Baeyer–Villiger oxidation catalysed by a monooxygenase (BVMO) with the required reduced nicotinamide cofactor. For this reaction using the MPS, only the substrate (cyclohexanone **12 a**) and oxygen are required as reagents (Figure 7, for all details see Figure S3). All other components of the system are only present in catalytic amounts. In detail, besides the substrate, the monooxygenase requires one molecule of oxygen and NADPH to produce the product and one molecule of water. The water is utilized by the cyanobacterium to generate the required NADPH and half an equivalent of oxygen.

When the MPS/BVMO oxidation of **12 a** (10 mM) was performed using acetone as shuttle as described above, 84 % of ϵ‐caprolactone (**12 b**) as well as a small amount of cyclohexanol (**12 c**) were formed within 16 h. Incubation of substrate **12 a** only with the recombinant *Synechococcus elongatus* under illumination, thus in the absence of Modules B and C, resulted exclusively in the formation of the alcohol **12 c**. The ketone reduction can be attributed to the activity of the recombinant *Lk*ADH in the cyanobacterium (compare Table S10 and Table S3). As this suggests that the substrate **12 a** as well as the alcohol **12 c** easily surpasses the cell membrane, it was envisioned that cyclohexanone/cyclohexanol may act as shuttle, thus acetone may be omitted. Indeed, the reaction in the absence of acetone as shuttle led to the formation of 85 % of **12 b** (Figure 7). Following the reaction over time revealed that in a first phase the substrate **12 a** is reduced to the alcohol **12 c** which then is further converted to the product **12 b** via oxidation (and thereby recycling the cofactor and substrate) and monooxygenation (Figure S3 and Figure S4). Note that the reduction of **12 a** to **12 c** has already been reported when heterologous BVMOs, expressed in *Synechocystis* sp. PCC 6803 were challenged with **12 a**, in this case leading to a loss of product.^[26]^ The problem was later overcome by using a faster BVMO.^[28]^

## Conclusion

In conclusion, many previously noted limitations of cyanobacteria used for cofactor regeneration can be overcome using MPS. For instance, the modularity allowed the extracellular regeneration of both reduced nicotinamide cofactors, NADH and NADPH, while cyanobacteria provide mainly intracellular NADPH. In nature transmembrane electron shuttling is described e.g. for the malate‐aspartate shuttle or the glycerol‐3‐phosphate shuttle,[^58^, ^59^] thus the here presented concept represent a new‐to‐nature approach. Furthermore, the modular design allowed the fast evaluation of a panel of 14 different EREDs, without the requirement for the time‐consuming cloning of each individual enzyme into a cyanobacterial host. Additionally, key parameters, such as the concentration of the involved enzymes or the amount of cyanobacterial cells can easily be adapted, as demonstrated in the semi‐preparative ERED‐catalysed reduction of **2 a** to (*R*)‐**2 b**, reaching substrate concentrations of up to 50 mM and a volume of 50 mL. The approach also minimizes the risk of toxic effects of the heterologous enzyme on the host cells metabolism, as was previously reported for heterologous HicDHs in cyanobacteria.^[24]^ Applying HicDHs with the presented system, allowed not only to overcome this toxicity but also to circumvent the reported problem of limited substrate uptake through the cell membrane.^[24]^ The reaction rates and product formations increased as compared to previous approaches, which also allowed to handle toxic substrates such as imines.^[25]^

However, the application of toxic substrates at higher concentration may be limited depending on the rate at which they are transformed to less harmful products. A further limitation of the MPS represents the required ADHs, which might also react with substrates that contain a carbonyl function, as experienced for substates **1 a** and **12 a**. When using EREDs a slow background activity was detected, which might turn out to be challenging in case the cyano‐enzyme display a different stereoselectivity than the enzymes applied in Module C. Furthermore, elongated reaction times with the ERED substrates resulted in reduced recoveries due to side reactions. A further challenge might be the application of the system at larger scale. While an upscaling to 50 mL was straight‐forward, even larger scales will have to address issues with self‐shading of the cells and reduced light penetration.

Strategies to improve the performance of the presented systems include the testing of lower concentrations of NAD(P)^+^ and optimizing the growth conditions of the cyanobacterium to accelerate photosynthesis. For example, the illumination conditions during growth are reported to have a significant influence on the photosynthetic activity.^[23]^ Furthermore, engineering of the cyanobacterium itself, e.g., its NADPH utilization^[22]^ or of the promotors to tune the heterologous expression levels,^[24]^ have already been shown to be beneficial in related systems.

The performance of the presented approach compared well with the established coupled enzyme regeneration system, using e.g. 2‐propanol for cofactor recycling, but has the advantage just to require light and water. The modular photo‐electron shuttling method was successfully demonstrated for 19 enzymes of four classes of redox reactions and for twelve different substrates. The modular photo‐electron shuttling (MPS) system presented herein leverages the photosynthesis‐driven cofactor recycling concepts based on cyanobacteria[^21^, ^22^, ^23^, ^24^, ^25^, ^26^, ^27^, ^28^, ^29^, ^30^] to a new level and may open new avenues for light dependent biocatalytic redox reactions.

## Conflict of interest

Valentina Jurkaš, Florian Weissensteiner, Sarah Bierbaumer, Christoph K. Winkler and Wolfgang Kroutil are authors of a patent application: Austrian priority application, filed 18/03/2021. A55/2021

1

## Supporting information

As a service to our authors and readers, this journal provides supporting information supplied by the authors. Such materials are peer reviewed and may be re‐organized for online delivery, but are not copy‐edited or typeset. Technical support issues arising from supporting information (other than missing files) should be addressed to the authors.

Supporting Information

## Data Availability

All methods and the data that support the findings of this study are available in the supplementary material of this article.
