# Supplementary material for: Transmembrane Shuttling of Photosynthetically Produced Electrons to Propel Extracellular Biocatalytic Redox Reactions in a Modular Fashion
Source: Angew Chem Weinheim Bergstr Ger. 2022 Aug 29;134(40):e202207971. doi: 10.1002/ange.202207971 (PMC10946770; doi:10.1002/ange.202207971)
Supplement: Supplementary file 1 — Supporting Information [file ANGE-134-0-s001.pdf]

Angewandte Chemie
Eine Zeitschrift der Gesellschaft Deutscher Chemiker

Supporting Information

Transmembrane Shuttling of Photosynthetically Produced Electrons to Propel Extracellular Biocatalytic Redox Reactions in a Modular Fashion

V. Jurkaš, F. Weissensteiner, P. De Santis, S. Vrabl, F. A. Sorgenfrei, S. Bierbaumer, S. Kara, R. Kourist, P. P. Wangikar, C. K. Winkler, W. Kroutil**

1 Table of Contents

1 Table of Contents 2

2 Supplementary Data 4

2.1 Initial test of alcohol/ketone shuttle pairs for Module A and the combined Modules B+C ... 4

2.2 Modular photo-electron shuttling (Modules A+B+C) using cyclohexanol/cyclohexanone and derivatives as alcohol/ketone shuttle pairs 4

2.3 Test of different alcohol/ketone shuttle pairs in the combined Modules B+C with *Lk*ADH ... 5

2.4 Optimization of Module A with the recombinant *Synechococcus elongatus* strain together and Module B using either ADH-A or *Lk*ADH 6

2.5 Optimization of the amount of ADH-A supplied to Module B+C and the combined Modules A+B+C 7

2.6 Tolerance of *Synechococcus elongatus* to DMSO and DMF 7

2.7 Scaling up the substrate loading 8

2.8 Application of the MPS system to a library of ene-reductases 9

2.9 Substrate scope of ene-reductases driven by the MPS recycling system 10

2.10 Application of the MPS for keto acid dehydrogenases 11

2.11 Application of the MPS for imine reductases 12

2.12 Application of the MPS for a Baeyer Villiger monooxygenase (CHMO) 13

3 Supplementary Methods 15

3.1 General, kits, instruments and chemicals 15

3.2 Synthesis of *rac*-2b 15

3.3 Source of organisms 15

3.4 Enyzmes 15

3.5 Expression of enzymes in *E. coli* 16

3.5.1 Transformation 16

3.5.2 Cultivation 16

3.5.3 Harvesting 16

3.5.4 Preparation of cell-free extracts 17

3.5.5 SDS-PAGE 17

3.6 Preparation of purified enzymes 19

3.6.1 Cell lysis 19
3.6.2 Strep-tag purification 19
3.6.3 His-tag purification 19
3.6.4 Storage 19
3.7 Cultivation of cyanobacteria 20
3.8 Determination of the cell dry weight and chlorophyll *a* content 20
3.8.1 Cell dry weight 20
3.8.2 Chlorophyll *a* 21
3.9 General procedure for the fully assembled cofactor recycling system (Modules A+B+C) 21
3.10 50 mL scale reduction of 2a, utilizing the MPS. 22
4 Analytics and Chromatographic Data 23
4.1 NMR 27
4.2 Representative chromatograms 28
4.3 Calibration curves 46
5 References 49

2 Supplementary Data

2.1 Initial test of alcohol/ketone shuttle pairs for Module A and the combined Modules B+C

Table S1. Initial test of alcohol/ketone shuttle pairs for Module A and the combined Modules B+C with ADH-A and the ene-reductase OPR3 for different alcohol/ketone shuttle pairs.

Alcohol/Ketone Shuttle Pair	**Module A** conversion of shuttle ketone to alcohol [%]	**Modules B+C** conversion of **1a** to **1b** [%]
phenylethanol/acetophenone	n.c.	37
2-chlorophenylethanol/2-chloroacetophenone	5	n.d.
1-phenyl-2-propanol/phenylacetone	n.c.	22
6-methyl-5-hepten-2-ol/6-methyl-5-heptene-2-one	n.c.	41
2-octanol/2-octanone	n.c.	46
cyclohexanol/cyclohexanone	**9**	**11**
2-methylcyclohexanol/2-methylcyclohexanone	21	n.d.
4-methylcyclohexanol/4-methylcyclohexanone	7	n.d.

Module A: *Synechocystis* sp. PCC 6803 cells (OD_{750} = 10) and the indicated ketone-shuttle (20 mM ketone) in BG11 medium (with 5 mM HEPES/NaOH buffer, pH 8, final volume 1 mL) overnight in a photoreactor with white light (430 µE m^{-2} s^{-1}) at 30 °C and 600 rpm. **Modules B+C**: ADH-A (lyophilized *E. coli* cells, 2 mg mL^{-1}), NAD^+ (1 mM), OPR3 (purified enzyme, 100 µg mL^{-1}), the indicated alcohol shuttle (20 mM) and **1a** (10 mM) in BG11 medium (with 5 mM HEPES/NaOH buffer, pH 8, final volume 1 mL) overnight in a photoreactor with white light (430 µE m^{-2} s^{-1}) at 30 °C and 600 rpm. n.d. = not determined. n.c. = no conversion detected.

2.2 Modular photo-electron shuttling (Modules A+B+C) using cyclohexanol/cyclohexanone and derivatives as alcohol/ketone shuttle pairs

Table S2. MPS (Modules A+B+C) using cyclohexanol/cyclohexanone derivatives as alcohol/ketone shuttle pairs, with *Synechocystis* sp. PCC 6803 cells (Module A), ADH-A (Module B) and the ene-reductase OPR3 (Module C) with **1a** as substrate.

Alcohol/Ketone Shuttle Pair	Illumination	Conversion of Shuttle Ketone to Alcohol [%]	Conversion of 1a to 1b [%]
cyclohexanol/ cyclohexanone	light	6	**4**
	dark	3	n.c.
2-methylcyclohexanol/ 2-methylcyclohexanone	light	11	1
	dark	5	n.c.
4-methylcyclohexanol/ 4-methylcyclohexanone	light	2	2
	dark	1	2

Synechocystis sp. PCC 6803 cells (OD_{750} = 10), ADH-A (lyophilized whole cells, 2 mg mL^{-1}), the indicated ketone-shuttle (20 mM), NAD^+ (1 mM), OPR3 (purified enzyme, 100 µg mL^{-1}) and **1a** (10 mM) in BG11 medium (with 5 mM HEPES/NaOH buffer, pH 8, final volume 1 mL) overnight in a photoreactor with white light (430 µE m^{-2} s^{-1}) at 30 °C and 600 rpm. Samples in “dark” were covered with aluminum foil. n.c. = no conversion detected.

2.3 Test of different alcohol/ketone shuttle pairs in the combined Modules B+C with *Lk*ADH

Table S3. Test of different alcohol/ketone shuttle pairs in the combined Module B with *Lk*ADH, and Module C with the ene-reductase OPR3 and **1a** as substrate.

**Alcohol/Ketone Shuttle Pair**	**Recycling Module B**	
	conversion of shuttle alcohol to ketone [%]	conversion of **1a** to **1b** [%]
cyclohexanol/cyclohexanone	17	82
(*R*)-phenylethanol/acetophenone	78	>99
2-propanol/acetone	**n.d.**	**>99**

*Lk*ADH (purified enzyme, 73.8 µg mL^{-1}), shuttle provided as alcohol (20 mM), $NADP^+$ (1 mM), OPR3 (purified enzyme, 100 µg mL^{-1}) and **1a** (10 mM) in BG11 medium (with 5 mM HEPES/NaOH buffer, pH 8; supplemented with 1 mM $MgCl_2$, final volume 1 mL) overnight in a photoreactor with white light (430 µE m^{-2} s^{-1}) at 30 °C and 600 rpm. n.d. = not determined.

2.4 Optimization of Module A with the recombinant *Synechococcus elongatus* strain together and Module B using either ADH-A or *Lk*ADH

Table S4. Optimization of Module A with the recombinant *Synechococcus elongatus* strain together with Module B, using ADH-A or *Lk*ADH and Module C with the ene-reductase OPR3 and **1a** as substrate.

**Illumination**	**Module A** recombinant *Synechococcus elongatus* (OD_{750})	**Module B** enzyme	form	amount	**(*R*)-1b** c. [mM]	e.e. [%]	**Total Recovery** [%]
light	5	*Lk*ADH	CFE	0.25 mg	8.9	96	89
	5			0.5 mg	>9.9	95	>99
	10			0.25 mg	2.4	95	24
	10			0.5 mg	0.6	>99	6
light	5	*Lk*ADH	purified	3 µL	>9.9	96	>99
	5			6 µL	>9.9	96	>99
	10			3 µL	7.1	96	71
	10			6 µL	5.4	96	54
light	5	ADH-A	CFE	0.25 mg	5.9	97	95
	5			0.5 mg	5.5	96	95
	10			0.25 mg	6.5	97	77
	10			0.5 mg	6.1	96	75
light	5	ADH-A	purified	15 µL	4.6	97	96
	5			30 µL	6.6	97	97
	10			15 µL	5.8	97	84
	10			30 µL	7	96	84
light	5[a]	*Lk*ADH	CFE	0.25 mg	2.3	97	>99
	5[a]	ADH-A	CFE	0.25 mg	2.2	97	>99
dark	5	*Lk*ADH	CFE	0.5 mg	1.2	92	>99
	5	ADH-A	CFE	0.5 mg	0.6	89	>99
light	5	no ADH added			3.2	93	>99
dark	5	no ADH added			1	92	>99

Combined Modules A+B+C. **Module A**: Cells of the recombinant *Synechococcus elongatus* strain expressing *Lk*ADH. **Module B**: Either ADH-A (CFE or purified from a 2.06 mg mL^{-1} stock) and NAD^+ (1 mM), or *Lk*ADH (CFE or purified from a 24.6 mg mL^{-1} stock) and $NADP^+$ (1 mM). **Shuttle**: Acetone (5 mM). **Module C**: OPR3 (purified enzyme, 100 µg mL^{-1}) and **1a** (10 mM) in BG11 medium (with 5 mM HEPES/NaOH buffer, pH 8; supplemented with 1 mM $MgCl_2$, final volume 1 mL). Reaction overnight (16 h) in a photoreactor with white light (430 µE m^{-2} s^{-1}) at room temperature and 600 rpm. Samples in "dark" were covered with aluminum foil. c. = concentration; purified = purified enzyme; CFE = lyophilized cell free extract. Total recovery is the sum of recovered product and substrate.

2.5 Optimization of the amount of ADH-A supplied to Module B+C and the combined Modules A+B+C

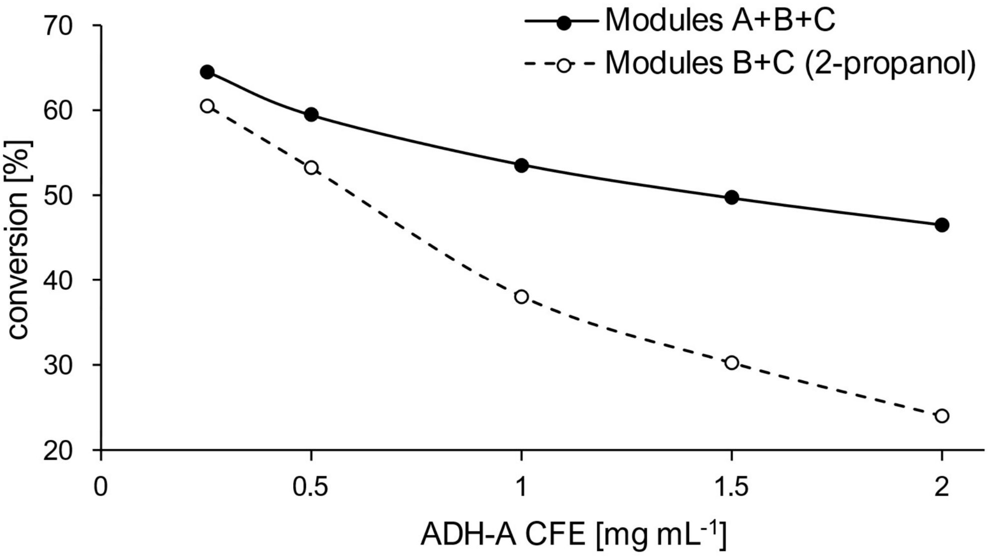

Figure S1. Optimization of the amount of ADH-A supplied to Module B+C and the combined Modules A+B+C. Combination of Modules A+B+C: **Module A**: Cells of the recombinant *Synechococcus elongatus* strain expressing *Lk*ADH (OD_{750} = 7). **Module B**: ADH-A (CFE, 0.5 - 2 mg mL^{-1} as indicated) and NAD^+ (1 mM), and acetone (10 mM) as shuttle. **Module C**: OPR3 (purified enzyme, 100 µg mL^{-1}) and **1a** (10 mM) in BG11 medium (with 5 mM HEPES/NaOH buffer, pH 8; final volume 1 mL). **Combination of Modules B+C (coupled enzyme recycling)**: 2-propanol (20 mM) was supplied instead of acetone in Module B. Reaction overnight (16 h) in a photoreactor with white light (430 µE m^{-2} s^{-1}) at room temperature and 600 rpm.

2.6 Tolerance of *Synechococcus elongatus* to DMSO and DMF

At up to 10% DMSO and 5% DMF no color change was observed. At 20% DMSO and 10% DMF, slight color change to turquoise could be observed. DMSO was chosen as the preferred co-solvent as it is generally considered safer and could be added in higher volume if necessary.

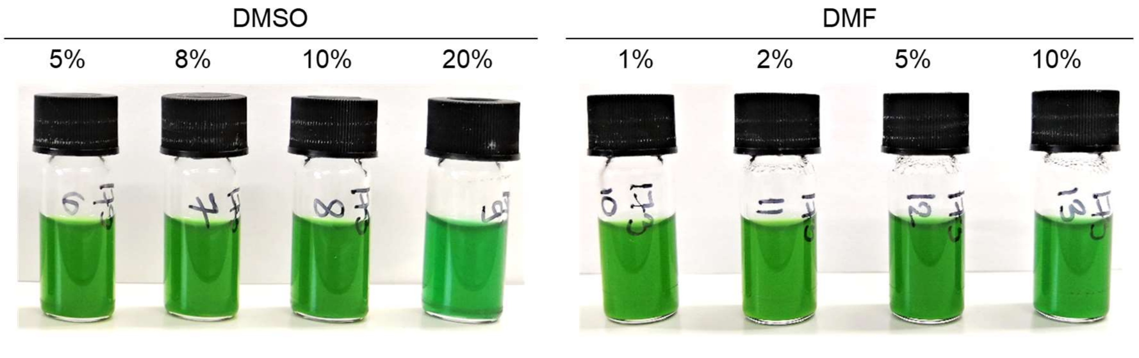

Figure S2. Color of the recombinant *Synechococcus elongatus* cells after incubation with different concentrations of co-solvents DMSO and DMF in BG11. *Synechococcus elongatus* strain expressing *Lk*ADH (OD_{750} = 5), Reaction overnight (16 h) in a photoreactor with white light (430 µE m^{-2} s^{-1}) at room temperature and 600 rpm.

2.7 Scaling up the substrate loading

Table S5. Running the fully assembled system with OPR3 at increased concentrations of **2a**.

**Substrate** c. [mM]	**Module A** *recombinant Synechococcus elongatus* (OD_{750})	**Reaction Conditions**	**2a** c. [mM]	**(*R*)-2b** c. [mM]	e.e. [%]	**Total Recovery** [%]
20	5	light	3.1 ± 0.2	13.6 ± 0.5	>99	83 ± 2
	10	light	0.1 ± 0.1	18.1 ± 0.3	>99	91 ± 1
		dark	15.0 ± 0.1	3.0 ± 0.0	>99	90 ± 0
		cells only	9.7 ± 0.2	7.1 ± 0.0	>99	84 ± 1
50	5	light	34.8 ± 0.6	12.7 ± 0.4	>99	95 ± 1
	10	light	17.7 ± 1.1	29.0 ± 0.7	>99	93 ± 1
	25	light	1.2 ± 0.7	46.8 ± 1.1	>99	96 ± 3
		dark	42.5 ± 0.8	6.8 ± 0.1	>99	99 ± 2
		cells only	29.5 ± 0.8	17.6 ± 0.3	>99	94 ± 2

Module A: Cells of the recombinant *Synechococcus elongatus* strain expressing *Lk*ADH (OD_{750} as indicated). **Module B**: *Lk*ADH (CFE, 0.25 mg mL^{-1}, 0.23 $U_{2\text{-propanol}}$ mL^{-1}) and $NADP^+$ (0.1 mM). **Shuttle**: Acetone (5 mM). **Module C**: OPR3 (purified enzyme, 100 µg mL^{-1}) and **2a** (20-50 mM, as indicated) in BG11 medium (with 5 mM HEPES/NaOH buffer, pH 8; supplemented with 1 mM $MgCl_2$, final volume 1 mL). Reaction overnight (16 h) in a photoreactor with white light (215 µE m^{-2} s^{-1}) at room temperature and 600 rpm. **Light**: combined Modules A+B+C; **Dark**: combined Modules A+B+C with the vials covered with aluminum foil. **Cells only**: Background reactivity of illuminated recombinant *Synechococcus elongatus* with **2a**, only. Average and standard deviation of two independent experiments, each as technical triplicates. c. = concentration; CFE = lyophilized cell free extract.

2.8 Application of the MPS system to a library of ene-reductases

Table S6. Biocatalytic reduction of **2a** with different ene-reductases, comparing the fully assembled regeneration system with the ADH-based coupled enzyme regeneration system (Modules B+C).

	**Modules B+C (20 mM 2-propanol)**				**Modules A+B+C**			
	**2a**	(*R*)-**2b**		**total recovery**	**2a**	(*R*)-**2b**		**total recovery**
**ene-reductase**	c. [mM]	c. [mM]	e.e. [%]	[%]	c. [mM]	c. [mM]	e.e. [%]	[%]
*Chr*OYE1	<0.2	>9.9	>99	>99	<0.2	>9.9 ± 0.3	>99	>99
*Dr*ER	<0.2	>9.9	>99	>99	<0.2	>9.9 ± 0.3	>99	>99
NCR	0.2	>9.9	>99	>99	<0.2	9.8 ± 0.2	>99	98 ± 2
NerA	7.1	0.4	>99	75	3.4 ± 0.5	4.3 ± 0.4	>99	77 ± 2
OPR3	<0.2	>9.9	>99	>99	<0.2	9.8 ± 0.3	>99	99 ± 3
OYE1	0.3	>9.9	>99	>99	n.d.	9.9 ± 0.2	>99	99 ± 2
*Pp*XenB	<0.2	9.9	>99	>99	<0.2	9.9 ± 0.1	>99	>99
*Rm*ER	<0.2	>9.9	>99	>99	<0.2	9.9 ± 0.2	>99	99 ± 2
*Ts*OYE	n.d.	>9.9	>99	>99	<0.2	>9.9 ± 0.1	>99	>99
XenA	0.2	>9.9	>99	>99	<0.2	>9.9 ± 0.2	>99	>99
XenB	0.2	>9.9	>99	>99	<0.2	>9.9 ± 0.3	>99	>99
YqiG	0.2	>9.9	>99	>99	<0.2	>9.9 ± 0.2	>99	>99
YqjM	0.3	>9.9	>99	>99	<0.2	>9.9 ± 0.1	>99	>99
*Lac*ER[a]	1.1	8.6	>99	96	3.4	4.7	>99	81
*Lac*ER[b]	<0.2	1.6	>99	16	-	-	-	-

Combination of Modules A+B+C : **Module A**: Recombinant *Synechococcus elongatus* cells (OD_{750} = 5). **Module B**: *Lk*ADH (CFE, 0.25 mg mL^{-1}, 0.23 $U_{2\text{-propanol}}$ mL^{-1}), $NADP^+$ (0.1 mM). **Shuttle**: Acetone (5 mM). **Module C**: The indicated ERED (purified enzyme, 100 µg mL^{-1}), DMSO (4% v/v), **2a** (10 mM). All in BG11 medium (with 5 mM HEPES/NaOH buffer, pH 8; supplemented with 1 mM $MgCl_2$, final volume 1 mL). **Combination of Modules B+C (coupled enzyme recycling)**: 2-propanol (20 mM) was supplied instead of acetone in Module B. Reaction overnight (16 h) in a photoreactor with white light (215 µE m^{-2} s^{-1}) at room temperature and 600 rpm. The experiment was performed as a single measurement or in biological triplicates (average and standard deviation given). c. = concentration; [a]NADH recycling: ADH-A (CFE, 0.25 mg mL^{-1}, 0.02 $U_{2\text{-propanol}}$ mL^{-1}) instead of *Lk*ADH, NAD^+ (0.1 mM) instead of $NADP^+$, no $MgCl_2$. [b]without Module A (no recycling system), 10 mM NADH.

2.9 Substrate scope of ene-reductases driven by the MPS recycling system

Table S7. Substrate scope of ene-reductases driven by the MPS (Modules A+B+C), control reactions and comparison to an ADH-based coupled enzyme recycling system (Modules B+C).

Substrate	Ene-Reductase	Light	Modules	Substrate c. [mM]	Product c. [mM]	Product ee [%]	Total Recovery [%]
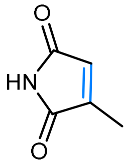 **3a**	OPR3	+	A+B+C	n.d.	10.2 ± 0.3	(*R*) >99	>99
		-	A+B+C	7.4 ± 0.3	1.2 ± 0.1	(*R*) >99	86 ± 2
		-	B+C[a]	n.d.	10.4	(*R*) >99	>99
	-	+	A	5.0 ± 0.4	4.0 ± 0.2	(*R*) >99	90 ± 2
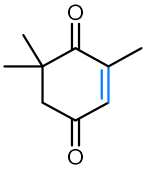 **1a**[a]	OPR3	+	A+B+C	<0.2	9.9 ± 0.3	(*R*) 97 ± 1	>99
		-	A+B+C	9.5 ± 0.2	0.6 ± 0.2	(*R*) 96 ± 4	>99
		-	B+C[a]	n.d.	10.2	(*R*) 98	>99
	-	+	A	9.3 ± 0.1	0.8 ± 0.1	(*R*) 98 ± 2	>99
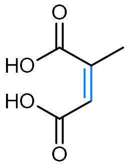 **4a**	YqjM	+	A+B+C	6.4 ± 0.8	3.8 ± 0.3	(*R*) >99	>99
		-	A+B+C	9.1 ± 0.2	0.6 ± 0.3	(*R*) >99	98 ± 1
		-	B+C[a]	5.1	5.2	(*R*) >99	>99
	-	+	A	9.9 ± 0.2	n.d.	n.d.	>99
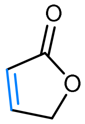 **5a**	OYE1	+	A+B+C	n.d.	9.9 ± 0.3	-	>99
		-	A+B+C	8.8 ± 0.2	1.3 ± 0.4	-	>99
		-	B+C[a]	n.d.	10	-	>99
	-	+	A	10.2 ± 0.1	n.d.	-	>99
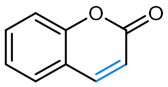 **6a**	XenA	+	A+B+C	4.2 ± 0.4	2.7 ± 0.2	-	69 ± 3
		-	A+B+C	9.3 ± 0.3	<0.2	-	94 ± 2
		-	B+C[a]	0.7	6.3	-	70
	-	+	A	9.4 ± 0.2	n.d.	-	94 ± 2

Module A: Recombinant *Synechococcus elongatus* cells (OD_{750} = 5). **Module B**: *Lk*ADH (CFE, 0.25 mg mL^{-1}, 0.23 $U_{2\text{-propanol}}$ mL^{-1}), $NADP^+$ (0.1 mM). **Shuttle**: Acetone (5 mM). **Module C**: The indicated ERED (purified enzyme, 100 µg mL^{-1}), DMSO (4% v/v), and the indicated substrate **1a**-**6a** (10 mM). **Combination of Modules B+C (coupled enzyme recycling)**: 2-propanol (20 mM) was supplied instead of acetone in Module B. All in BG11 medium (with 5 mM HEPES/NaOH buffer, pH 8; supplemented with 1 mM $MgCl_2$, final volume 1 mL). Reaction overnight (16 h) in a photoreactor with white light (215 µE m^{-2} s^{-1}) at room temperature and 600 rpm. The experiment was performed in two biological replicates, each in triplicate. c. = concentration; n.d. = not detected; [a]3h reaction time, no DMSO cosolvent. [a]**coupled enzyme recycling**: 2-propanol (20 mM) was supplied instead of acetone in Module B.

2.10 Application of the MPS for keto acid dehydrogenases

Table S8. Performance of the MPS (Modules A+B+C) for the reduction of **7a** and **8a** using keto acid dehydrogenases, control reactions and comparison to an ADH-based coupled enzyme recycling system (Modules B+C).

Substrate	Enzyme	Light	Module	Substrate c. [mM]	Product c. [mM]	Product ee [%]	Total Recovery [%]
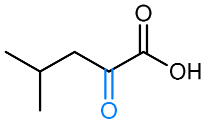 **7a**	D-HicDH	+	A+B+C	n.d.	8.3 ± 0.1	>99 (*R*)	83 ± 1
		-	A+B+C	6.5 ± 0.0	2.7 ± 0.0	>99 (*R*)	92 ± 0
		-	B+C	n.d.	8.6	>99 (*R*)	86
	L-HicDH	+	A+B+C	n.d.	8.2 ± 0.2	>99 (*S*)	82 ± 2
		-	A+B+C	7.2 ± 0.1	2.0 ± 0.0	>99 (*S*)	92 ± 1
		-	B+C[a]	n.d.	8.5	>99 (*S*)	85 ± 2
	-	+	A	9.1 ± 0.1	<0.2	-	91 ± 1
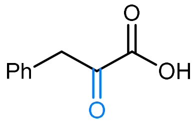 **8a**	D-HicDH	+	A+B+C	n.d.	9.2 ± 0.1	>99 (*R*)	92 ± 1
		-	A+B+C	5.7 ± 0.0	2.7 ± 0.0	>99 (*R*)	84 ± 1
		-	B+C[a]	n.d.	9.5 ± 0.0	>99 (*R*)	95 ± 0
	L-HicDH	+	A+B+C	n.d.	8.8 ± 0.3	>99 (*S*)	88 ± 3
		-	A+B+C	6.2 ± 0.1	1.8 ± 0.1	>99 (*S*)	80 ± 1
		-	B+C[a]	n.d.	9.2 ± 0.0	>99 (*S*)	92 ± 0
	-	+	A	6.6 ± 0.2	n.d.	-	66 ± 2

Module A: Recombinant *Synechococcus elongatus* cells (OD_{750} = 5). **Module B**: *Lk*ADH (CFE, 0.25 mg mL^{-1}, 0.23 $U_{2\text{-propanol}}$ mL^{-1}), $NADP^+$ (0.1 mM). **Shuttle**: Acetone (5 mM). **Module C**: L-HicDH (CFE, 0.5 mg mL^{-1}; 1.8 U_{8a} mL^{-1}) or D-HicDH (CFE, 0.5 mg mL^{-1}; 4.7 U_{8a} mL^{-1}), and the indicated substrate (10 mM, **7a** was added as stock solution in BG11, pH adjusted to 7.5; **8a** was added as stock solution in DMSO, final v/v 4%) and the corresponding reactions were supplemented with 100 mM HEPES/NaOH pH 8. **Combination of Modules B+C (coupled enzyme recycling)**: 2-propanol (20 mM) was supplied instead of acetone in Module B. All in BG11 medium (with 5 mM HEPES/NaOH buffer, pH 8; supplemented with 1 mM $MgCl_2$, final volume 1 mL). Reaction overnight (16 h) in a photoreactor with white light (215 µE m^{-2} s^{-1}) at room temperature and 600 rpm. The experiment was performed in two biological replicates, each in triplicate. c. = concentration; n.d. = not detected. [a]**coupled enzyme recycling**: 2-propanol (20 mM) was supplied instead of acetone in Module B.

2.11 Application of the MPS for imine reductases

Table S9. Performance of the MPS (Modules A+B+C) for the reduction of **9a-11a** using IREDs, control reactions and comparison to an ADH-based coupled enzyme recycling system (Modules B+C).

Substrate	Enzyme	Light	Modules	Substrate c. [mM]	Product c. [mM]	Product ee [%]	Total Recovery [%]
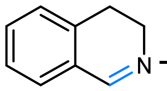	IRED A	+	A+B+C	0.7 ± 0.2	8.1 ± 0.4	-	88 ± 7
		-	A+B+C	10.4 ± 0.1	0.6 ± 0.0	-	>99
		-	B+C[a]	<0.2	8.9	-	90
	IRED J	+	A+B+C	0.6 ± 0.4	8.3 ± 0.3	-	89 ± 7
		-	A+B+C	9.2 ± 0.1	0.8 ± 0.0	-	>99
**9a**		-	B+C[a]	<0.2	8.6	-	86
	-	+	A	9.7 ± 0.1	n.d.	-	97 ± 1
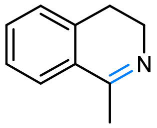	IRED A	+	A+B+C	0.5 ± 0.2	9.2 ± 0.3	36 (*R*)	98 ± 5
		-	A+B+C	9.3 ± 0.2	0.7 ± 0.0	n.d.	>99
		-	B+C[a]	0.2	9.6	19 (*R*)	98
	IRED J	+	A+B+C	1.0 ± 0.5	8.9 ± 0.4	99 (*S*)	9.9 ± 0.9
		-	A+B+C	9.1 ± 0.2	0.7 ± 0.0	n.d.	9.8 ± 0.2
**10a**		-	B+C[a]	0.4	9.3	99 (*S*)	9.7
	-	+	A	10.2 ± 0.2	n.d.	n.d.	10.2 ± 0.2
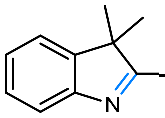	IRED A	+	A+B+C	6.2 ± 0.5	3.4 ± 0.8	44 (*R*)	95 ± 13
		-	A+B+C	9.5 ± 0.1	0.6 ± 0.0	50 (*R*)	>99
		-	B+C[a]	2.5	7	42 (*R*)	95
	IRED J	+	A+B+C	8.0 ± 0.5	2.0 ± 0.2	12 (*R*)	>99
		-	A+B+C	10.6 ± 0.1	<0.2	11 (*R*)	>99
**11a**		-	B+C[a]	5.2	4.3	26 (*R*)	95
	-	+	A	9.9 ± 0.1	n.d.	-	>99

Module A: Recombinant *Synechococcus elongatus* cells (OD_{750} = 5). **Module B**: *Lk*ADH (CFE, 0.25 mg mL^{-1}, 0.23 $U_{2\text{-propanol}}$ mL^{-1}), $NADP^+$ (0.1 mM). **Shuttle**: Acetone (5 mM). **Module C**: The indicated IRED (CFE, 4 mg mL^{-1}), DMSO (5% v/v), and the indicated substrate **9a**, **10a** or **11a** (10 mM). All in BG11 medium (with 5 mM HEPES/NaOH buffer, pH 8; supplemented with 1 mM $MgCl_2$, final volume 1 mL). Reaction overnight (16 h) in a photoreactor with white light (215 µE m^{-2} s^{-1}) at room temperature and 600 rpm. The experiment was performed in two biological replicates, each in triplicate. c. = concentration; n.d. = not detected. [a]**coupled enzyme recycling**: 2-propanol (20 mM) was supplied instead of acetone in Module B.

2.12 Application of the MPS for a Baeyer Villiger monooxygenase (CHMO)

Table S10. Performance of the MPS (Modules A+B+C) for the monooxygenation of **12a** using a BVMO (CHMO), control reactions and comparison to an ADH-based coupled enzyme recycling system (Modules B+C).

Substrate	Enzyme	Light	Module	Substrate	Products		Total Recovery
				c. [mM]	alcohol **12c** c. [mM]	lactone **12b** c. [mM]	[%]
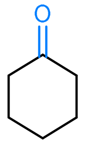	CHMO	+	A+B+C	<0.2	<0.2	8.4 ± 0.1	86 ± 1
		-	A+B+C	7.3 ± 0.2	1.3 ± 0.0	0.3 ± 0.0	89 ± 2
		+	A+B+C[a]	n.d.	n.d.	8.5 ± 0.1	85 ± 1
		-	B+C	8.4 ± 0.1	n.d.	n.d.	84 ± 1
		-	B+C[b]	0.2	2.0	7.3	95
**12a**	-	+	A	0.2 ± 0.0	10.1 ± +0.2	n.d.	>99
		+	A[c]	n.d.	n.d.	9.5 ± 0.1	95 ± 1

Module A: Recombinant *Synechococcus elongatus* cells (OD_{750} = 5). **Module B**: *Lk*ADH (CFE, 0.25 mg mL^{-1}, 0.23 $U_{2\text{-propanol}}$ mL^{-1}), $NADP^+$ (0.1 mM). **Shuttle**: Acetone (5 mM). **Module C**: CHMO (CFE, 2 mg mL^{-1}, 0.22 U_{12a} mL^{-1}), and **12a** (10 mM). All in BG11 medium (with 5 mM HEPES/NaOH buffer, pH 8; supplemented with 1 mM $MgCl_2$, final volume 1 mL). Reaction overnight (16 h) in a photoreactor with white light (215 µE m^{-2} s^{-1}) at room temperature and 600 rpm. The experiment was performed in two biological replicates, each in triplicate. c. = concentration; n.d. = not detected; [a]Acetone omitted from Module B. [b]**(coupled enzyme recycling)**: 2-propanol (20 mM) was supplied instead of acetone in Module B. [c]Recombinant *Synechococcus elongatus* incubated with lactone **12b**, only.

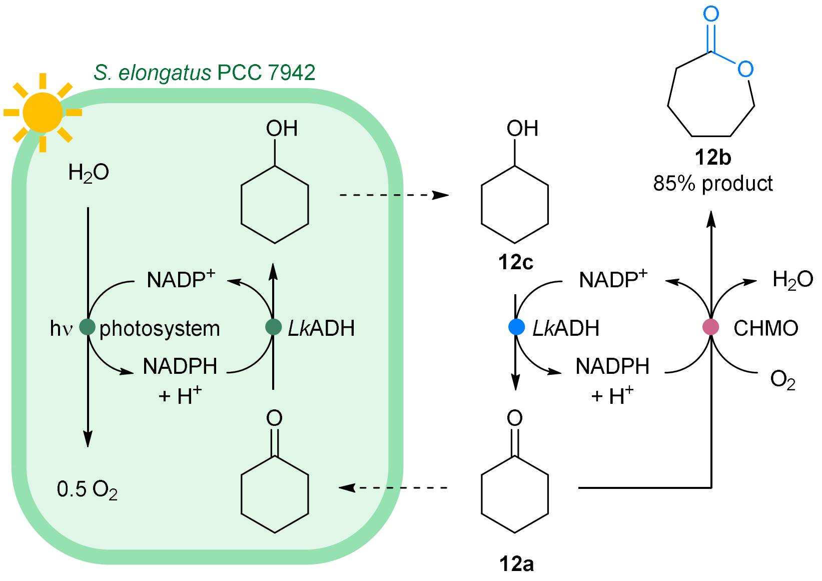

Figure S3. Detailed reaction scheme of the modular photosynthetic monooxygenation of **12a**, using the substrate as alcohol/ketone shuttle pair.

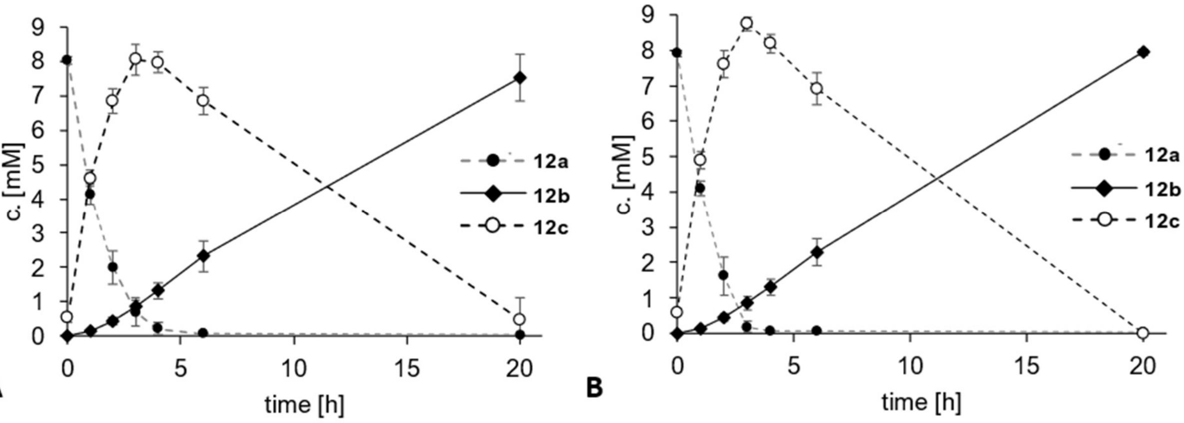

A B

Figure S4. Time course of the CHMO-catalyzed lactonization of **12a**, applying the fully assembled regeneration system. **(A)** optimized conditions with acetone as alcohol/ketone shuttle. **(B)** optimized conditions without the addition of an alcohol/ketone shuttle pair. **Module A**: Recombinant *Synechococcus elongatus* cells (OD_{750} = 5). **Module B**: *Lk*ADH (CFE, 0.25 mg mL^{-1}), $NADP^+$ (0.1 mM). **Shuttle**: If indicated: acetone as ketone-shuttle (5 mM). **Module C**: CHMO (CFE, 2 mg mL^{-1}), and **12a** (10 mM). **Combination of Modules B+C (coupled enzyme recycling)**: 2-propanol (20 mM) was supplied instead of acetone in Module B. All in BG11 medium (with 5 mM HEPES/NaOH buffer, pH 8; supplemented with 1 mM $MgCl_2$, final volume 1 mL). Reaction overnight (16 h) in a photoreactor with white light (215 µE m^{-2} s^{-1}) at room temperature and 600 rpm. The experiment was performed in three biological replicates. c. = concentration.

3 Supplementary Methods

3.1 General, kits, instruments and chemicals

Optical densities of *E. coli* cell cultures and enzyme concentrations of enzyme preparations were determined on an Eppendorf Biophotometer Plus. Optical densities and chlorophyll *a* contents of cyanobacterial cell cultures were measured on a Cary 60 UV-Vis photometer from Agilent Technologies. SDS page was performed with Gene Script ExpressPlus™ page gels. Cell disruption was carried out with a BRANSON Digital Sonifier. An A&D GH-200 semi-micro analytical balance was used for the preparation of samples and stock solutions. Measurements for determining enzyme activity were done using a SpectraMax M2 microplate-reader. General reagents, substrates and solvents were purchased and used as supplied from Sigma-Aldrich (Merck KGaA), Thermo Fisher Scientific, Fluka, Lancaster and Roth. Unless stated otherwise, reagents and organic solvents were obtained from commercial suppliers in reagent grade quality and used without further purification.

3.2 Synthesis of *rac*-2b

2a (209.3 mg, 1.1 mmol) was dissolved in ethyl acetate (20 mL) and stirred under hydrogen atmosphere at atmospheric pressure at room temperature using 10% Pd/C (12.1 mg) as catalyst. After 24 h, the mixture was filtered through celite and evaporated to afford pure white crystals, yield: 92%. ^{1}H NMR (300 MHz, Chloroform-*d*) δ 7.50 – 7.20 (m, 5H), 3.15 – 2.90 (m, 2H), 2.57 – 2.36 (m, 1H), 1.42 (d, *J* = 7.0 Hz, 3H). ^{13}C NMR (75 MHz, $CDCl_3$) δ 179.7, 175.6, 132.1, 129.3, 128.7, 126.6, 36.8, 35.0, 17.1.

3.3 Source of organisms

Synechocystis sp. PCC 6083 wild-type (substrain Kazusa, geographical origin in California (USA)) was received from Prof. Tamagnini at the University of Porto.[1-3] The wild-type and recombinant *Synechococcus elongatus* PCC 7942 (alcohol dehydrogenase from *L. kefir* under the P_{psbA1} promoter and spectinomycin resistance cassette integrated into the neutral site 1 (NS1) of the chromosome) were received from Prof. Waginkar at the Indian Institute of Technology Bombay.[4]

3.4 Enyzmes

The enzymes used in this study are enlisted in Table S11.

Table S11. Enzymes used in this study.

Type	Name	ACC	Origin	Literature
ADH	*Lk*ADH	Q6WVP7	*Lactobacillus kefir*	[5]
	ADH-A	Q8KLT9	Rhodococcus ruber DSM 44541	[6-8]
ERED	ChrOYE1	A0A0U2H4S5	*Chryseobacterium* sp. CA49	[9]
	*Dr*ER	Q9RSD4	*Deinococcus radiodurans*	[10]
	NCR	Q5NLA1	*Zymomonas mobilis* subsp. *mobilis*	[11]
	NerA	O31246	*Rhizobium radiobacter*	[12]
	OPR3	Q9FEW9	*Lycopersicon esculentum*	[13-14]
	OYE1	Q02899	*Saccharomyces pastorianus*	[15]
	*Pp*XenB	Q88PD0	*Pseudomonas putida*	[16-17]
	*Rm*ER	Q1LDQ5	*Cupriavidus metallidurans*	[10]
	*Ts*OYE	B0JDW3	*Thermus scotoductus* SA-01	[18]
	XenA	Q9R9V9	*Pseudomonas putida*	[19]
	XenB	Q9RPM1	*Pseudomonas fluorescens*	[19]
	YqiG	P54524	*Bacillus subtilis str. 168*	[20]
	YqjM	P54550	*Bacillus subtilis*	[21-22]
	*Lac*ER	S4ZS69	*Lactobacillus casei* str. Zhang	[23]
IRED	IRED A	M4ZRJ3	*Streptomyces* sp. GF3587	[24-25]
	IRED J	D2PR38	*Kribbella flavida* DSM 17836	[24-25]
Keto Acid Dehydrogenase	L-HicDH	P14295	*Lactobacillus confusus* DSM 20196	[26-27]
	D-HicDH	P17584	*Lactobacillus paracasei* DSM 20008	[27-28]
BVMO	CHMO	P12015	*Acinetobacter* sp. NCIMB 9871	[29-31]

3.5 Expression of enzymes in *E. coli*

The enzymes used in this study were expressed in *E. coli* BL21(DE3) or its derivatives (Table S13).

3.5.1 Transformation

Plasmids (100 ng) were mixed with chemically competent *E. coli* BL21 (DE3) cells (100 µL), rested on ice for 30 minutes and heat-shocked for 30 seconds at 42 °C. SOC medium (200 µL) was added, and the transformed cells were incubated for 1 h at 37 °C and 300 rpm. The cells were then plated on a LB-agar plate supplemented with corresponding antibiotic (ampicillin, 100 µg mL^{-1} or kanamycin, 50 µg mL^{-1}) and incubated overnight at 37 °C.

3.5.2 Cultivation

Overnight cultures (ONC) were prepared in LB-medium (10 mL) supplemented with the corresponding antibiotic at 30 °C and 120 rpm. The ONCs were then used for the inoculation of sterile medium (1% v/v) supplemented with the corresponding antibiotic. Precultures were incubated until OD_{600} of 0.6 was reached, then protein expression was induced, and cells incubated further. Detailed conditions for every enzyme can be found in Table S13.

3.5.3 Harvesting

To harvest the cells, cultures were centrifuged at 3184 *g*, 20 min, 4 °C, the cell pellet suspended in wash buffer (1 - 2.5 g cells per 10 mL phosphate buffer, 10 mM, pH 7), and then centrifuged again under the same conditions.

3.5.4 Preparation of cell-free extracts

Cell pellets were suspended in lysis buffer and sonicated on ice (for conditions see Table S12). The sonicated cells were centrifuged for 25 minutes at 17 000 *g* and 4 °C. The supernatant (cell-free extract) was shock-frozen (liquid nitrogen) inside a round bottom flask, lyophilized, and stored at -20 °C.

Table S12. Buffers and sonication parameters used for preparations of CFEs.

Enzyme	Sonication Parameters	Lysis Buffer
*Lk*ADH	5 min, amplitude 30 %, 1 sec ON, 4 sec OFF	KPi (50 mM, $MgCl_2$ 1 mM, pH 7.5)
ADH-A		KPi (50 mM, pH 7)
LeifADH		
HLADH		
L-HicDH		
D-HicDH		
CHMO		KPi (50 mM, 0.3 M NaCl, pH 8)
IRED A	2.5 min, amplitude 30%, 2 sec ON, 4 sec OFF	Tris/HCl (100 mM, pH 7.5)
IRED J		

The pH of the buffers was adjusted by using hydrochloric acid (HCl) and sodium hydroxide (NaOH).

3.5.5 SDS-PAGE

Protein concentrations of cell pellets and supernatants were determined *via* a Bradford Assay. Volumes equivalent to 15 µg of protein were mixed with Laemmli sample buffer (1:1) and heated to 95 °C for 5 minutes. Prepared samples and a marker (PageRuler Prestained Protein Ladder; 7 µL) were loaded onto the 10 % SDS-PAGE gel (100 V, MOPS buffer). The gel was stained overnight using Coomassie Quick Stain and afterwards destained with deionized water.

Table S13. Cultivation conditions for the expression in *E. coli*.

pEG	Enzyme Name	Form	Plasmid Backbone	Size [kDa]	Tag	-*C* or -*N* Terminus	*E. coli* Host Strain	Medium	Antibiotic	c_{final} [µg mL^{-1}]	Inducer	c_{final}	Preculture		Expression		
													T [°C]	rpm	T [°C]	rpm	time
10	ADH-A	CFE	pET22b	36	none		BL21(DE3)	LB[a]	Amp	100	IPTG[b]	450 mg mL^{-1}	30	120	20	120	24 h
518	ADH-A	pure	pET21a	36	Strep	*N*	BL21(DE3)	LB[a]	Amp	100	IPTG[b]	2 mM	30	120	20	120	24 h
326	*Lk*ADH	CFE	pET21a	27	none		BL21(DE3)pLys	LB	Amp[c]	100	IPTG	1 mM	37	120	30	120	ON
524	*Lk*ADH	pure	pET28a	27	His	*N*	BL21(DE3)	LB	Kan	50	IPTG	1 mM	37	120	30	120	ON
54	HLADH	CFE	pET28a	42	His	*N*	BL21(DE3)	LB	Kan	50	IPTG	0.5 mM	30	120	25	120	ON
293	LeifADH	CFE	IBA7+	26	Strep	*N*	BL21(DE3)	LB	Amp	100	AHTC	0.2 µg mL^{-1}	30	120	25	120	ON
	*Chr*OYE1	pure	pET28a	39	His	*N*	BL21(DE3)	LB	Kan	50	IPTG	0.2 mM	37	120	20	120	ON
	*Dr*ER	pure	pET28a	40	His	*N*	BL21(DE3)	LB	Kan	50	IPTG	0.2 mM	37	120	20	120	ON
516	NCR	pure	pET28a	40	His	*N*	BL21(DE3)	LB	Kan	50	IPTG	0.2 mM	37	120	20	120	ON
147	NerA	pure	pET21a	40	His	*C*	BL21(DE3)	LB	Amp	100	IPTG	0.2 mM	37	120	20	120	ON
367	OPR3	pure	pET21a	45	His	*N*	BL21(DE3) RIL	LB	Amp	50	IPTG	0.2 mM	37	140	37	140	ON
360	OYE1	pure	pET28a	45	His	*C*	BL21(DE3)	LB	Kan	50	IPTG	0.2 mM	37	120	20	120	ON
	*Pp*XenB	pure	pET28a	38	His	*N*	BL21(DE3)	LB	Kan	50	IPTG	0.2 mM	37	120	20	120	ON
	*Rm*ER	pure	pET28a	40	His	*N*	BL21(DE3)	LB	Kan	50	IPTG	0.2 mM	37	120	20	120	ON
	*Ts*OYE	pure	pET28a	38	His	*N*	BL21(DE3)	LB	Kan	50	IPTG	0.2 mM	37	120	20	120	ON
145	XenA	pure	pET21a	40	His	*C*	BL21(DE3)	LB	Amp	100	IPTG	0.2 mM	37	120	20	120	ON
146	XenB	pure	pET21a	38	His	*C*	BL21(DE3)	LB	Amp	100	IPTG	0.2 mM	37	120	20	120	ON
	YqiG	pure	pET28a	41	His	*N*	BL21(DE3)	LB	Kan	50	IPTG	0.2 mM	37	120	20	120	ON
368	YqjM	pure	pET28a	38	His	*N*	BL21(DE3)	LB	Kan	50	IPTG	0.2 mM	37	120	20	120	ON
	*Lac*ER	pure	pET28a	42	His	*N*	BL21(DE3)	LB	Kan	50	IPTG	0.2 mM	37	120	20	120	ON
221	D-HicDH	CFE	pET21a	37	none		BL21(DE3)	LB	Amp	100	IPTG	1 mM	37	120	25	120	ON
220	L-HicDH	CFE	pET21a	33	none		BL21(DE3)	LB	Amp	100	IPTG	1 mM	37	120	25	120	ON
373	IRED A	CFE	pET28a	33	His	*N*	BL21(DE3)	TB	Kan	50	IPTG	1 mM	30	120	20	120	ON
382	IRED J	CFE	pET28a	33	His	*N*	BL21(DE3)	TB	Kan	50	IPTG	1 mM	30	120	20	120	ON
87	CHMO	CFE	pET21a	61	His	*C*	BL21(DE3)	LB	Amp	100	IPTG	1 mM	37	130	17	120	ON

Amp: ampicillin (c_{stock} = 100 mg mL^{-1}, in H_2O); Kan: kanamycin (c_{stock} = 50 mg mL^{-1}, in H_2O); AHTC: anhydrotetracycline (c_{stock} = 2 mg mL^{-1}, in ethanol); IPTG: isopropyl-*β*-D-thiogalactopyranoside (c_{stock}= 1M, in H_2O); LB: NaCl (5 g L^{-1}), yeast extract (5 g L^{-1}), tryptone (10 g L^{-1}); TB: A: yeast extract (24 g L^{-1}), tryptone (12 g L^{-1}), glycerol (4 mL L^{-1}), B: KH_2PO_4 (2. 31 g L^{-1}), K_2HPO_4 (12.54 g L^{-1}), A and B autoclaved separately; CFE: cell-free extract; ON: overnight. a: $ZnCl_2$ (100 mg L^{-1}), Amp, K_2HPO_4 (4.4 g L^{-1}) and KH_2PO_4 (1.4 g L^{-1}) added as solids to sterile LB. b: Induction after 24 h (OD_{600} = 5), IPTG and additional Amp (50 mg mL^{-1}) added as solids. c: Chloramphenicol (c_{final} = 50 µg mL^{-1}) added to the ONC, only.

3.6 Preparation of purified enzymes

Buffers and sonication conditions used are listed in Table S14.

3.6.1 Cell lysis

Harvested cell pellets were resuspended in binding buffer (ene-reductases were supplemented with a spatula tip of FMN) and sonicated on ice. (Digital sonifier, BRANSON). The cell suspension was centrifuged (20 min, 18 000 *g*, 4 °C) and the supernatant was filtered (0.45 µm syringe filter) and stored on ice.

3.6.2 Strep-tag purification

ADH-A with the Strep-Tag was purified by strep-tactin affinity chromatography (Strep-Tactin®XT Superflow® Column, IBA) with gravity flow. The purification was performed at 4 °C. The column was equilibrated with two column volumes binding buffer, the filtered lysate was loaded onto the column and the flow-through collected for SDS-PAGE-sample. The column was washed five times with one column volume of binding buffer. Then, the protein was eluted by applying eight times 0.5 column volumes elution. After finished elution the column was regenerated with four column volumes sodium hydroxide (10 mM NaOH in water). NaOH was removed immediately by washing the column two times with four column volumes binding buffer. The column was stored at 4 °C.

3.6.3 His-tag purification

*Lk*ADH and the ene-reductases containing the His_6-tag were purified by an immobilized metal ion affinity chromatography (HisTrapTM FF, 5 mL, GE HEALTHCARE). The purification was performed at 4 °C and at a flow rate of 5 mL min^{-1}. Prior to loading the soluble fraction to a HisTrap FF column (GE Healthcare, 5mL) equilibrated in binding buffer it was filtered through a 45 µm syringe filter. After loading the column was washed with binding buffer according to the manual. Then, the enzyme was eluted using elution buffer. All purification steps were verified by SDS PAGE.

3.6.4 Storage

The volume of the fractions containing the enzyme (colored yellow or visualized with Bradford reagent) was reduced to 2.5 mL using a Vivaspin® 20 mL Ultrafiltration Unit (Satorius). Then, the buffer was changed to storage buffer using a Sephadex G-25 PD10 desalting column (GE Healthcare). The final enzyme solution was aliquoted and stored at -20 °C.

Table S14. Buffers and sonication parameters used for the purification of enzymes.

Enzyme	Binding Buffer	Elution Buffer	Storage Buffer	Sonication Parameters
ADH-A	100 mM Tris/HCl, 150 mM NaCl, pH 8; 10 mL g^{-1} pellet	100 mM Tris/HCl, 150 mM NaCl, 50 mM biotin, pH 8	50 mM KPi; Na_2HPO_4 x $2H_2O$ (7.58 g L^{-1}), KH_2PO_4 (1.01 g L^{-1})	3 x 2.5 min, amplitude 30%, 2 sec ON, 4 sec OFF, 1 min pause
*Lk*ADH	50 mM Tris/HCl, 150 mM NaCl, 20 mM imidazole, pH 7.5, 1 mM $MgCl_2$, pH 7.5; 10 mL g^{-1} pellet	50 mM Tris/HCl, 150 mM NaCl, 250 mM imidazole, pH 7.5, 1 mM $MgCl_2$, pH 7.5	50 mM KPi; Na_2HPO_4 x $2H_2O$ (7.58 g L^{-1}), KH_2PO_4 (1.01 g L^{-1}), 1 mM $MgCl_2$	
ene-reductases	20 mM sodium phosphate, 500 mM NaCl, 20 mM imidazol, pH 7.4; 5 mL g^{-1} pellet	20 mM sodium phosphate, 500 mM NaCl, 500 mM imidazol, pH 7.4	50 mM sodium phosphate, pH 7.4	3.5 min, amplitude 30%, 2 sec ON, 4 sec OFF

The pH of the buffers was adjusted by using hydrochloric acid (HCl) and sodium hydroxide (NaOH). Buffers used for purification were degassed using ultrasonication and filtered with a Steritop® Filter (Millipore Express® PLUS, 0.22 µm PES Membrane).

3.7 Cultivation of cyanobacteria

Cyanobacteria were cultivated in BG11 medium[1] supplemented with HEPES (5 mM, pH 8) at 30 °C under a 16-hour light (Lumitronix, Powerbar V3 cool white 5700K)/8-hour dark regimen. Seed cultures were grown in of BG11 (30 mL) in baffled 100 mL Erlenmeyer flasks under shaking at 180 rpm and illumination (30 µE m^{-2} s^{-1}) for 5-9 days. Working cultures were inoculated from seed cultures to an OD_{750} of 0.1 in BG11 (150 mL), inside 250 mL gas-washing flasks and grown under 80 µE m^{-2} s^{-1} light-intensity while continuously bubbling with water-saturated air, sterilized by pumping through a venting filter (0.2 µm pore size, Midisart 2000) until reaching an OD_{750} of 1-2 (ca. 5 days). Seed cultures of recombinant *Synechococcus elongatus* were grown in the presence of spectinomycin (100 µg mL^{-1}).

3.8 Determination of the cell dry weight and chlorophyll *a* content

The dry cell weight and the amount of chlorophyl *a* were determined from samples originating from at least three independent cultivations under growth conditions for working cultures, each measured in triplicates.

3.8.1 Cell dry weight

Working cultures grown and harvested as above were shock-frozen in liquid nitrogen, lyophilized overnight and weighed in three independent experiments.

3.8.2 Chlorophyll *a*

The chlorophyll *a* was determined as described.[32] A sample of the cell culture (100 µL) was mixed with cold methanol (900 µL) and incubated in darkness at 4 °C for 2-3 hours or overnight. Then, the samples were centrifuged for 3 min at 14000 g and the absorption of the supernatant was measured at 665 nm. The amount of chlorophyll was determined using the extinction coefficient ε = 78.74 L g^{-1} cm^{-1} according to Eq. S 1, where the dilution factor corresponds to 10.

Eq. S1 $$Chl\ a\ \left[\frac{\mu g}{mL}\right] = A_{665\ nm} * 12.7 * dilution\ factor$$

3.9 General procedure for the fully assembled cofactor recycling system (Modules A+B+C)

Stock solutions of substrates **1a, 4a, 7a** and **12a** were prepared in BG11 (100 mM or 250 mM, final concentration in reaction mixture 10 mM). The pH of stock solutions of **4a** and **7a** was adjusted to 7.5 with aqueous HCl or NaOH. Stock solutions of **2a**, **3a**, **5a**, **6a**, **8a**, **9a**, **10a** and **11a** were prepared in DMSO (250 or 200 mM, final concentration 10 mM, final DMSO content 4% or 5% v/v, respectively). Stock solutions of *Lk*ADH CFE, $NADP^+$, OPR3, $MgCl_2$ and acetone were prepared in BG11 according to Table S15 (or as indicated at the specific experiments). Reaction mixtures with substrate **8a** were supplemented with HEPES (final c. 100 mM, pH 8). Working cultures of *Synechococcus elongatus* at OD_{750} between 1 and 2 were harvested by centrifugation (30 °C, 45 min, 2786 *g*) and the pellet was resuspended in BG11 to reach a stock OD_{750} (20 to 40). The reactions were performed in 1.5 mL screw-top glass GC-vials. First the substrate stock was added, then BG11 and other components, with the cyanobacteria being added last. Table S15 enlists the sample preparation for reduction of **2a** under the optimized conditions.

Table S15. Concentrations of the stock solutions for the fully assembled cofactor recycling system using the optimized conditions for the reduction of **2a**.

**Component**	**Stock**			c_{final}
	c_{stock}	solvent/medium	V [µL mL^{-1}]	
**2a**	250 mM	DMSO	40	10 mM
***Lk*ADH CFE**	5 mg mL^{-1}; 4.6 $U_{2\text{-propanol}}$ mL^{-1}	BG11	50	0.25 mg mL^{-1}; 0.23 $U_{2\text{-propanol}}$ mL^{-1}
**$NADP^+$**	2 mM	BG11	50	0.1 mM
**OPR3**	2 mg mL^{-1}	BG11	50	0.1 mg mL^{-1}
**acetone**	100 mM	BG11	50	5 mM
**$MgCl_2$**	100 mM	BG11	10	1 mM
Synechococcus elongatus	OD_{750} = 20	BG11	250	OD_{750} = 5
**BG11**	-	-	500	-

The vials were incubated in a custom photoreactor[33] equipped with cool white LEDs (LED stripes, 5200 K) for the indicated reaction time (16 h) at 600 rpm shaking and at room temperature at an average light intensity of 215 µE m^{-2} s^{-1} (photoreactor settings: frequency 100 Hz, duty range 100, duty cycle 5) or covered with aluminum foil for dark reactions. The workup and analytics are described in section 4.

3.10 50 mL scale reduction of 2a, utilizing the MPS.

2a (188.7 mg, 1 mmol) was added to a 250 mL Erlenmeyer flask with a screw neck. BG11 (31.4 mL), *Lk*ADH CFE (5 mg mL^{-1}, 2.5 mL, final 0.25 mg mL^{-1}), $NADP^+$ (2 mM, 2.5 mL, final 0.1 mM), OPR3 (8.7 mg mL^{-1}, 574.7 µL, final 100 µg mL^{-1}) and $MgCl_2$ (100 mM, 500 µL, final 1 mM), all dissolved in BG11 were added to the flask. Then, acetone (58.1 µL, final 20 mM) and recombinant *Synechococcus elongatus* (harvested at OD_{750} 1.9, concentrated to OD_{750} = 40, 12.5 mL, final OD_{750} = 9) were added, the flask was closed and placed in an incubator at 24 °C (AQUALYTIC Thermostatically controlled incubators) equipped with a custom photoreactor[33] controlling four cool white LED stripes (5200 K). Two LED stripes were mounted on the left and right wall of the incubator and two LED stripes were mounted above the reaction, on the rack placed in the 7th slot from the bottom, giving an average light intensity of 300 µE m^{-2} s^{-1} at the spot of the reaction (photoreactor settings: frequency 100 Hz, duty range 100, duty cycle 90). The reaction was shaken at 140 rpm. The setup is displayed in the main paper. After 16 h, the mixture was extracted with ethyl acetate (2 x 150 mL), dried over anhydrous Na_2SO_4, and concentrated on the rotary evaporator. The crude was purified with Biotage® Selekt Flash Purification System using Biotage® Sfär Silica HC Duo column (20 µm, 5 g) and gradient elution starting from cyclohexane to 30% ethyl acetate in twenty column volumes, then to 50% ethyl acetate in five column volumes, yielding 133.4 mg of (*R*)-**2b** (70% isolated yield). ^{1}H NMR (300 MHz, Chloroform-d) d 7.75 – 7.09 (m, 5H), 3.24 – 2.90 (m, 2H), 2.64 – 2.37 (m, 1H), 1.45 (d, J = 7.1 Hz, 3H). The HPLC chromatogram is displayed in the analytics section (Figure S9).

4 Analytics and Chromatographic Data

GC-MS spectra were recorded on an Agilent 7890A GC-system, equipped with an Agilent 5975C quadrupole detector and a HP-5 MS column (30 m x 0.25 mm x 0.25 µm), using helium as carrier gas (flow = 0.5 mL min^{-1}). Temperature program: 40 °C, hold 2 min, 10 °C min^{-1} to 180 °C, hold 1 min. Retention times were: acetophenone: 9.75 min; phenylethanol: 9.64 min; cyclohexanone: 6.72 min; cyclohexanol: 6.55 min; 6-methyl-5-hepten-2-one: 8.37 min; 6-methyl-5-hepten-2-ol: 8.47 min; 2-octanone: 8.44 min; 2-octanol: 8.6 min; 2-chloroacetophenone: 13.15 min; 2-chlorophenylethanol: 13.12 min; 2-methylcyclohexanone: 7.71 min; 2-methylcyclohexanol: 7.62 min; phenylacetone: 10.76 min; 1-phenyl-2-propanol: 10.84 min; 4-methylcyclohexanone: 7.86 min; 4-methylcyclohexanol: 7.66 min; 2-pentanone: 3.35 min, **1a**: 10.8 min, **1b**: 11.3 min.

GC-FID analytics were performed on an Agilent 7890A GC system equipped with a flame ionization detector (FID). Achiral analytics were performed with an HP-5 column (30 m x 0.25 mm x 0.25 µm) or a DB-1701 column (30 m x 0.25 mm x 0.25 µm), using helium as carrier gas (flow = 0.5 mL min^{-1}) (Table S16). Chiral analysis was performed using a CP-Chirasil-DEX CB column (25 m x 0.32 mm x 0.25 µm), a Hydrodex-β-TBDAc (50 m, 0.25 mm) or a Rt®-BDEXse column (30 m x 0.32 mm x 0.25 µm) column using hydrogen as carrier gas (flow = 0.5 mL min^{-1}) (Table S17).

HPLC analyses on a chiral phase were carried out on a *Shimadzu* HPLC system (Communication Bus Module CBM-20 A, Column Oven CTO-20 AC, Degasser DGU-20 A5, Liquid Chromatograph LC-20 AD, Auto sampler SIL-20 AC, Diode Array Detector SPD-M20 A) equipped with a *Daicel* Chiralcel OD-H column (dimensions: 250 mm × 4.6 mm; stationary phase: coated cellulose-tris-(3,5-dimethylphenylcarbamate); particle size 5 µm) using *n*-heptane and 2-propanol as eluents in isocratic elution (Table S 18).

The reaction mixtures containing different shuttle-molecule pairs were extracted twice with ethyl acetate (400 µL, 250 µL), dried over anhydrous sodium sulphate and measured on GC-MS.

The reaction mixtures containing **1**- **3a**, **5a**, **6a**, **9a**-**12a** were extracted twice with ethyl acetate (400 µL, 250 µL) containing (*R*)-limonene (0.05% v/v), *n*-dodecane (10 mM) or *n*-decanol (10 mM) as internal GC standard. The combined organic phases were dried over anhydrous sodium sulphate. The samples were quantified on the GC-FID (Table S16) and the enantiomeric excess was measured on the GC-FID (Table S17) or the HPLC (Table S 18).

The reaction mixtures containing **4a**/**4b**, **7a**/**7b** and **8a**/**8b** were acidified with aqueous HCl (110 µL, 2 M), saturated with sodium chloride, and extracted twice with ethyl acetate (1 x 400 µL, 1 x 250 µL) containing *n*-decanol (10 mM) as internal GC standard. The combined organic phases were dried over anhydrous sodium sulphate. Compounds were quantified on GC-FID as their corresponding

trimethylsilyl esters: For derivatization the organic phase (100 µL) was with mixed with pyridine (60 µL) and BSTFA (60 µL) and heated for 1 h at 60°C and 600 rpm (**4a**/**4b**, **8a**/**8b**) or incubated at room temperature for 20 min (**7a**/**7b**). The enantiomeric excess was analyzed on GC-FID from the corresponding methyl esters: For derivatization the organic phase (100 µL) was mixed with methanol (10 µL) and TMS (5 µL). The retention times are reported in Table S16 and Table S17 respectively.

The absolute configuration of the enantiomeric products were assigned based on published methods (**1b**,[13] **2b**,[13] **10b**,[24] **11b**[24]), enantiopure authentic references (**4b**, **7b**, **8b**) or from the reported enantioselectivity of YqjM[34] (**3b**). Representative chromatograms of standards and biotransformations, as well as calibration curves are displayed below.

Table S16. Methods for quantification of compounds by GC-FID.

GC method	Column	Internal Standard (IS)	Temperature [C°] injector	Temperature [C°] detector	Split Ratio	Injection Volume [µL]	Temperature Program	Retention Times [min]
1	HP5	(*R*)-limonene	250	300	90:1	1	40 °C, hold 2 min, 10 °C min^{-1} to 180 °C, hold 1 min	**1a**: 10.9, **1b**: 11.3; IS: 9.0
2	HP5	(*R*)-limonene	250	300	90:1	1	100 °C, hold 0.5 min, 10 °C min^{-1} to 300 °C	**2a**: 9.7, **2b**: 10.4, IS: 3.4
3	DB-1701	(*R*)-limonene	250	250	50:1	1	80 °C, hold 1 min, 10 °C min^{-1} to 180 °C, hold 1 min	**3a**: 8.4, **3b**: 9.9, IS: 5.3
4[a]	HP5	*n*-decanol	250	300	90:1	1	100 °C, hold 0.5 min, 10 °C min^{-1} to 200 °C	**4a**: 6.7, **4b**: 6.3, IS: 6.9
5[15]	DB-1701	(*R*)-limonene	250	250	50:1	2	60 °C, hold 5 min, 2 °C min^{-1} to 80 °C, hold 5 min, 20 °C min^{-1} to 110 °C, hold 5 min	**5a**: 19.4, **5b**: 18.7, IS: 14.2
6[15]	HP5	(*R*)-limonene	300	300	50:1	1	100 °C, hold 1 min, 10 °C min^{-1} to 280 °C, hold 2 min	**6a**: 8.8, **6b**: 8.1, IS: 3.9
7[a]	DB-1701	*n*-decanol	250	250	90:1	1	40 °C, hold 2 min, 10 °C min^{-1} to 180 °C, hold 1 min	**7a:**12.1, **7b**: 13.1, IS: 14.5
8[a]	HP-5	*n*-decanol	250	300	50:1	1	100 °C, hold 0.5 min, 10 °C min^{-1} to 300 °C	**8a**: 10.8, **8b**: 9.5, IS: 6.9
9[24]	HP-5	*n*-dodecane	300	300	15:1	1	60 °C, hold 0.5 min, 10 °C min^{-1} to 160 °C, 40 °C min^{-1} to 300 °C, hold 1 min	**9a**: 9.7, **9b**: 10.2, IS: 8.9
10[24]	DB-1701	*n*-dodecane	280	280	15:1	1	80 °C, hold 1 min, 10 °C min^{-1} to 250 °C, hold 1 min	**10a**: 10.2, **10b**: 10.1, IS:7.1
11[24]	HP-5	*n*-dodecane	300	300	15:1	1	80 °C, hold 1 min, 10 °C min^{-1} to 250 °C, hold 1 min	**11a**: 8.5, **11b**: 9.1, IS:7.4
12[35]	HP-5	*n*-decanol	300	300	20:1	1	50 °C, 10 °C min^{-1} to 250 °C	**12a**: 4.2, **12c**: 4.1, **12b**: 7.9, IS: 9.2

a: Compounds quantified as trimethylsilyl esters.

Table S17. Methods for determining the enantiomeric excess of compounds by GC-FID.

GC method	Column	Internal Standard (IS)<br>injector	 detector	Temperature [C°]	Split Ratio	Injection Volume [µL]	Temperature Program
ee_1[13]	Chirasil-Dex CB	250	250	20:1	1	90 °C, hold 2 min, 4 °C min^{-1} to 115 °C, 20 °C min^{-1} to 180 °C, hold 2 min	(*R*)-**1b**: 10.7, (*S*)-**1b**: 10.8
ee_3	Rt®-BDEXse	230	250	50:1	1	60 °C, hold 1 min, 5 °C min^{-1} to 180 °C, hold 2 min	(*R*)-**3b**: 20.3, (*S*)-**3b**: 20.5
ee_4[a]	Hydrodex-β-TBDAc	230	250	20:1	1	40 °C, hold 2 min, 4 °C min^{-1} to 120 °C, hold 1 min, 20 °C min^{-1} to 180 °C, hold 3 min	(*R*)-**4b**: 24.3, (*S*)-**4b**: 24.1
ee_7[a]	Chirasil Dex-CB	250	250	50:1	1	60 °C, hold 2 min, 3 °C min^{-1} to 110 °C, 10 °C min^{-1} to 200 °C	(*R*)-**7b**: 15.6, (*S*)-**7b:** 16.1
ee_8[a]	Rt®-BDEXse	230	250	50:1	1	60 °C, hold 1 min, 5 °C min^{-1} to 180 °C, hold 2 min	(*R*)-**8b**: 20.6, (*S*)-**8a**: 20.8

a: Compounds detected as methyl esters.

Table S 18. Methods for determining the enantiomeric excess of compounds by HPLC.

HPLC Method	Column	Oven Temperature [C°]	Eluent<br>*n*-heptane: 2-propanol	 flow rate [mL min^{-1}]	Run Time [min]	Injection Volume [µL]	Integration Wavelength [nm]	Retention Times [min]
ee_2[12]	Chiralcel OD-H	30	95:5	1	35	10	215	(*R*)-**2b**: 27.1, (*S*)-**2b**: 28.9
ee_10[41]	Chiralcel OD-H	30	99:1[a]	0.5	40	10	265	(*R*)-**10b:** 24.7, (*S*)-**10b**: 23.6
ee_11[41]	Chiralcel OD-H	30	99.4:0.6[a]	0.5	20	10	242	**11a**: 16.3, (*R*)-**11b**: 15.2, (*S*)-**11b**: 14.0

a: +0.1% (v/v) diethyl amine.

4.1 NMR

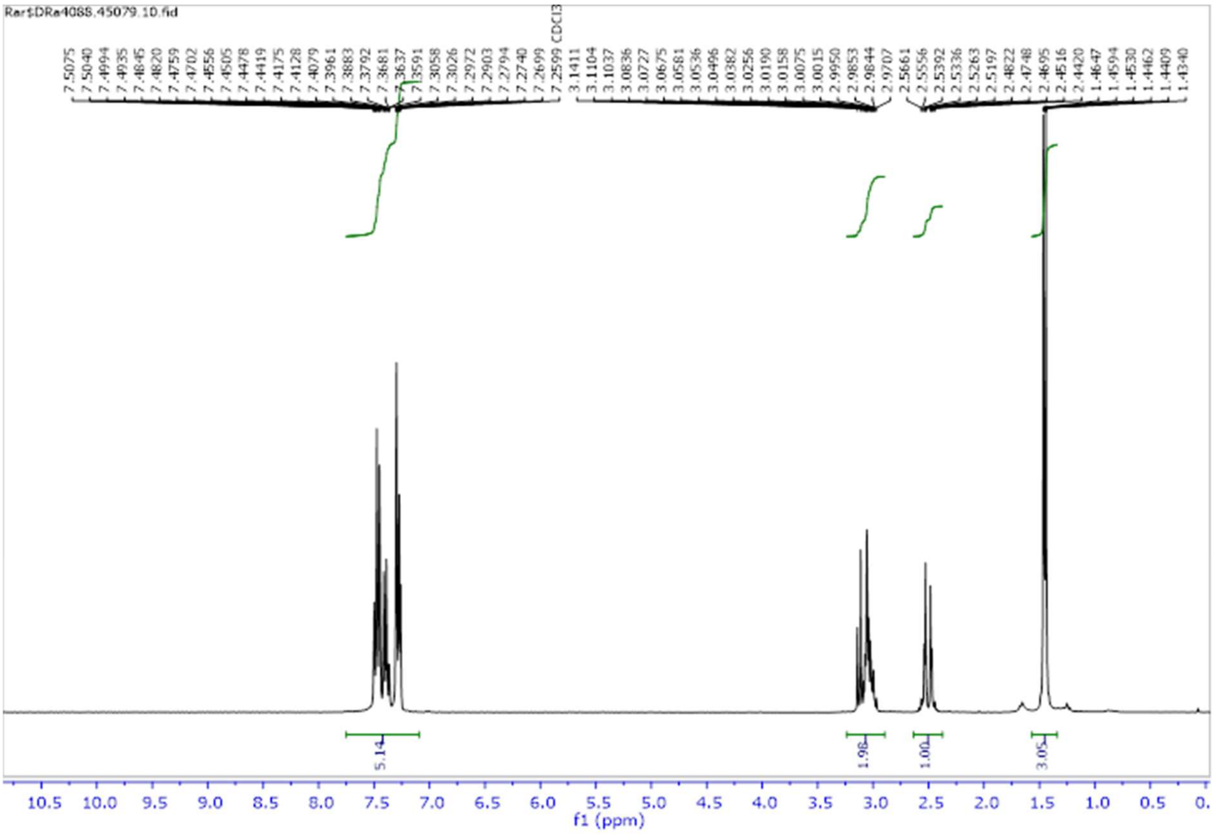

Figure S5. ^{1}H NMR of **2b**, isolated from the 50 mL scale biotransformation.

4.2 Representative chromatograms

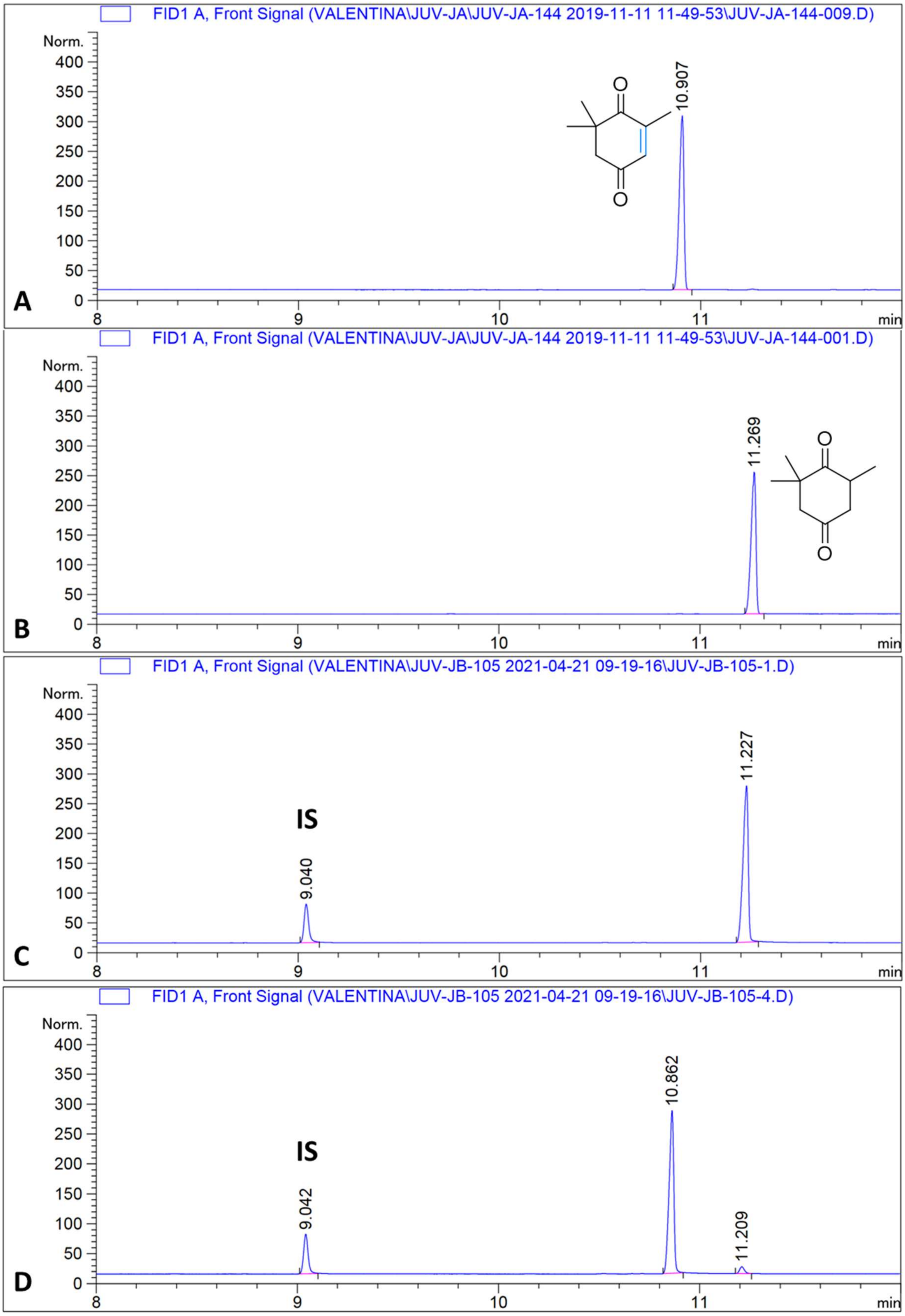

Figure S6. Representative GC-FID chromatograms for quantification of **1a** and **1b**. **(A)** reference compound **1a**; **(B)** reference compound **1b**; **(C)** illuminated and **(D)** dark reduction of **1a** using the MPS.

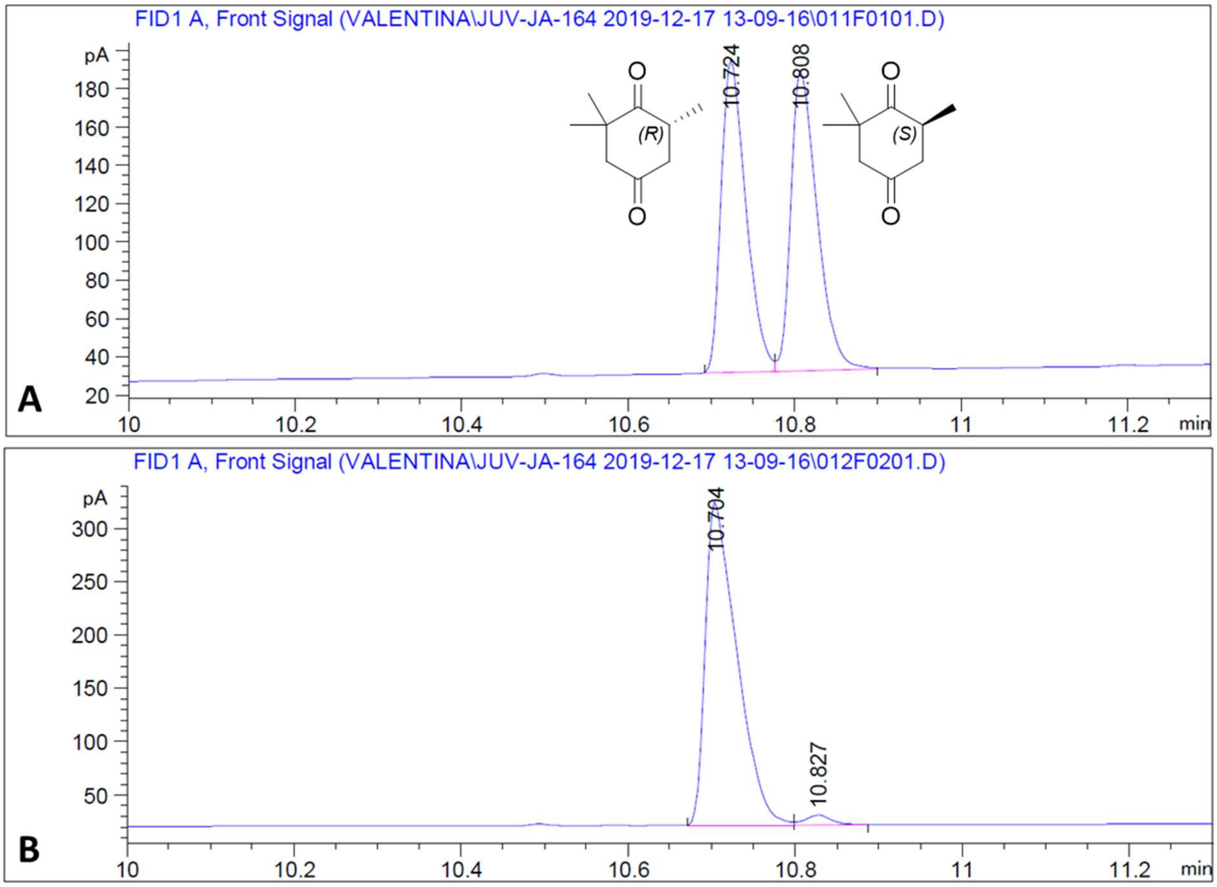

Figure S7. Representative GC-FID chromatograms for determining the enantiomeric excess of **1b**. **(A)** Racemic reference compound; **(B)** illuminated reduction of **1a** to **1b** using the MPS.

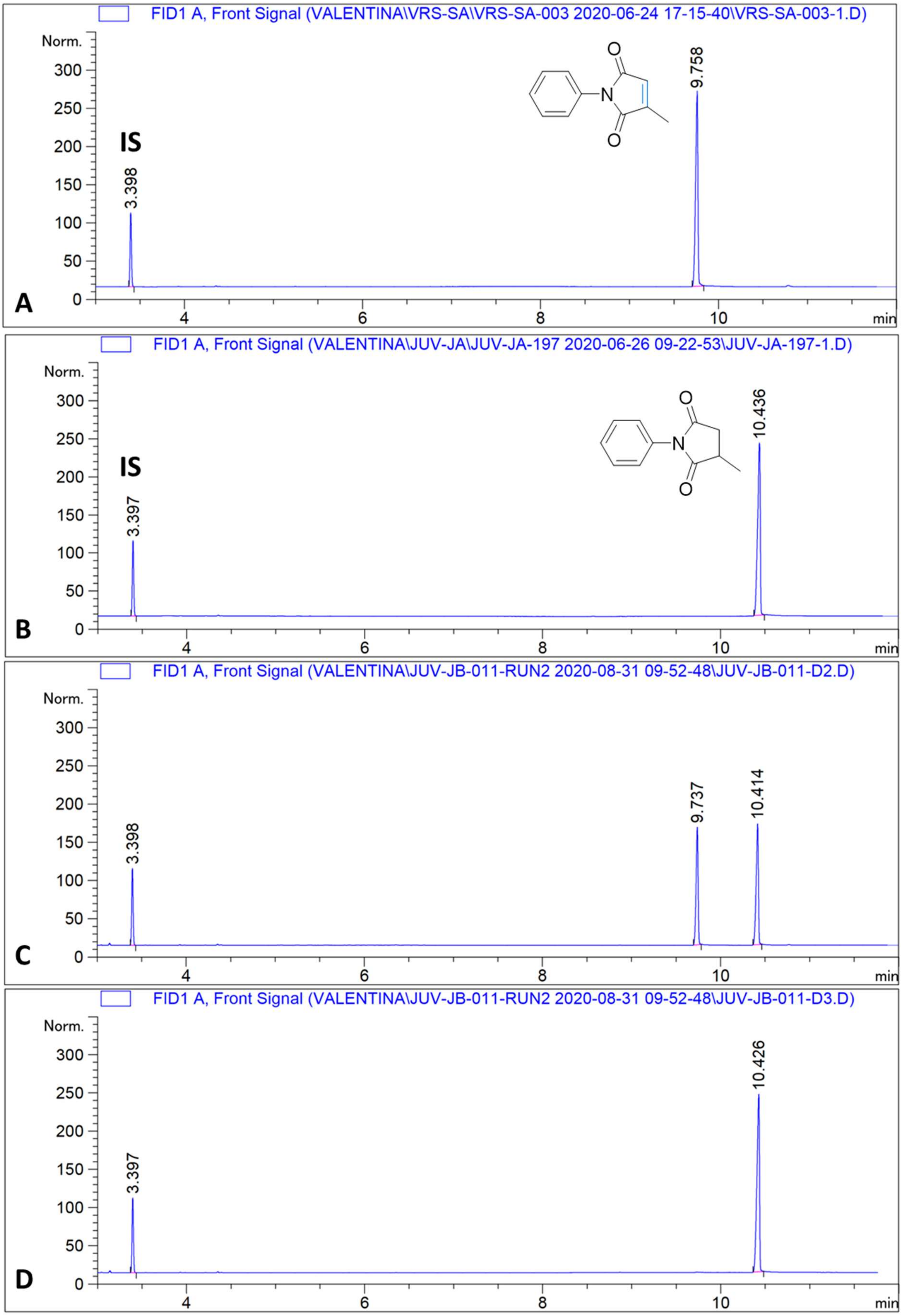

Figure S8. Representative GC-FID chromatograms for quantification of **2a** and **2b**. **(A)** reference compound **2a**; **(B)** reference compound **2b**; **(C)** illuminated reduction of **2a** using the MPS at 90 µE m^{-2} s^{-1} after 1h and **(D)** after 3h.

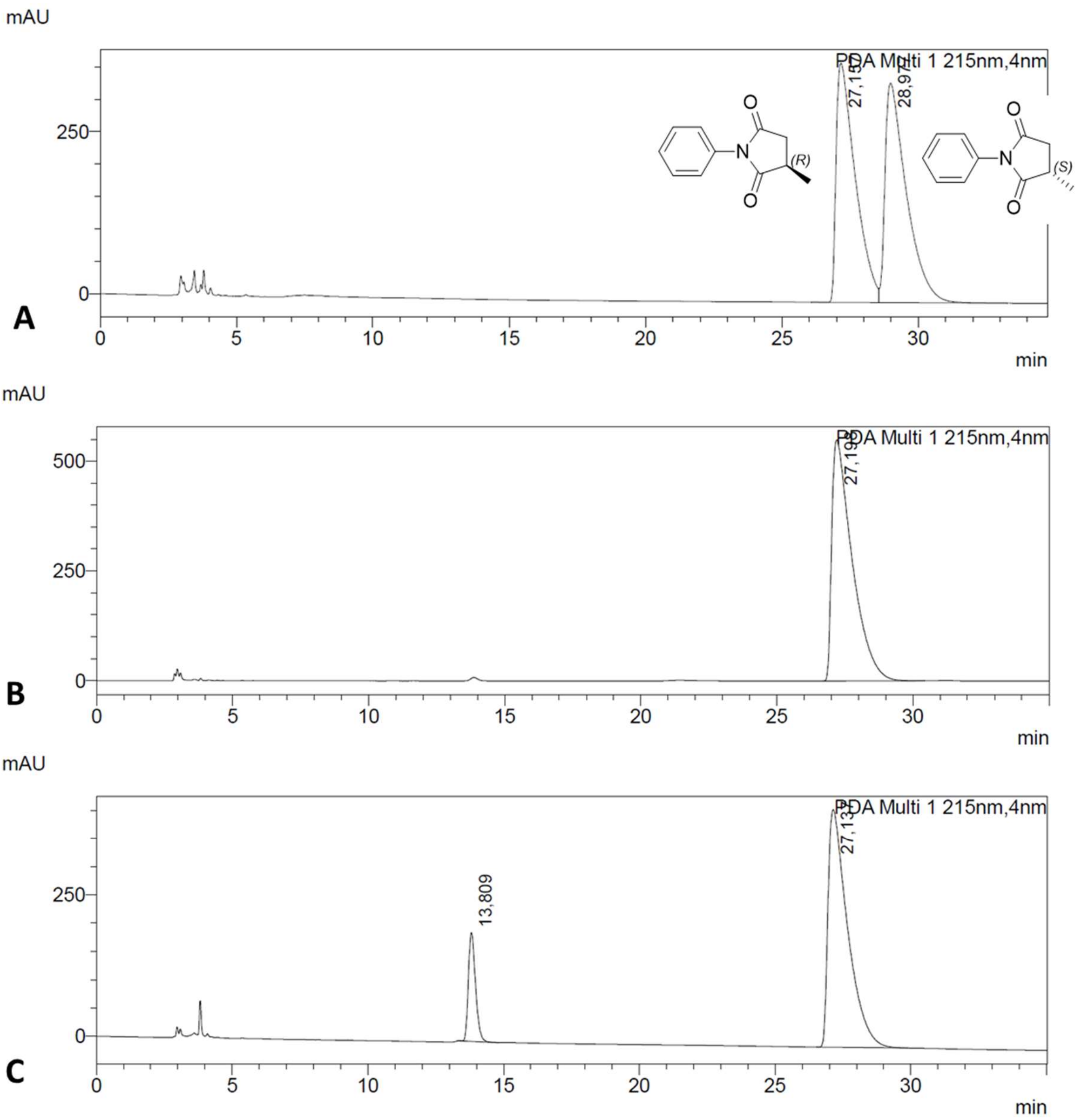

Figure S9. Representative HPLC chromatograms for determining the enantiomeric excess of **2b**. **(A)** Racemic reference compound; **(B)** illuminated reduction of **2a** to **2b** using the MPS at analytical scale and **(C)** at 50 mL scale.

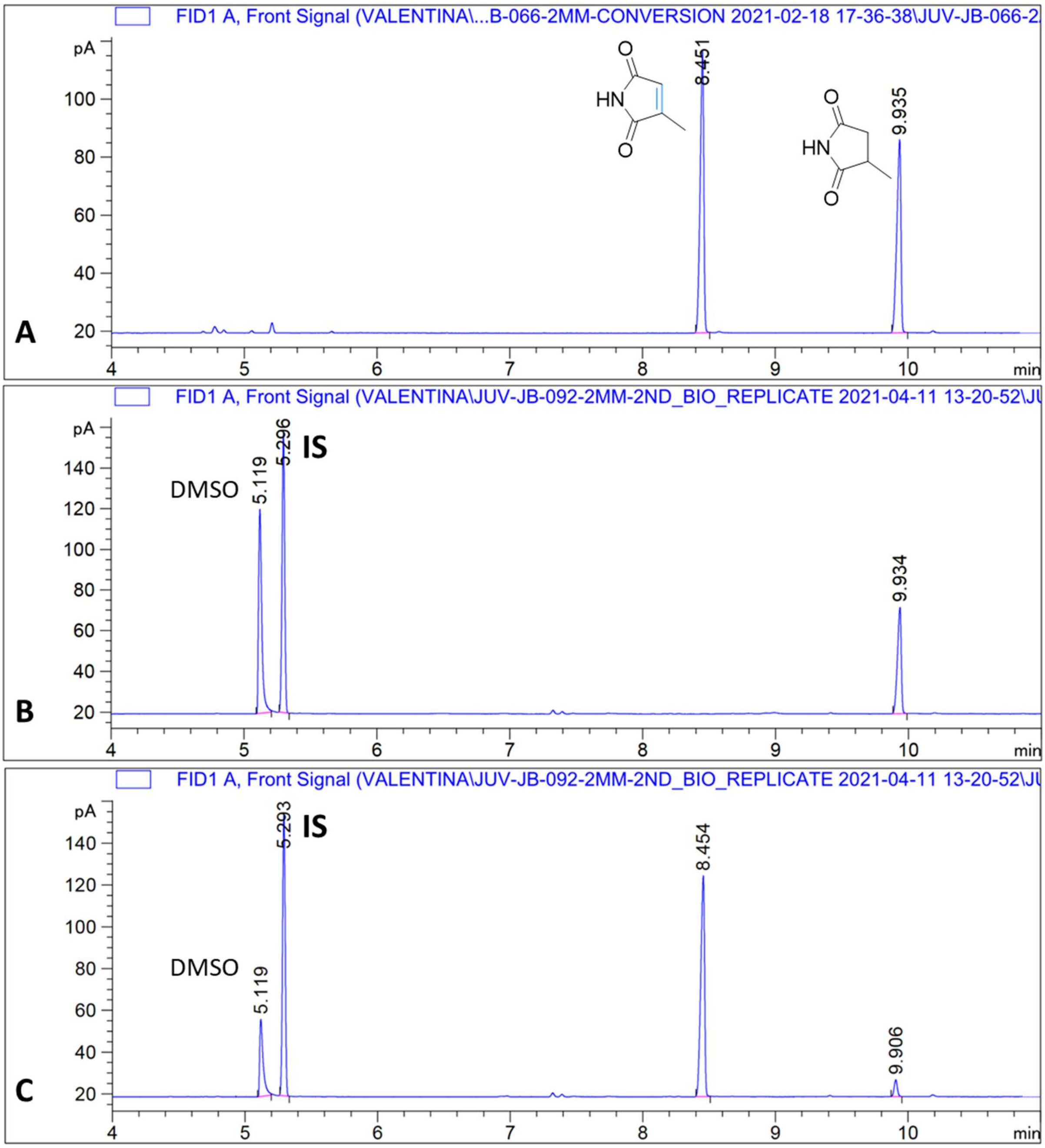

Figure S10. Representative GC-FID chromatograms for quantification of **3a** and **3b**. **(A)** Mixture of reference compounds **3a** and **3b**; **(B)** illuminated and **(C)** dark reduction of **3a** using the MPS.

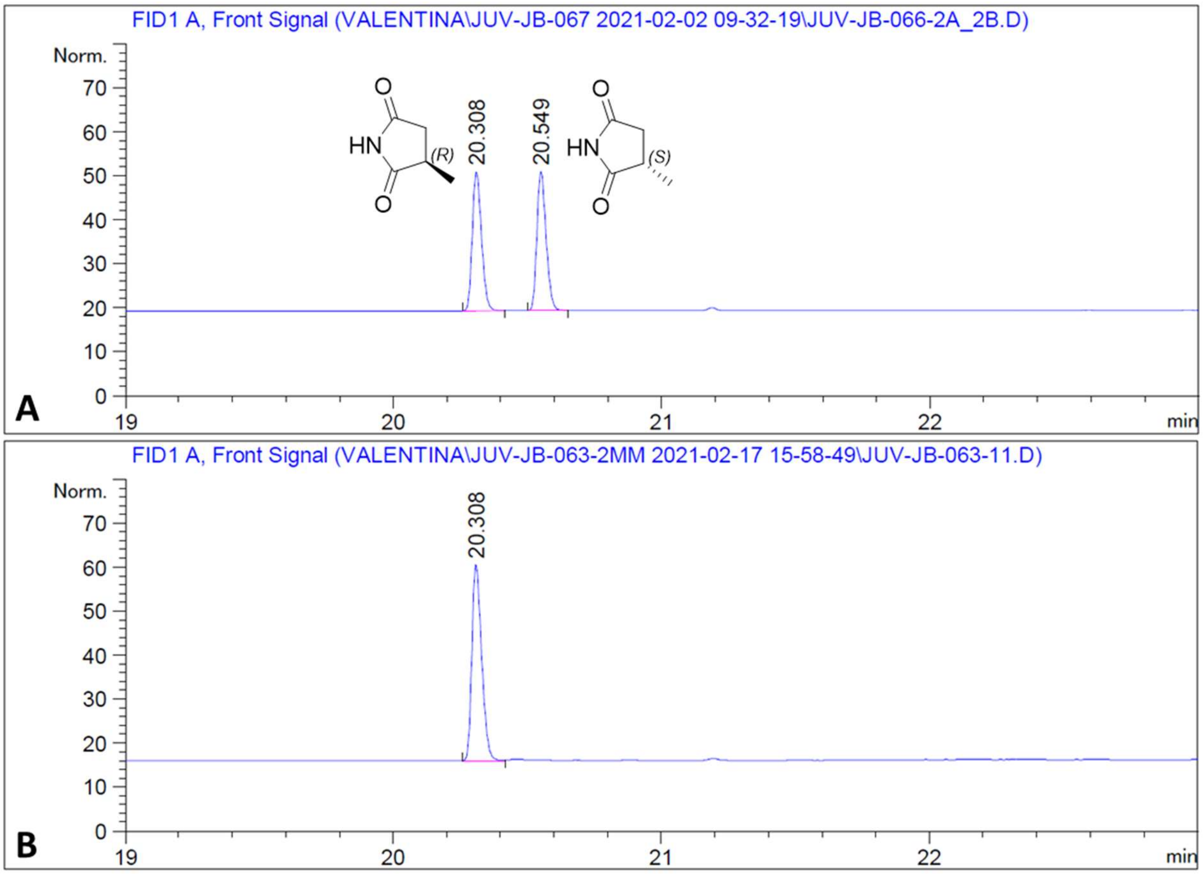

Figure S11. Representative GC-FID chromatograms for determining the enantiomeric excess of **3b**. **(A)** Racemic reference compound; **(B)** illuminated reduction of **3a** to **3b** using the MPS.

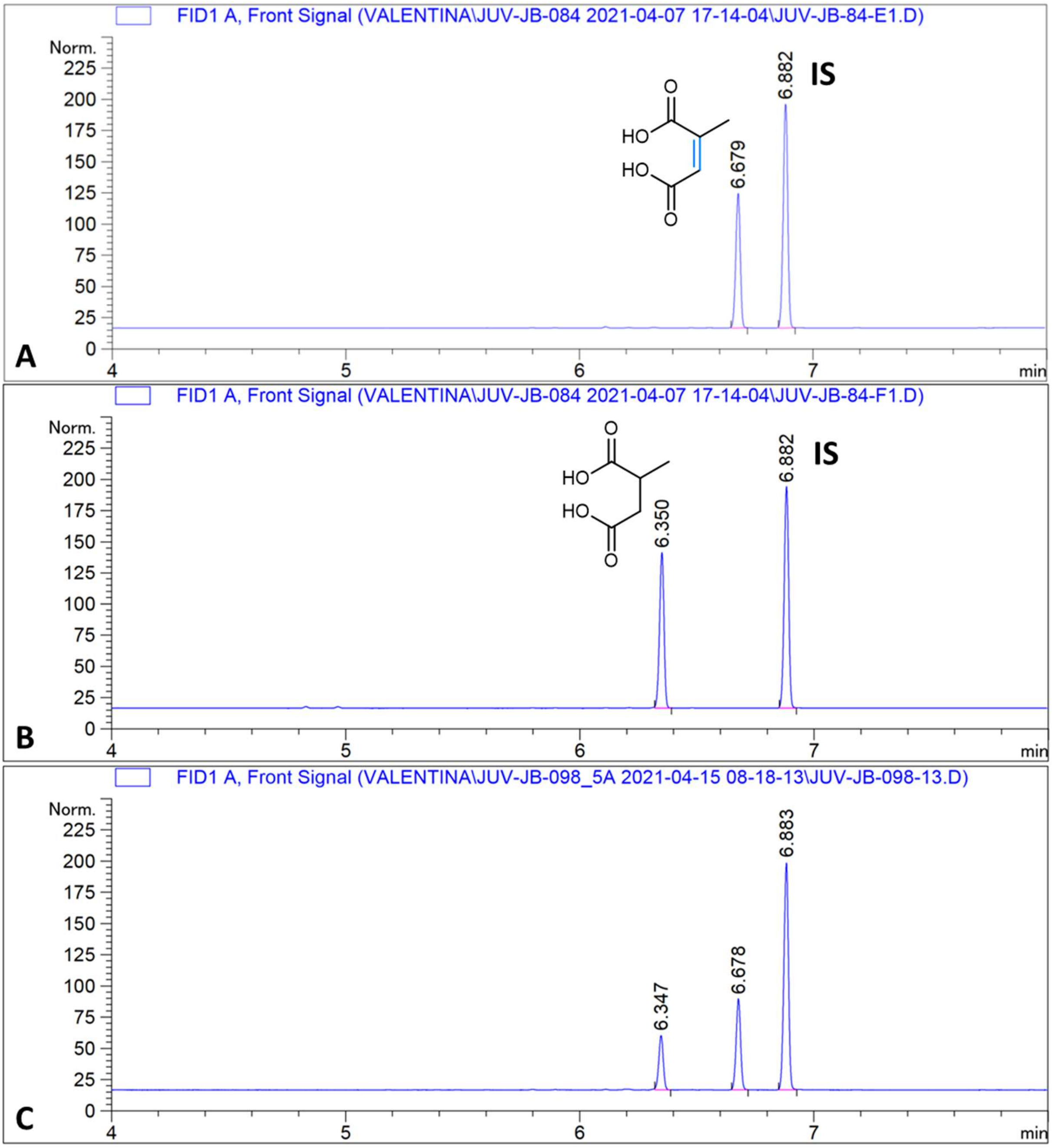

Figure S12. Representative GC-FID chromatograms for quantification of **3a** and **3b**, derivatized as trimethylsilyl diester. **(A)** reference compound **4a**; **(B)** reference compound **4b**; **(C)** illuminated reduction of **4a** using the MPS.

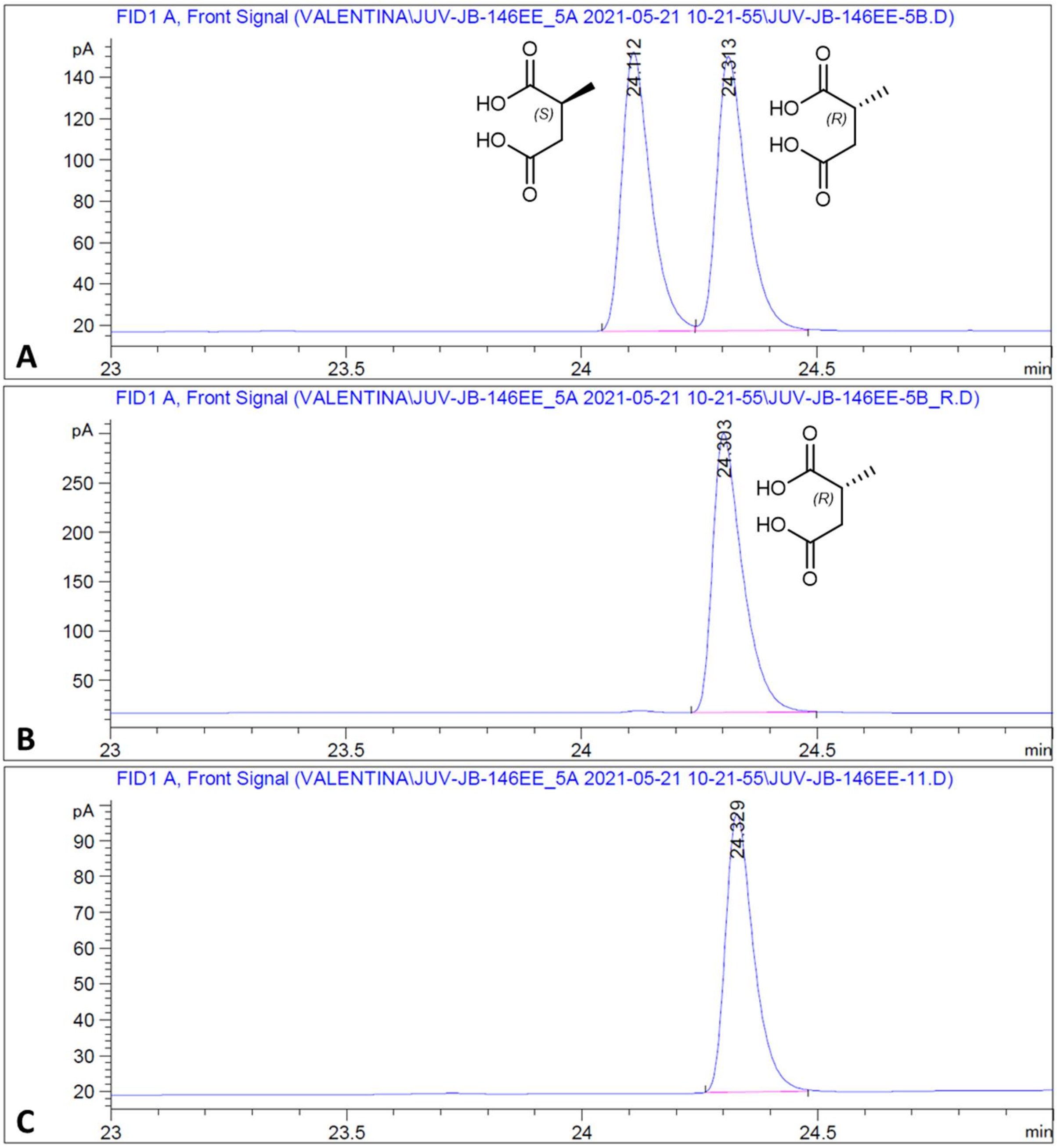

Figure S13. Representative GC-FID chromatograms for determining the enantiomeric excess of **4b**, derivatized as dimethyl ester. **(A)** Racemic reference compound; **(B)** enantiopure (*R*)-**4b** reference; **(C)** illuminated reduction of **4a** to **4b** using the MPS.

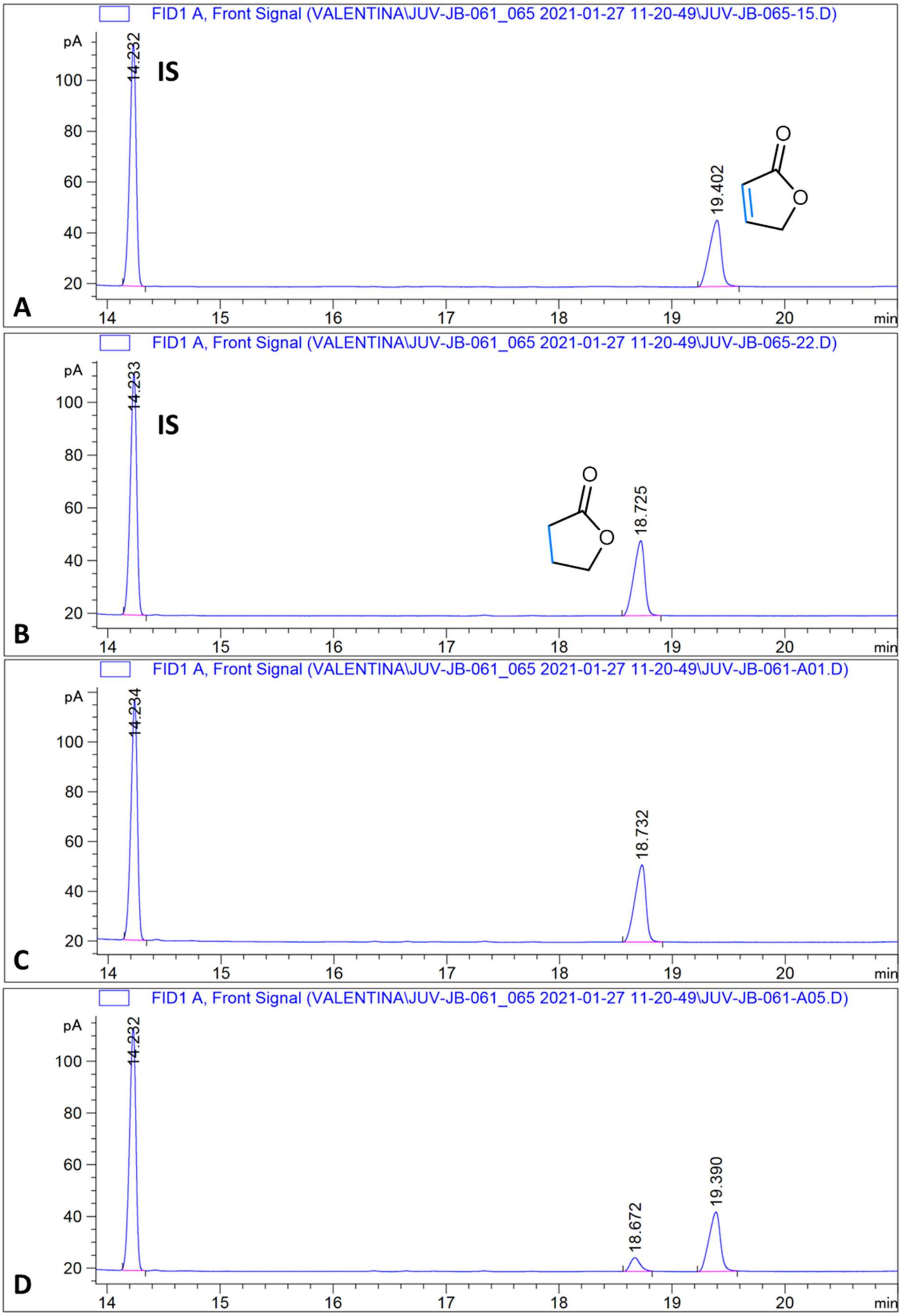

Figure S14. Representative GC-FID chromatograms for quantification of **5a** and **5b**. **(A)** reference compound **5a**; **(B)** reference compound **5b**; **(C)** illuminated and **(D)** dark reduction of **5a** using the MPS.

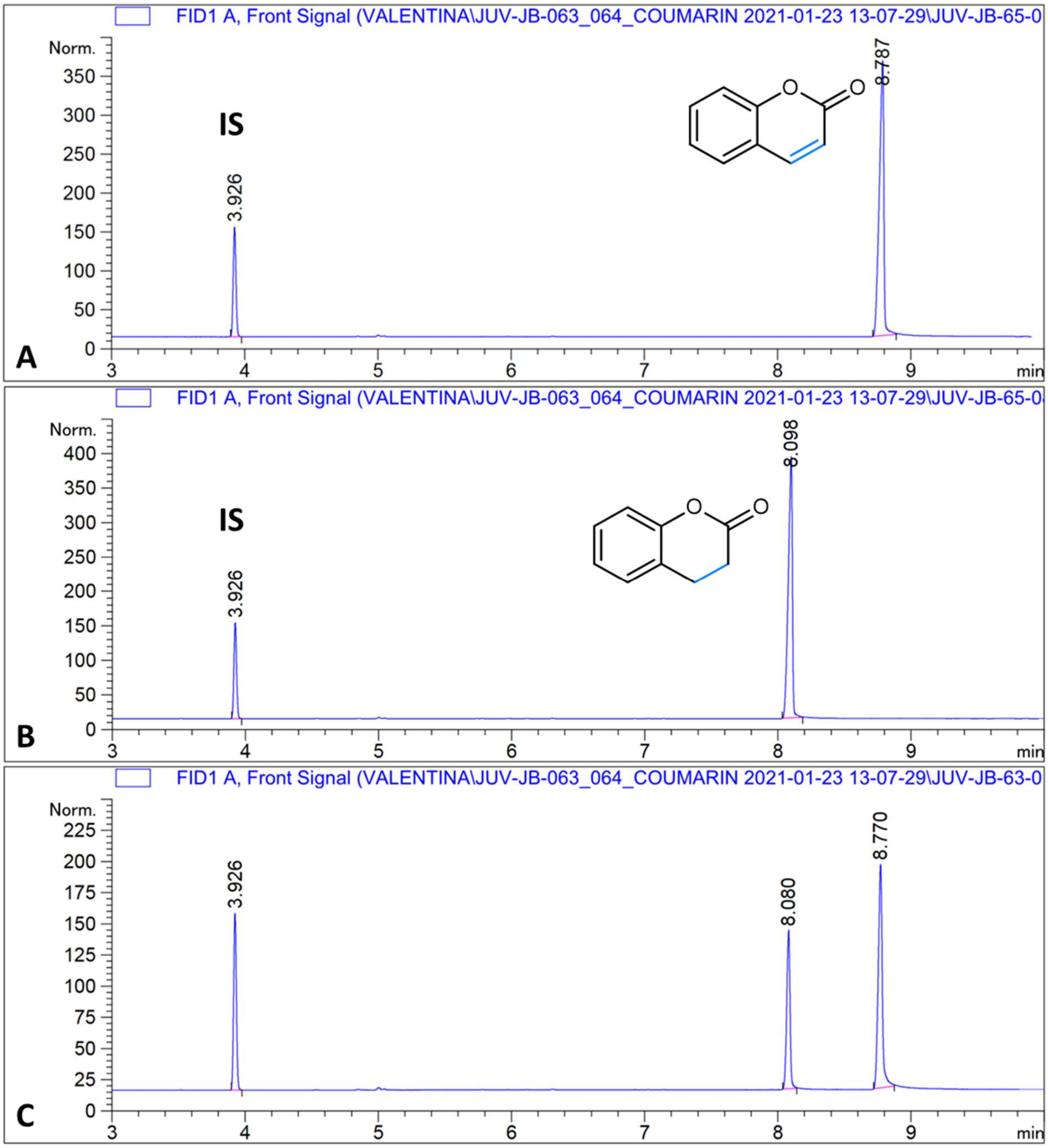

Figure S15. Representative GC-FID chromatograms for quantification of **6a** and **6b**. **(A)** reference compound **6a**; **(B)** reference compound **6b**; **(C)** illuminated reduction of **6a** using the MPS.

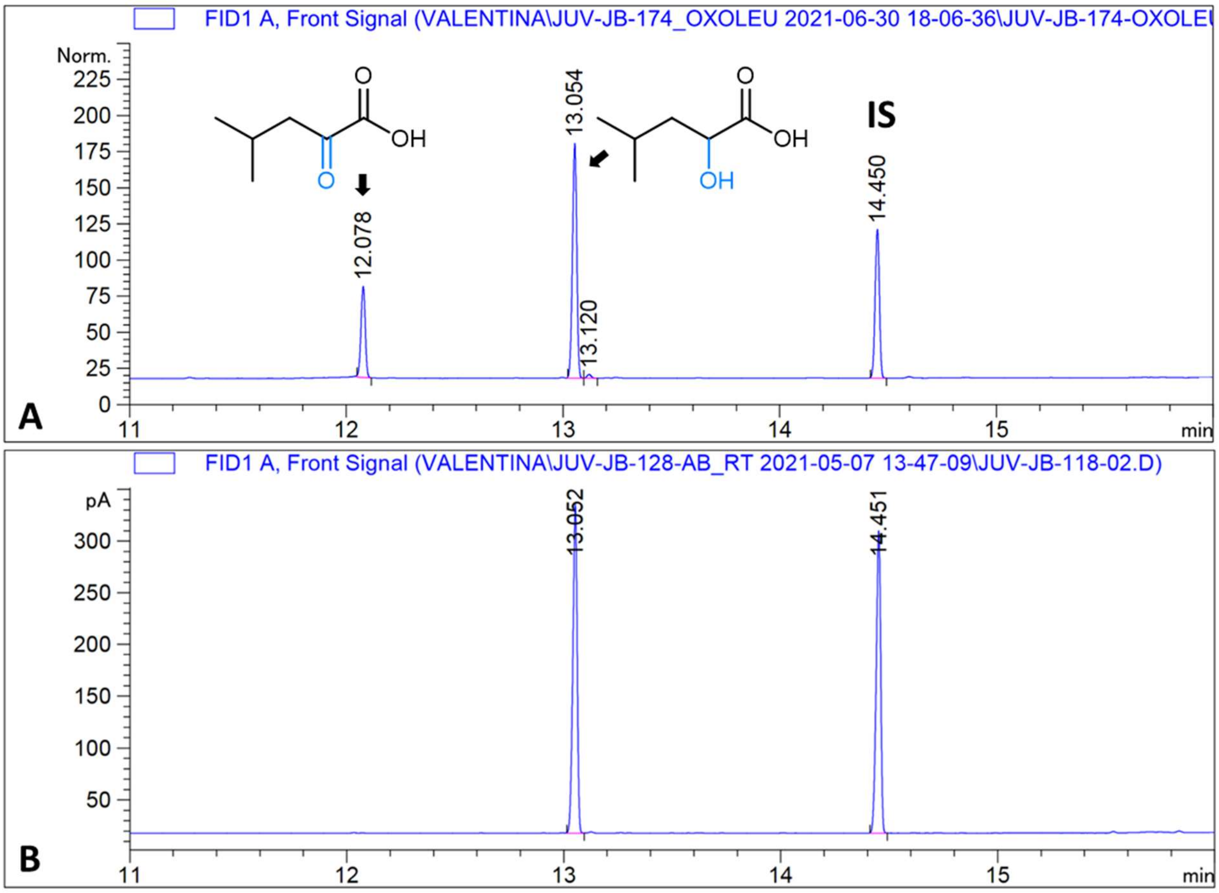

Figure S16. Representative GC-FID chromatograms for quantification of **7a** and **7b**, derivatized as trimethylsilyl esters. **(A)** Mixture of reference compounds **7a** and **7b**; **(B)** illuminated reduction of **7a** using the MPS.

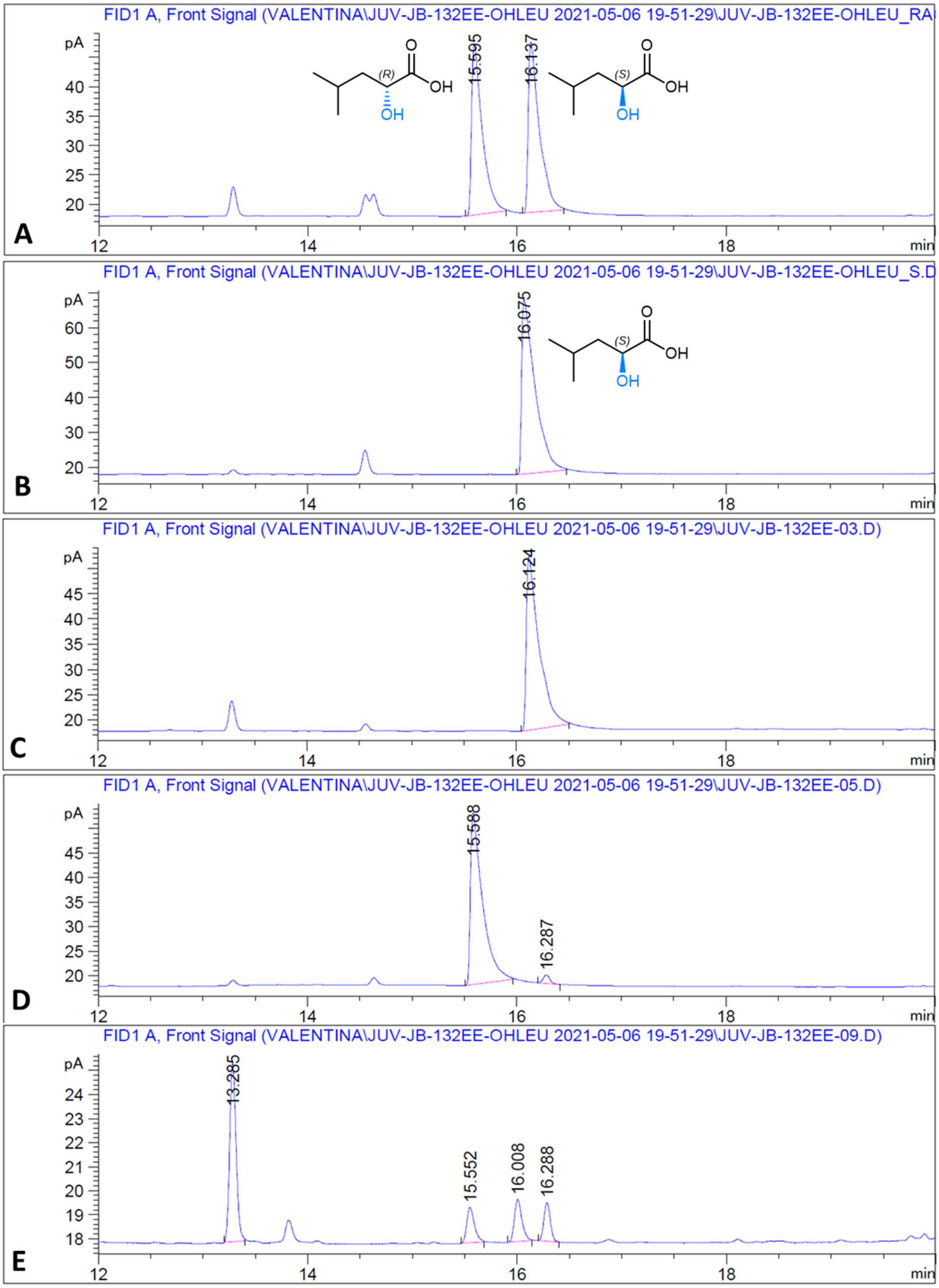

Figure S17. Representative GC-FID chromatograms for determining the enantiomeric excess of **7b**, derivatized as methyl ester. **(A)** Racemic reference compound; **(B)** enantiopure (*S*)-**7b** reference; **(C)** illuminated reduction of **7a** to (*S*)-**7b** with L-HicDH, or **(D)** to (*R*)-**7b** with D-HicDH, both using the MPS; **(E)** reaction sample with no substrate.

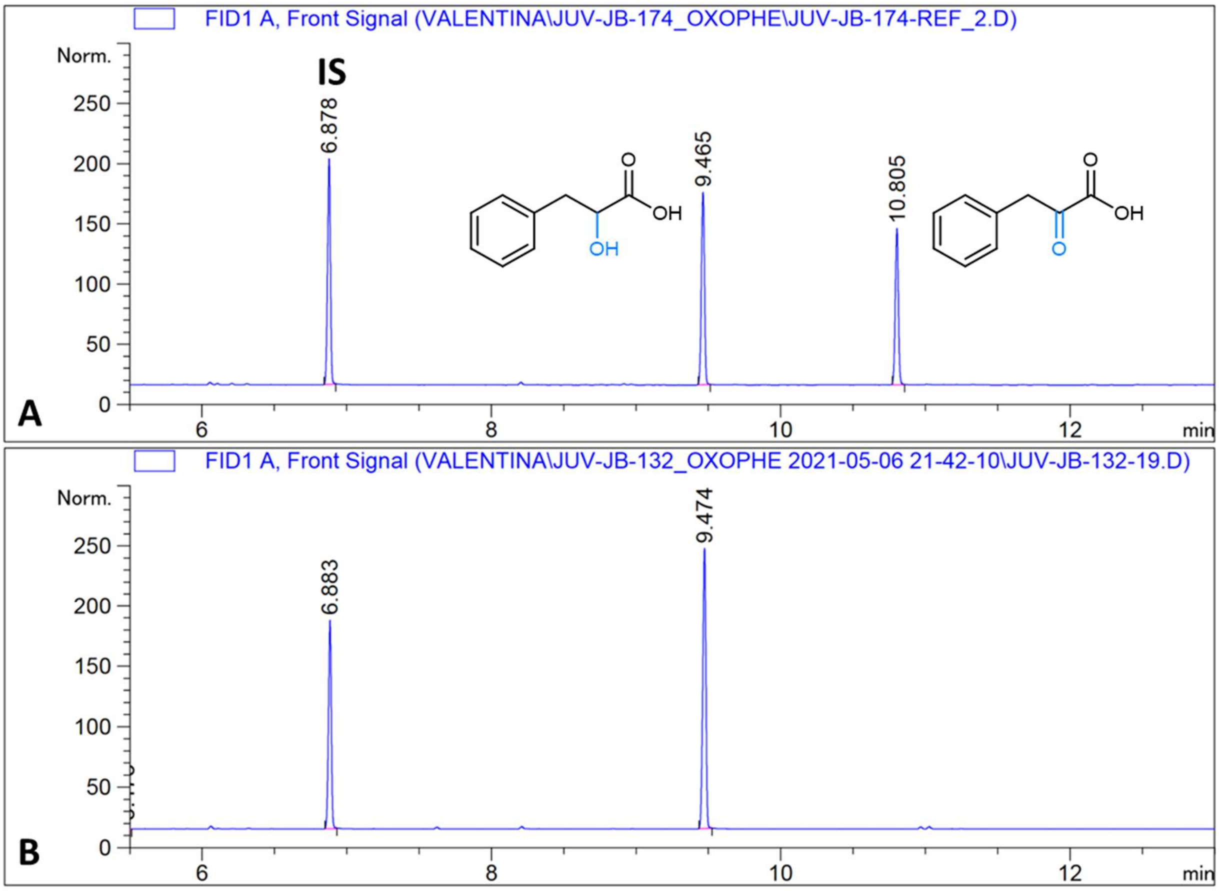

Figure S18. Representative GC-FID chromatograms for quantification of **8a** and **8b**, derivatized as trimethylsilyl esters. **(A)** Mixture of reference compounds **8a** and **8b**; **(B)** illuminated reduction of **8a** using the MPS.

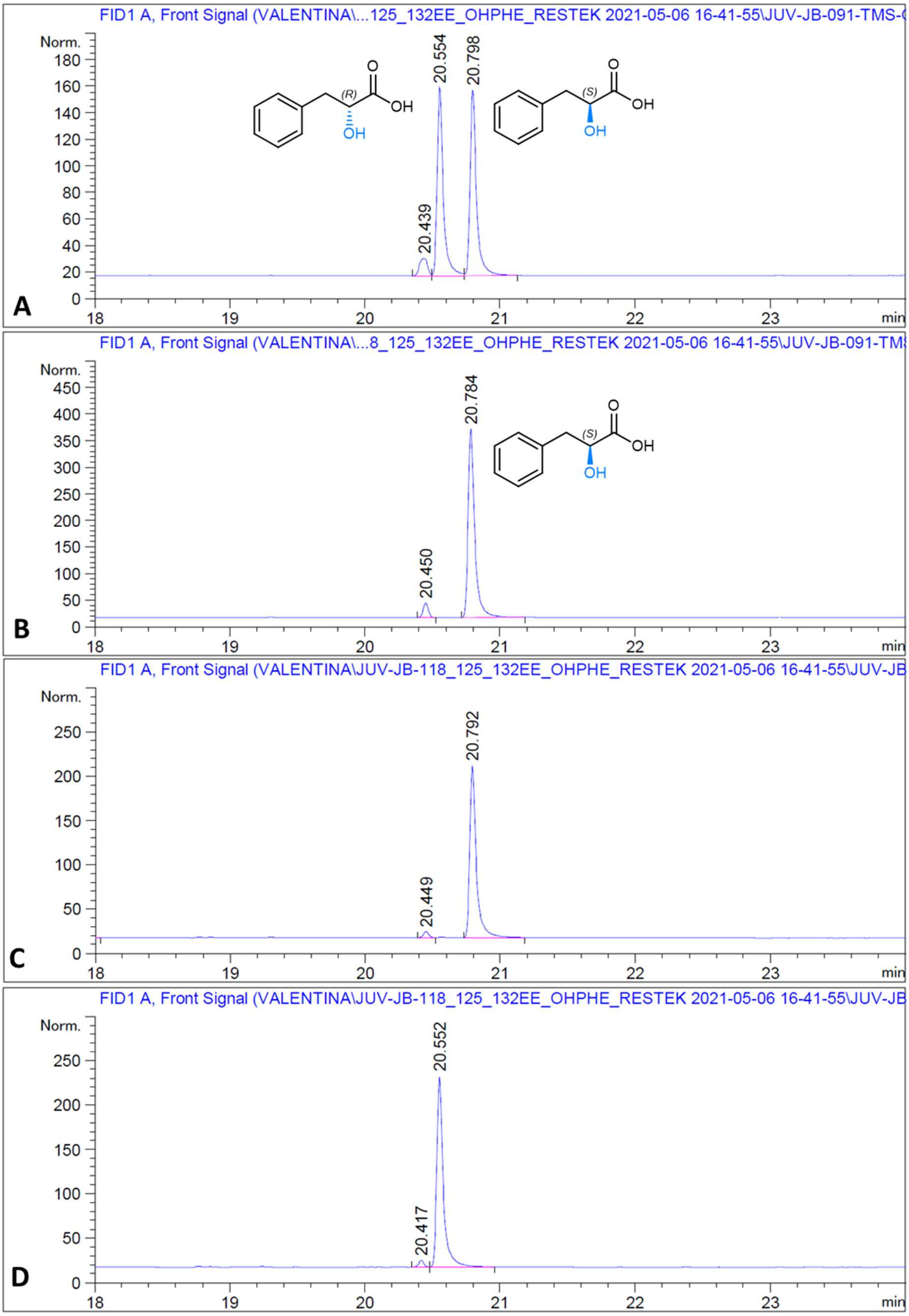

Figure S19. Representative GC-FID chromatograms for determining the enantiomeric excess of **8b**, derivatized as methyl ester. **(A)** Racemic reference compound; **(B)** enantiopure (*S*)-**8b** reference; **(C)** illuminated reduction of **8a** to (*S*)-**8b** with L-HicDH, or **(D)** to (*R*)-**8b** with D-HicDH, both using the MPS.

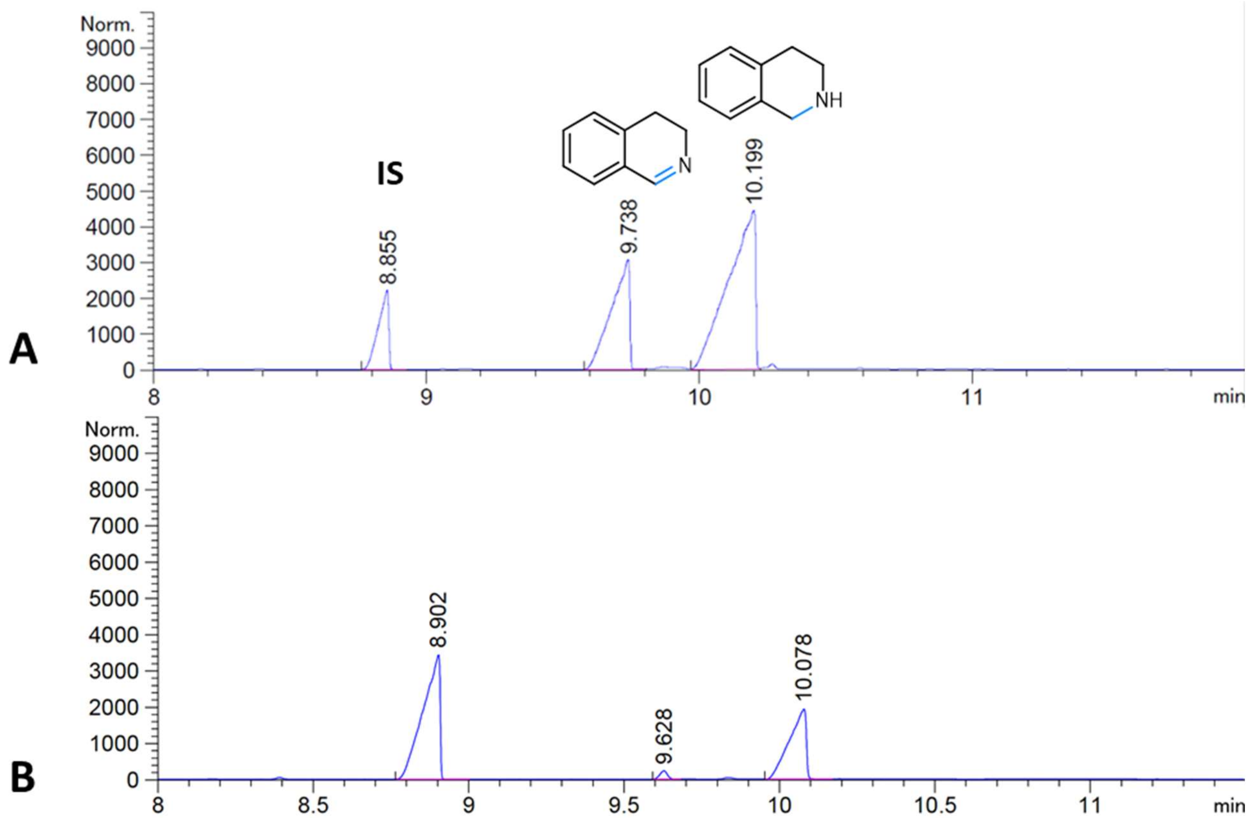

Figure S20. Representative GC-FID chromatograms for quantification of **9a** and **9b**. **(A)** Mixture of reference compounds **9a** and **9b**; **(B)** illuminated reduction of **9a** using the MPS with IRED A.

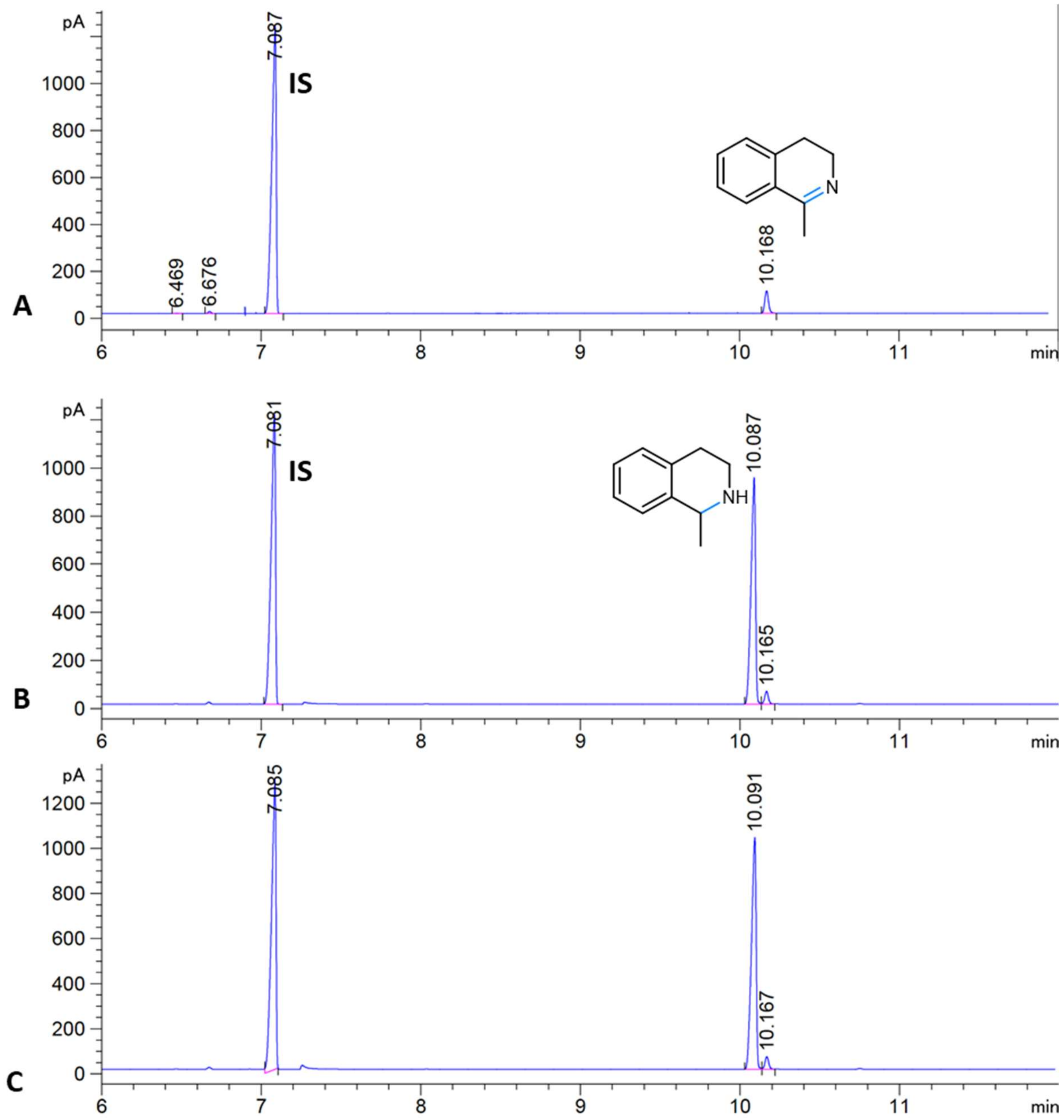

Figure S21. Representative GC-FID chromatograms for quantification of **10a** and **10b**. **(A)** reference compound **10a**; **(B)** reference compound **10b**; **(C)** illuminated reduction of **10a** using the MPS with IRED A.

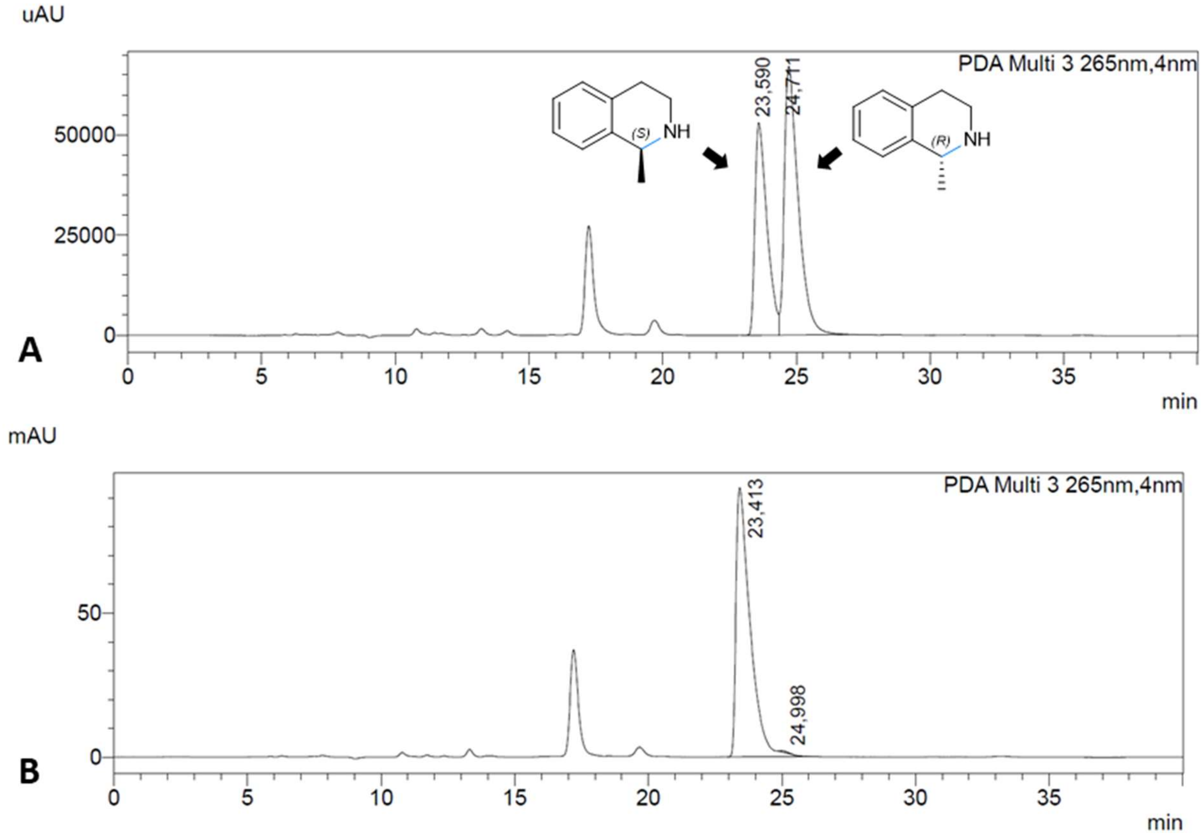

Figure S22. Representative HPLC chromatograms for determining the enantiomeric excess of **10b**. **(A)** illuminated (unselective) reduction of **10a** with IRED A, and **(B)** illuminated (*S*)-selective reduction of **10a** with IRED J, both using the MPS.

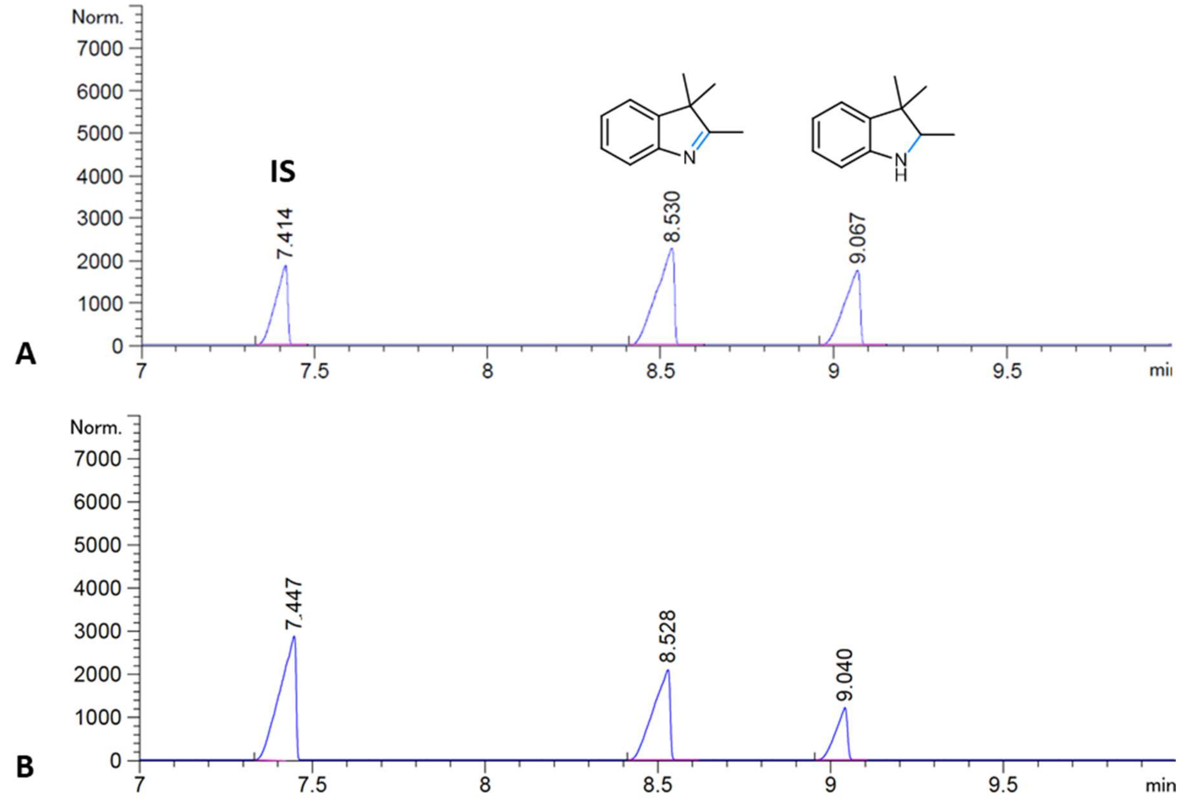

Figure S23. Representative GC-FID chromatograms for quantification of **11a** and **11b**. **(A)** Mixture of reference compounds **11a** and **11b**; **(B)** illuminated reduction of **11a** using the MPS with IRED A.

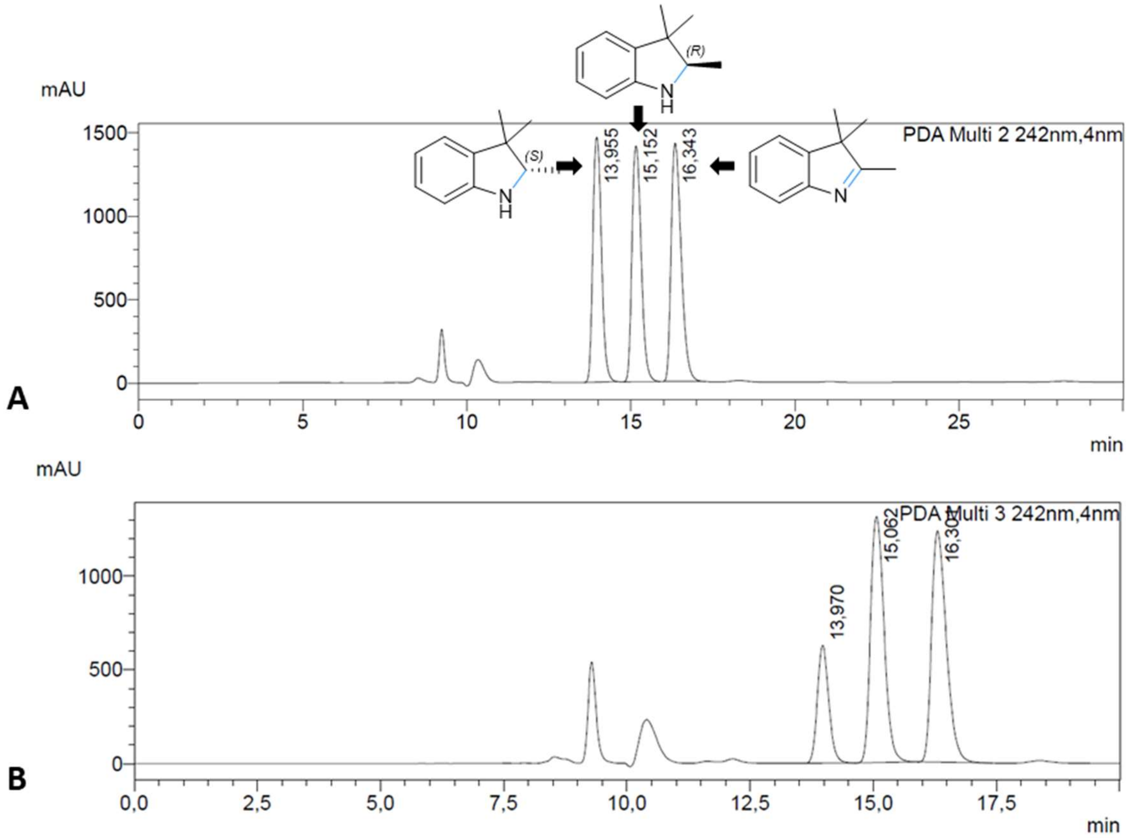

Figure S24. Representative HPLC chromatograms for determining the enantiomeric excess of **11b**. **(A)** Mixture of reference compounds *rac*-**11b** and **11a**; **(B)** illuminated reduction of **11a** with IRED A using the MPS.

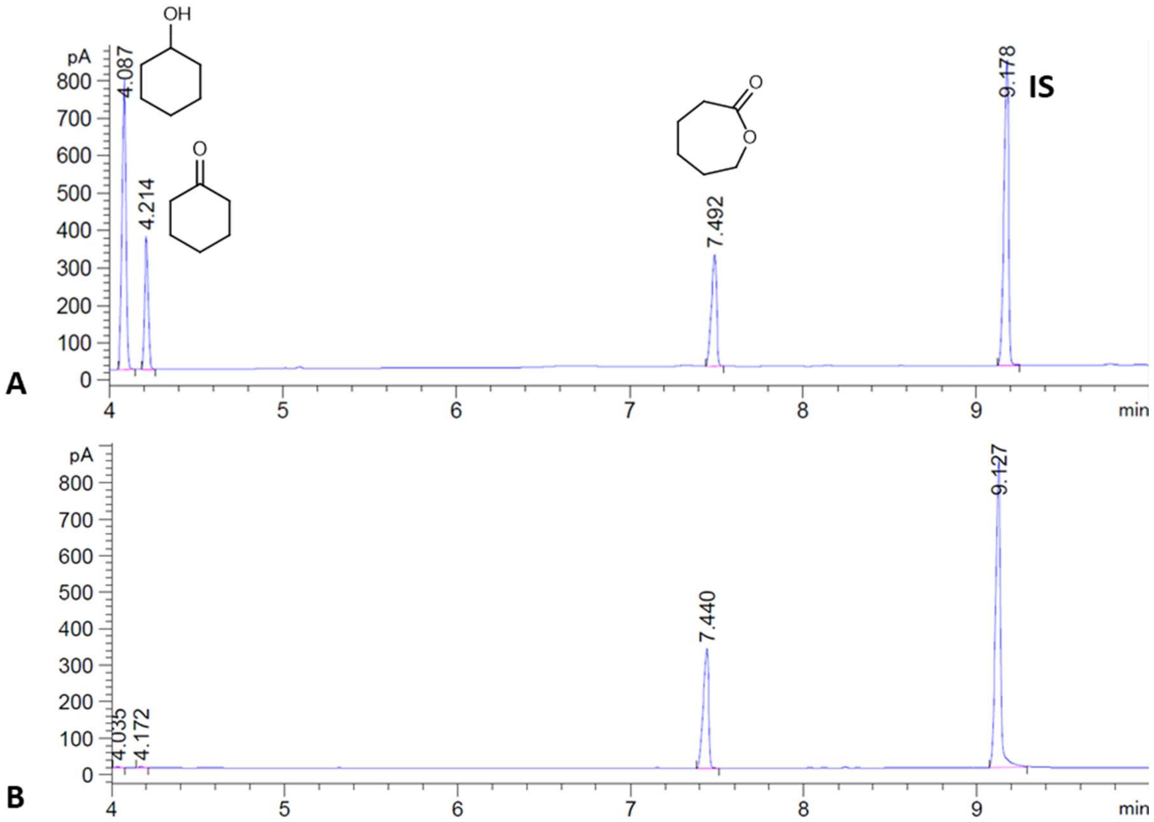

Figure S25. Representative GC-FID chromatograms for quantification of **12a**, **12b** and **12c**. **(A)** Mixture of reference compounds **12a**, **12b** and **12c**; **(B)** illuminated monooxygenation of **12a** using the MPS.

4.3 Calibration curves

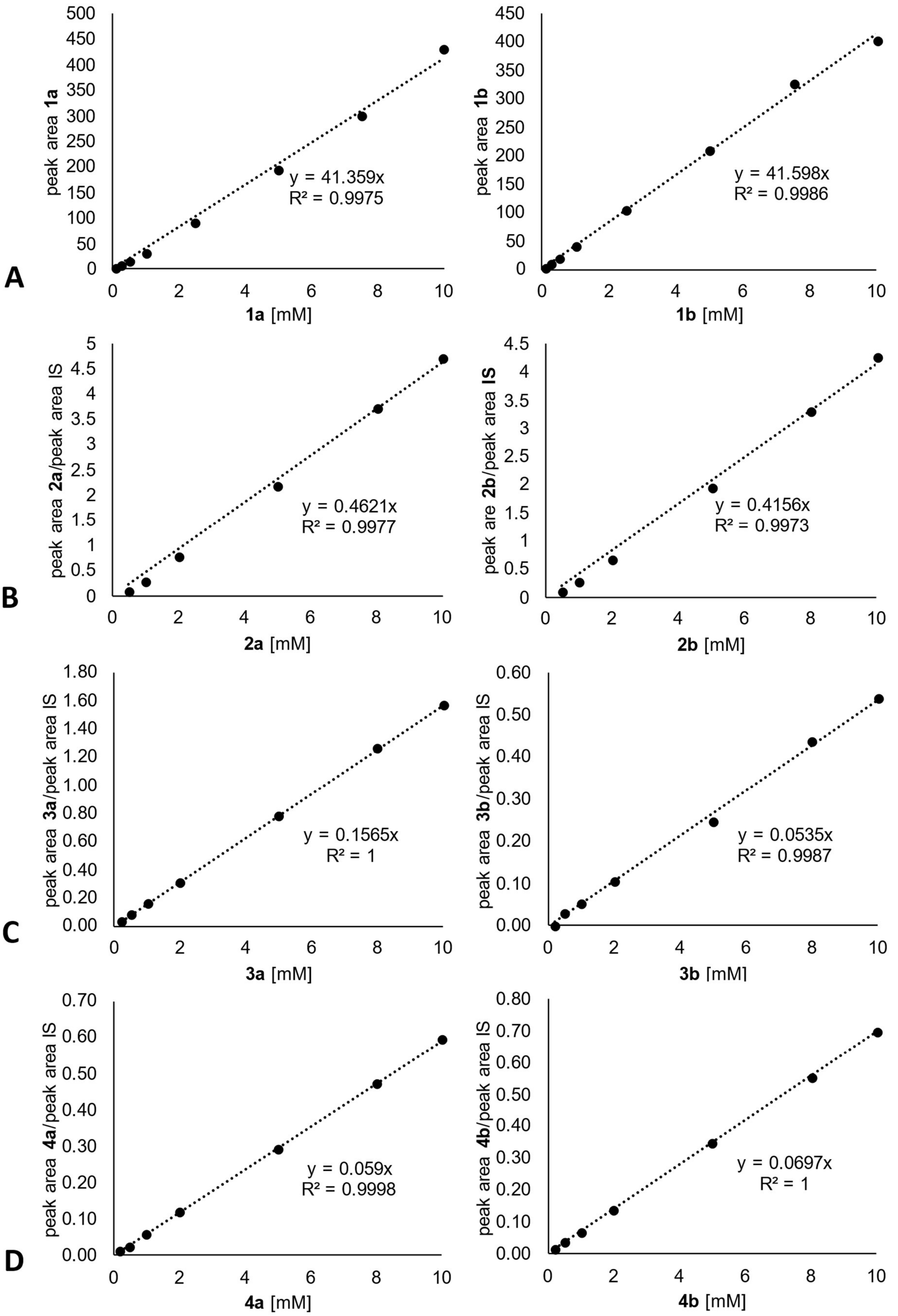

Figure S26. Calibration curves for the quantification of **(A) 1a/1b**, **(B) 2a/2b**, **(C) 3a/3b** and **(D) 4a/4b**. The corresponding line equations and R^2 values were generated by a simple linear regression and were forced through zero.

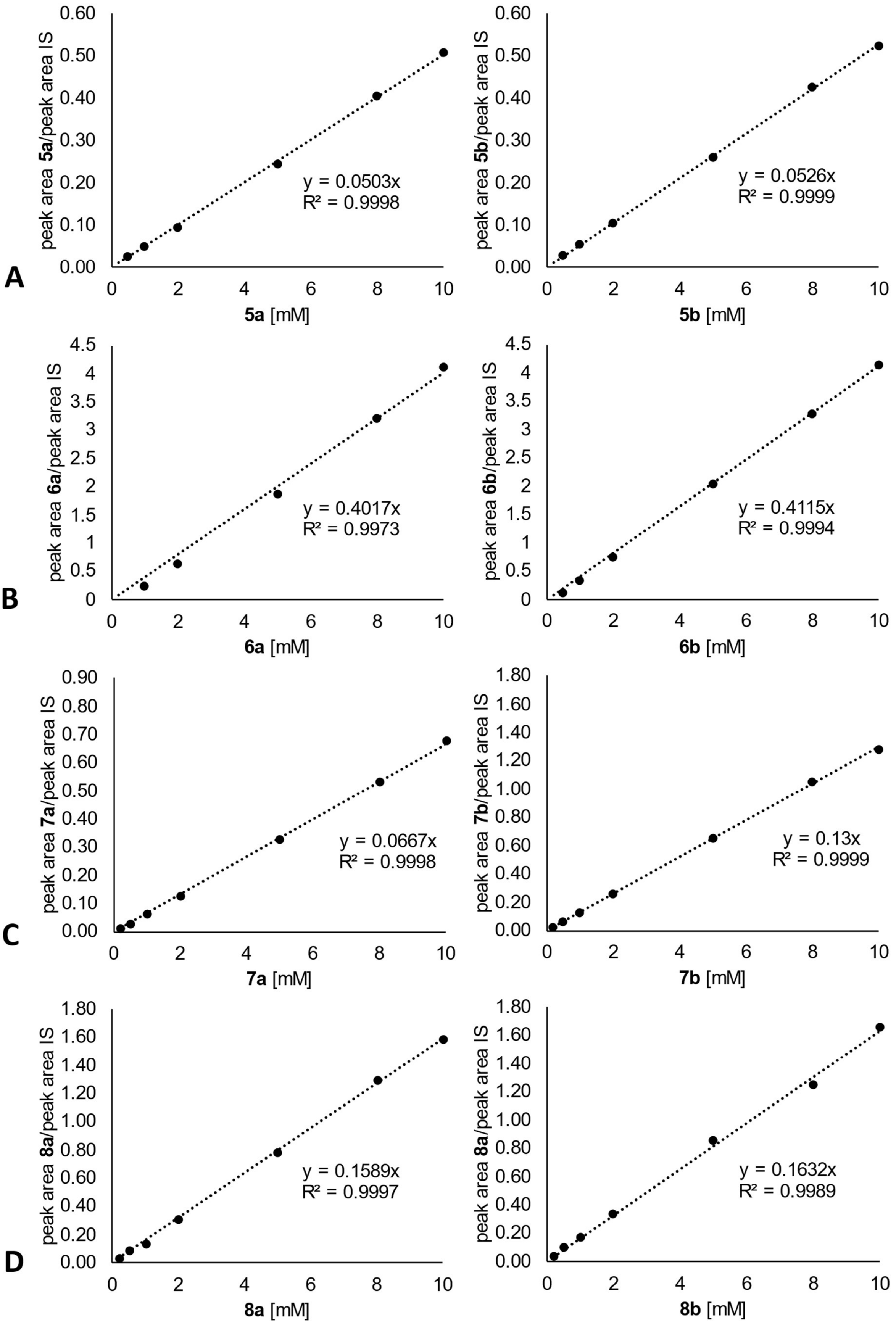

Figure S27. Calibration curves for the quantification of **(A) 5a**/**5b**, **(B) 6a**/**6b**, **(C) 7a**/**7b** and **(D) 8a**/**8b**. The corresponding line equations and R^2 values were generated by a simple linear regression and were forced through zero.

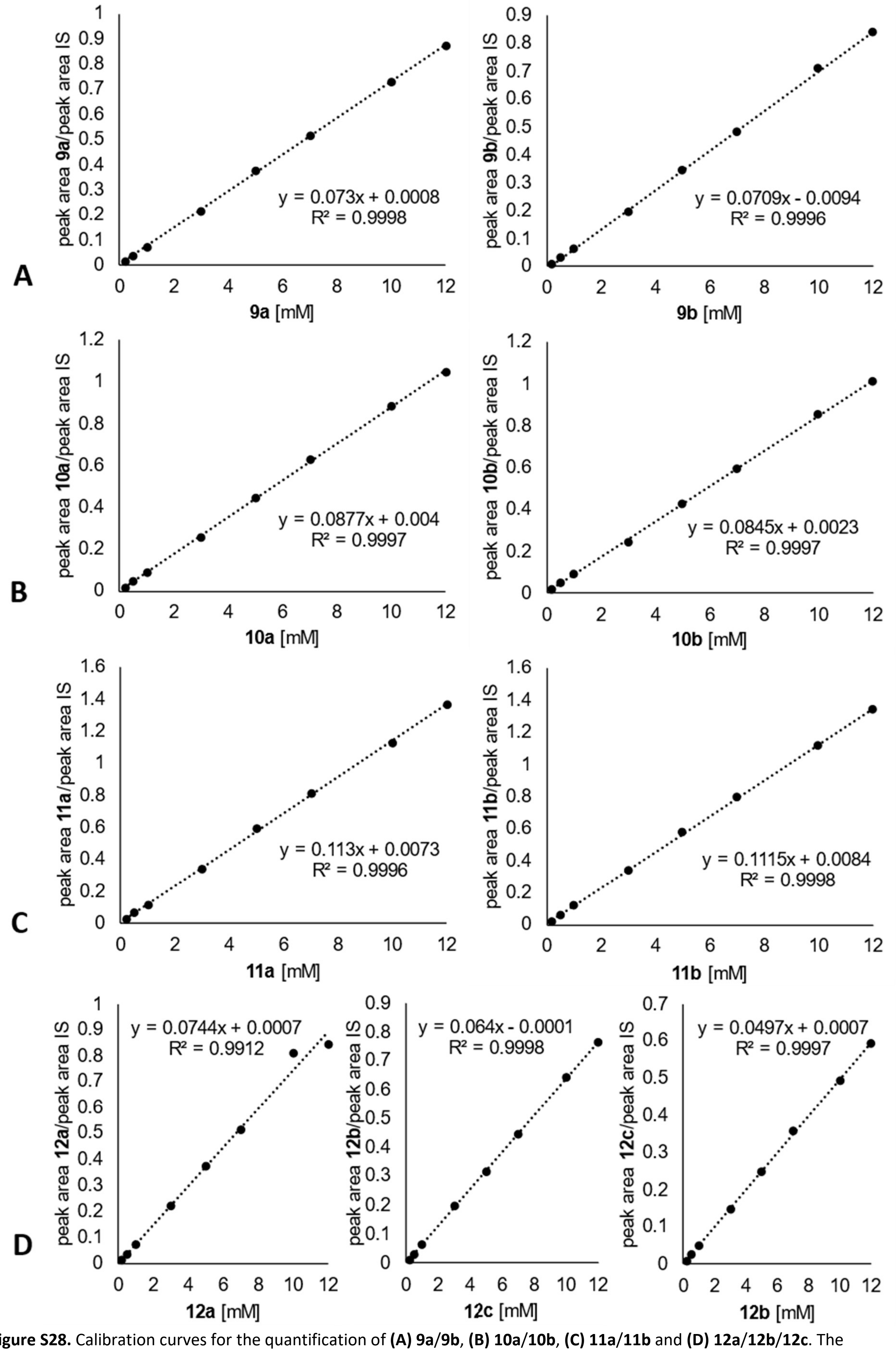

Figure S28. Calibration curves for the quantification of **(A) 9a**/**9b**, **(B) 10a**/**10b**, **(C) 11a**/**11b** and **(D) 12a**/**12b**/**12c**. The corresponding line equations and R^2 values were generated by a simple linear regression and were not forced through zero.

5 References

[1] R. Y. Stanier, R. Kunisawa, M. Mandel, G. Cohen-Bazire, *Bacteriol. Rev.* **1971**, *35*, 171-205.

[2] Y. Kanesaki, Y. Shiwa, N. Tajima, M. Suzuki, S. Watanabe, N. Sato, M. Ikeuchi, H. Yoshikawa, *DNA Res.* **2012**, *19*, 67-79.

[3] D. Trautmann, B. Voss, A. Wilde, S. Al-Babili, W. R. Hess, *DNA Res.* **2012**, *19*, 435-448.

[4] A. Sengupta, A. V. Sunder, S. V. Sohoni, P. P. Wangikar, *J. Biotechnol.* **2019**, *289*, 1-6.

[5] A. Weckbecker, W. Hummel, *Biocatal. Biotransform.* **2009**, *24*, 380-389.

[6] B. Kosjek, W. Stampfer, M. Pogorevc, W. Goessler, K. Faber, W. Kroutil, *Biotechnol. Bioeng.* **2004**, *86*, 55-62.

[7] K. Edegger, C. C. Gruber, T. M. Poessl, S. R. Wallner, I. Lavandera, K. Faber, F. Niehaus, J. Eck, R. Oehrlein, A. Hafner, W. Kroutil, *Chem. Commun.* **2006**, 2402-2404.

[8] K. Tauber, M. Hall, W. Kroutil, W. M. Fabian, K. Faber, S. M. Glueck, *Biotechnol. Bioeng.* **2011**, *108*, 1462-1467.

[9] X. Q. Pei, M. Y. Xu, Z. L. Wu, *J. Mol. Catal. B: Enzym.* **2016**, *123*, 91-99.

[10] S. Litthauer, S. Gargiulo, E. van Heerden, F. Hollmann, D. J. Opperman, *J. Mol. Catal. B: Enzym.* **2014**, *99*, 89-95.

[11] M. Hall, C. Stueckler, B. Hauer, R. Stuermer, T. Friedrich, M. Breuer, W. Kroutil, K. Faber, *Eur. J. Org. Chem.* **2008**, *2008*, 1511-1516.

[12] J. R. Snape, N. A. Walkley, A. P. Morby, S. Nicklin, G. F. White, *J. Bacteriol.* **1997**, *179*, 7796-7802.

[13] M. Hall, C. Stueckler, W. Kroutil, P. Macheroux, K. Faber, *Angew. Chem., Int. Ed.* **2007**, *46*, 3934-3937.

[14] M. Hall, C. Stueckler, H. Ehammer, E. Pointner, G. Oberdorfer, K. Gruber, B. Hauer, R. Stuermer, W. Kroutil, P. Macheroux, K. Faber, *Adv. Synth. Catal.* **2008**, *350*, 411-418.

[15] N. G. Turrini, M. Hall, K. Faber, *Adv. Synth. Catal.* **2015**, *357*, 1861-1871.

[16] P. van Dillewijn, R. M. Wittich, A. Caballero, J. L. Ramos, *Appl. Environ. Microbiol.* **2008**, *74*, 6703-6708.

[17] P. van Dillewijn, R. M. Wittich, A. Caballero, J. L. Ramos, *Appl. Environ. Microbiol.* **2008**, *74*, 6820-6823.

[18] D. J. Opperman, L. A. Piater, E. van Heerden, *J. Bacteriol.* **2008**, *190*, 3076-3082.

[19] D. S. Blehert, B. G. Fox, G. H. Chambliss, *J. Bacteriol.* **1999**, *181*, 6254-6263.

[20] X. Q. Sheng, M. Yan, L. Xu, M. Wei, *J. Mol. Catal. B: Enzym.* **2016**, *130*, 18-24.

[21] K. Kitzing, T. B. Fitzpatrick, C. Wilken, J. Sawa, G. P. Bourenkov, P. Macheroux, T. Clausen, *J. Biol. Chem.* **2005**, *280*, 27904-27913.

[22] T. B. Fitzpatrick, N. Amrhein, P. Macheroux, *J. Biol. Chem.* **2003**, *278*, 19891-19897.

[23] X. Gao, J. Ren, Q. Wu, D. Zhu, *Enzyme Microb. Technol.* **2012**, *51*, 26-34.

[24] S. Velikogne, V. Resch, C. Dertnig, J. H. Schrittwieser, W. Kroutil, *ChemCatChem* **2018**, *10*, 3236-3246.

[25] H. C. Büchsenschütz, V. Vidimce-Risteski, B. Eggbauer, S. Schmidt, C. K. Winkler, J. H. Schrittwieser, W. Kroutil, R. Kourist, *ChemCatChem* **2019**, *12*, 726-730.

[26] H. Schutte, W. Hummel, M. R. Kula, *Appl. Microbiol. Biotechnol.* **1984**, *19*, 167-176.

[27] G. Gourinchas, E. Busto, M. Killinger, N. Richter, B. Wiltschi, W. Kroutil, *Chem. Commun.* **2015**, *51*, 2828-2831.

[28] W. Hummel, H. Schutte, M. R. Kula, *Appl. Microbiol. Biotechnol.* **1985**, *21*, 7-15.

[29] S. Böhmer, K. Köninger, Á. Gómez-Baraibar, S. Bojarra, C. Mügge, S. Schmidt, M. Nowaczyk, R. Kourist, *Catalysts* **2017**, *7*, 240.

[30] I. A. Mirza, B. J. Yachnin, S. Wang, S. Grosse, H. Bergeron, A. Imura, H. Iwaki, Y. Hasegawa, P. C. Lau, A. M. Berghuis, *J. Am. Chem. Soc.* **2009**, *131*, 8848-8854.

[31] N. A. Donoghue, P. W. Trudgill, *Eur. J. Biochem.* **1975**, *60*, 1-7.

[32] J. C. Meeks, R. W. Castenholz, *Arch Mikrobiol* **1971**, *78*, 25-41.

[33] C. K. Winkler, S. Simić, V. Jurkaš, S. Bierbaumer, L. Schmermund, S. Poschenrieder, S. A. Berger, E. Kulterer, R. Kourist, W. Kroutil, *ChemPhotoChem* **2021**.

[34] K. Koninger, A. Gomez Baraibar, C. Mugge, C. E. Paul, F. Hollmann, M. M. Nowaczyk, R. Kourist, *Angew. Chem., Int. Ed.* **2016**, *55*, 5582-5585.
[35] C. Szolkowy, L. D. Eltis, N. C. Bruce, G. Grogan, *ChemBioChem* **2009**, *10*, 1208-1217.
